# Effect of preconception low dose aspirin on pregnancy and live birth according to socioeconomic status: A secondary analysis of a randomized clinical trial

**DOI:** 10.1371/journal.pone.0200533

**Published:** 2019-04-18

**Authors:** Shilpi Agrawala, Lindsey A. Sjaarda, Ukpebo R. Omosigho, Neil J. Perkins, Robert M. Silver, Sunni L. Mumford, Matthew T. Connell, Ashley I. Naimi, Lisa M. Halvorson, Enrique F. Schisterman

**Affiliations:** 1 University of Texas Southwestern Medical Center, Dallas, TX, United States of America; 2 Epidemiology Branch, Division of Intramural Population Health Research, *Eunice Kennedy Shriver* National Institute of Child Health and Human Development, National Institutes of Health, Bethesda, MD, United States of America; 3 Department of Obstetrics and Gynecology, University of Utah and Intermountain Healthcare, Salt Lake City, UT, United States of America; 4 Program of Reproductive and Adult Endocrinology, *Eunice Kennedy Shriver* National Institute of Child Health and Human Development, National Institutes of Health, Bethesda, MD, United States of America; 5 Department of Epidemiology, University of Pittsburgh Graduate School of Public Health, Pittsburgh, PA, United States of America; 6 Gynecologic Health and Disease Branch, Division of Extramural Research, *Eunice Kennedy Shriver* National Institute of Child Health and Human Development, National Institutes of Health, Bethesda, MD, United States of America; TNO, NETHERLANDS

## Abstract

Low socioeconomic status (SES) is associated with adverse pregnancy outcomes and infertility. Low-dose aspirin (LDA) was shown to improve livebirth rates in certain subsets of women, and therefore, may impact pregnancy rates differentially by SES status. Therefore, the aim of the current study was to examine whether daily preconception-initiated LDA affects rates of pregnancy, livebirth, and pregnancy loss differently across strata of socioeconomic status (SES). This is a secondary analysis of The Effects of Aspirin in Gestation and Reproduction (EAGeR) Trial, a multisite, block- randomized, placebo-controlled trial conducted at four U.S. medical centers (n = 1,228, 2007–2012). Women attempting spontaneous conception with a history of pregnancy loss were randomly allocated preconception to 81mg of aspirin + 400mcg of folic acid (n = 615) or placebo + 400mcg of folic acid (n = 613). Study medication was administered for six menstrual cycles or until 36 weeks’ gestation if pregnancy was achieved. For this analysis, women were stratified by SES, which included income (low, mid, high) and a combined grouping of education and income (low-low, low-high, high-low, high-high). Log binomial models with robust variance estimated risks of pregnancy, livebirth, and pregnancy loss for LDA versus placebo. LDA increased pregnancy and livebirth rates (RR 1.23, 95% CI: 1.03, 1.45) in the high-income, but not mid- or low-income groups. LDA increased pregnancy rates in both the low education-low income group (RR 1.22, 95% CI: 1.02, 1.46) and the high education-high income group (RR 1.23, 95%CI: 1.06, 1.42), with no effect observed in mid-SES groupings. LDA, a low-cost and widely available treatment, may be particularly beneficial to women at the highest and lowest ends of the socioeconomic spectrum, though underlying mechanisms of this disparity are unclear. Confirming these findings and identifying factors which may modulate the effectiveness of LDA will ultimately facilitate personalized clinical care and improvements in population-level reproductive health.

**Trial registration number**: ClinicalTrials.gov, NCT00467363.

## Introduction

Low socioeconomic status (SES) is associated with higher rates of all-cause mortality [1, 2], as well as adverse pregnancy outcomes such as preterm birth [3–5] and low birth weight [6–8]. Multiple factors may contribute to higher rates of health complications in low SES populations, including lower medication compliance [9], restricted access to health care resources [10, 11], and more frequent chronic low-grade inflammation [12]. Data from the National Survey for Family Planning suggest that women with lower income and education have higher rates of infertility [13, 14], though this has not been validated by prospective cohort studies.

Low-dose aspirin (LDA) may improve implantation rates in women undergoing infertility treatments [15–17]. Furthermore, among women attempting spontaneous conception as part of the Effect of Aspirin in Gestation and Reproduction (EAGeR) trial, daily LDA initiated prior to conception improved live birth rates among a prespecified subgroup of women with a single recent pregnancy loss, but not the overall cohort [18]. Given the different effects of LDA on varied subgroups, we explored whether the effect of LDA was modulated by SES in the EAGeR trial. Indeed, because of the links between SES and medication compliance [9, 19], inflammation [20, 21], and healthcare access [11, 22], it is important to evaluate if the effect of LDA is influenced by socioeconomic conditions. Furthermore, LDA may be particularly beneficial to low SES women who lack the financial resources for more expensive interventions to achieve optimal reproductive outcomes.

Therefore, the aim of the present investigation was to examine the effect of daily preconception-initiated LDA compared to placebo on pregnancy, live birth, and pregnancy loss, stratified by different levels of income and education.

## Methods

The EAGeR trial was a multi-center, block-randomized, double-blind, placebo-controlled clinical trial conducted at four U.S. medical centers, enrolling a total of 1,228 women (2007–2012). Each site obtained approval from their Institutional Review Board (IRB), (Intermountain Healthcare IRB, Colorado Multiple IRB, University at Buffalo Health Sciences IRB, and The Wright Center for Graduate Medical Education IRB), and each IRB approved the intervention as an acceptable risk to the fetus. All participants provided written informed consent. A Data- Safety and Monitoring Board (DSMB) monitored patient safety and were informed of adverse events by a blinded committee. The trial was registered with ClinicalTrials.gov (#NCT00467363) where *a priori* primary and secondary trial outcomes are listed; the current investigation stratifying by income and education was not a preplanned aim of the parent trial. The full EAGeR trial study design and participant characteristics [23], as well as primary trial findings [18], were previously reported. All randomized trial participants (n = 1228) with income and education data available, and with completed follow-up, were included in this secondary analysis (n = 1087).

### Study design and population

Women aged 18–40 years who were actively attempting to conceive were eligible if they had regular menstrual cycles of 21–42 days in length, no known history of infertility, and one to two confirmed prior pregnancy losses. Women were excluded if they had any major medical problem such as diabetes or hypertension or any history of infertility. Women were also excluded if they had a contraindication to aspirin or any indication for anticoagulant therapy. Women were enrolled into one of two eligibility strata based on specific eligibility qualifications [18]: 1) original stratum: women with a history of exactly one pregnancy loss at less than 20 weeks’ gestation within the past 12 months; and 2) expanded stratum: women with a history of up two previous pregnancy losses of any gestational age at any time in the past.

### Treatment and study procedures

Participants were block randomized by study center and eligibility strata to receive daily LDA (81 mg) plus folic acid (400 mcg) (n = 615) or placebo plus folic acid (400 mcg) (n = 613). Treatment was assigned by the data-coordinating center using a computerized randomization algorithm; the participants, study staff, clinicians, and investigators were blinded to treatment throughout the trial. Study staff conducted enrollment. Pills were taken daily for up to six menstrual cycles while attempting to conceive and until 36 weeks’ gestation for those who became pregnant. Participants attended two scheduled clinic visits per cycle for the first two menstrual cycles (one scheduled on day 2–4 of the cycle and the other around expected ovulation) and a single visit per cycle on cycle day 2–4 thereafter. Both treatments utilized fertility monitors to assist with the timing of intercourse and the scheduling of clinic visits (Clearblue Easy Fertility Monitor: Inverness Medical). At study visits, pill bottles were weighed to calculate the percent of days compliant during the preconception treatment period.

Study participation ended when a woman completed six menstrual cycles without becoming pregnant. Women with an ongoing pregnancy were followed monthly through their pregnancy outcome (e.g. birth, etc.) and pregnant women continued their assigned treatment through 36 weeks’ gestation.

### Exposure assessment

Participants completed extensive questionnaires regarding household income, education level, ethnicity, parental education, occupational history, cigarette and substance use, and exercise. To characterize SES, the population was distributed into three income categories [low ≤ $39,999 (n = 406), mid $40,000-$99,999 (n = 330), high ≥ $100,000 (n = 491)]. Participants were also stratified by the combination of both income and education, selecting cut-points of education and income which dichotomized the education and income variables to achieve four relatively equivalent group sizes to permit similar power for detecting an effect of LDA on outcomes: 1) lower education-lower income (low-low) was defined as education of an Associate’s degree or lower and income ≤ $74,999 (n = 371); lower education-higher income (low-high) was defined as education of an Associates’ degree or lower and income >$74,999 (n = 307); higher education-lower income (high-low): education of Bachelors’ degree or higher and income ≤ $74,999 (n = 215); and higher education-higher income (high-high): education of a Bachelors’ degree or higher and income>$74,999 (n = 333).

Participants also underwent a thorough history and physical examination at the baseline visit (prior to randomization to LDA or placebo) including blood collection. Trained study staff measured body weight and height; body mass index (BMI) was calculated as weight (kg) divided by the square of height (m^2^) and is reported in kg/m^2^. The categorization of BMI was made as follows: underweight (UW) BMI <18.5 kg/m^2^; normal weight (NW) 18.5 kg/m^2^ ≤ BMI < 25 kg/m^2^; overweight-obese (OW-OB) BMI ≥ 25 kg/m^2^. High sensitivity C-reactive protein (hsCRP) was measured in serum samples collected at the baseline study visit (pre- randomization, day 2–4 of menses). An immunoturbidimetric assay using a Roche COBAS 6000 autoanalyzer was utilized to measure hsCRP to a limit of detection of 0.15 mg/L (Roche Diagnostics, Indianapolis, IN). Interassay coefficients of variation were 5.1% at 1.05 mg/L and 6.7% at 3.12 mg/L. hsCRP values ≥10.0 mg/L were excluded (n = 63, 5.1%) as this level is consistent with acute infection or injury [24, 25].

### Outcome measures

Primary outcomes for this analysis were hCG detected pregnancy, clinically confirmed pregnancy (gestational sac on ultrasound, clinical recording of fetal heart tones, or a later-stage confirmation of pregnancy), and live birth. An hCG detected pregnancy was determined from a positive result on a “real-time” urine pregnancy test (Quidel Quickvue, Quidel Corporation, San Diego, CA), which was sensitive to 25 mIU/ml hCG, conducted each time participants reported missing menses on any study visit timed to expected day 2–4 of the menstrual cycle; or from batched urine hCG testing performed after study completion on stored samples from the last 10 days of each woman’s first and second cycle of study participation (using daily first-morning urine collected and stored frozen at home) [18, 23] and on spot urine samples collected at all post-cycle visits (n = 21 additional pregnancies detected) [26].

Secondary outcomes were any pregnancy loss (pregnancy loss after either hCG detected pregnancy or clinically confirmed pregnancy) and clinical pregnancy loss (losses occurring after clinical confirmation of pregnancy).

### Statistical analysis

**Fig 1:**
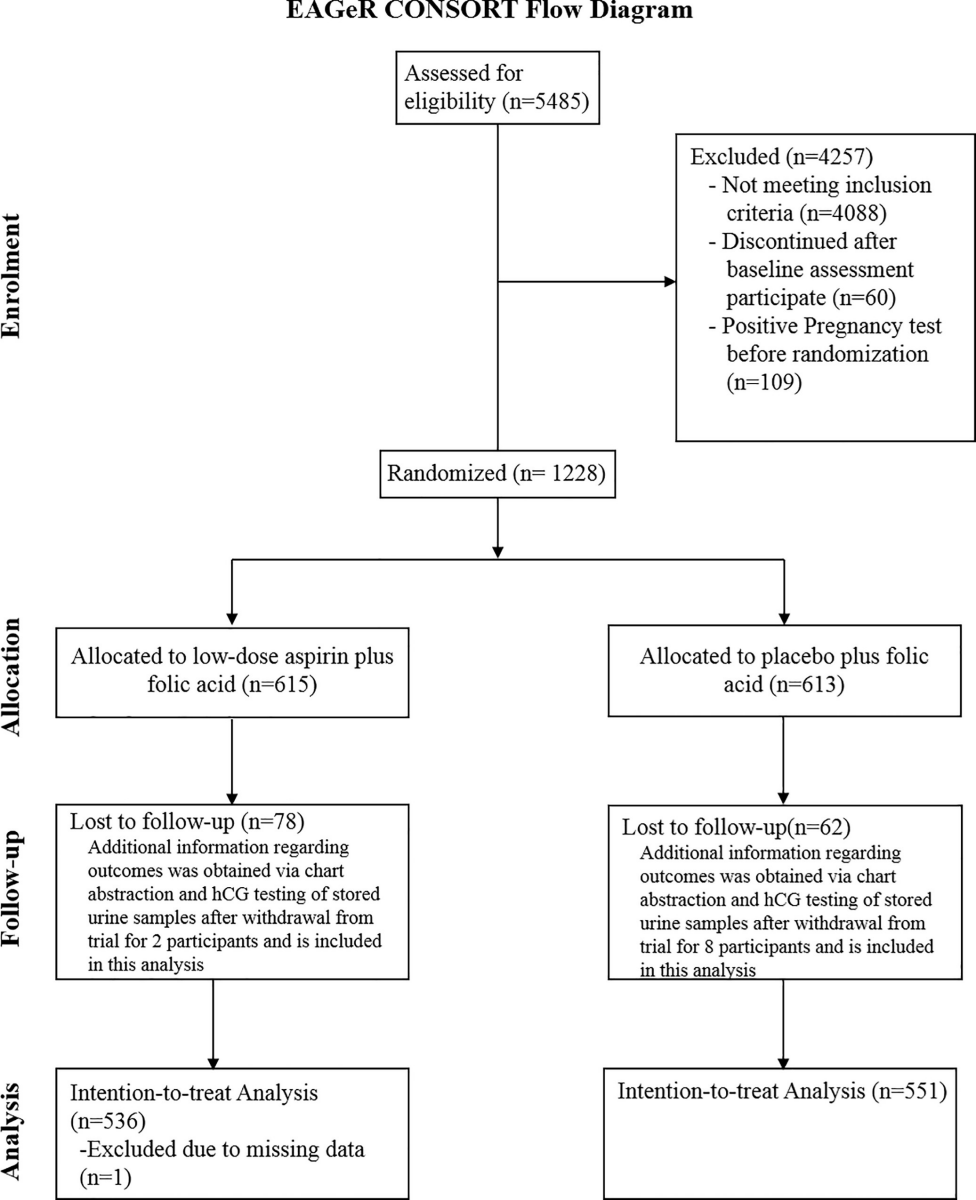
Participant flow for EAGeR Trial.

All analyses followed the intent-to-treat principle in that analyses were completed according to assigned treatment and no exclusions were made based on treatment compliance. 1,087 women were included in analyses (Fig 1) which encompassed all women who completed the trial (n = 1,078) and 9 additional women with complete income and education data for whom secondary outcome data relevant to the present analysis was obtained via chart abstraction and urine hCG testing [26]. All differences across SES groups in baseline characteristics were calculated using chi-square test. Log binomial models were used to estimate the risk ratio of LDA versus placebo for outcomes of hCG detected pregnancy, clinically confirmed pregnancy, live birth, and pregnancy loss. Analyses were stratified by income and the combination of education and income groupings described above. Inverse probability weights were employed in analyses of pregnancy loss and live birth to account for potential bias attributable to the effect of LDA on becoming pregnant. Because treatment allocation was randomized, all models evaluating the effect of LDA are reported without adjustment for any covariates. Sensitivity analyses adjusting for insurance status and hsCRP (variables observed to differ between treatment groups within some SES levels), as well as models additionally adjusting for age, BMI, race, and student status, were conducted to assess the robustness of the findings. All analyses were conducted in SAS version 9.4 (SAS Institute, Cary, NC, USA).

## Results

Participant flow is shown in Fig 1. Trial recruitment took place between 15 June 2007 and 15 July 2011 and follow-up continued through 2012. Of the four study centers, over 80% of women were from the Salt Lake City, UT area and the remainder were from Pennsylvania, Colorado, and upstate New York.

### Baseline characteristics

Baseline characteristics were similar between treatment arms, as expected from randomization, except that within the low-income group, women in the placebo group were more likely to have health insurance than women assigned to LDA. Also, within the mid-income group, women in the placebo group had a higher baseline hsCRP than the LDA group (Table 1).

**Table 1:** Participant characteristics by treatment arm and income level.

Characteristics	Overall Trial	Low Income: ≤ $39,999		Mid Income: $40,000-$99,999		High Income: ≥ $100,000	
		LDA	Placebo	LDA	Placebo	LDA	Placebo
	N = 1228^a^	N = 198	N = 208	N = 175	N = 155	N = 241	N = 250
Age, y	28.7 (4.8)	26.2 (4.5)	26.3 (4.3)	31.9 (4.4)	31.5 (3.9)	28.6 (4.1)	28.9 (4.6)
BMI kg/m^2^	26.3 (6.6)	27.6 (8)	27.1 (7)	24.9 (5.8)	25.8 (5.9)	26 (6)	26.3 (6.2)
% UW/NW/OW-OB	3.5/48.9/47.6	4.1/43.1/52.8	5.9/40.7/53.4	1.2/65.1/33.7	2.6/47.1/50.4	2.1/53.6/44.4	4.5/45.5/50.0
Waist:hip ratio	0.81 (0.07)	0.82 (0.07)	0.82 (0.08)	0.8 (0.06)	0.8 (0.06)	0.81 (0.08)	0.81 (0.07)
CRP mg/L (Geometric mean and std)	1.1 (2.9)	1.3 (3)	1.1 (3)	1 (3)	1.3 (2.9)	1.1 (2.8)	1.2 (2.8)
White (vs. non-white) race	1162 (94.6)	179 (90.4)	191 (91.8)	166 (94.9)	150 (96.8)	230 (95.4)	245 (98)
Marital status: married or living with partner (vs. other)	1198 (97.6)	190 (96)	193 (92.8)	175 (100)	154 (99.4)	240 (99.6)	245 (98)
Education:							
< HS/HS/GED	182 (14.8)	54 (27.4)	50 (24)	11 (6.3)	9 (5.8)	30 (12.4)	28 (11.2)
Some college, no degree & Associates	497 (40.5)	95 (48.2)	98 (47.1)	41 (23.4)	41 (26.5)	110 (45.6)	111 (44.4)
Bachelors (BA, Ab, BS, BBS)	394 (32.1)	41 (20.8)	55 (26.4)	72 (41.1)	61 (39.4)	76 (31.5)	89 (35.6)
Masters, professional school degree(MD/JD), doctoral degree	154 (12.6)	7 (3.6)	5 (2.4)	51 (29.1)	44 (28.4)	25 (10.4)	22 (8.8)
Student, Yes	185(11)	35 (17.8)	50 (24)	16 (9.1)	13 (8.4)	35 (14.5)	36 (14.4)
Health Insurance, Yes	1089 (88.9)	137 (69.2)	167 (80.3)	170 (97.1)	152 (99.3)	229 (95.4)	233 (93.2)
Employment:							
Not employed	276 (23.3)	63 (33.9)	60 (30.6)	28 (16.3)	20 (13.2)	46 (19.6)	59 (24.2)
Part-time	287 (24.2)	57 (30.6)	47 (24)	34 (19.8)	28 (18.5)	64 (27.2)	57 (23.4)
Full time	608 (51.4)	65 (34.9)	86 (43.9)	109 (63.4)	101 (66.9)	122 (51.9)	125 (51.2)
Other	13 (1.1)	1 (0.5)	3 (1.5)	1 (0.6)	2 (1.3)	3 (1.3)	3 (1.2)
Smoke while pregnant, Yes	105 (9.3)	20 (11.8)	22 (11.8)	14 (8.4)	17 (11.9)	14 (6.2)	18 (7.6)
Drink while pregnant, Yes	52 (4.7)	9 (5.3)	12 (6.7)	10 (6.3)	11 (7.8)	3 (1.4)	7 (3)
Exercise per week							
%Low/Moderate/High	26.2/40.7/33	25.9/38.6/35.5	26.9/34.6/38.5	25.7/43.4/30.9	25.8/49.7/24.5	24.5/43.2/32.4	28.4/38/33.6
Prior live birth							
0	571 (46.5)	93 (47)	107 (51.4)	81 (46.3)	71 (45.8)	109 (45.2)	110 (44)
1	443 (36.1)	70 (35.4)	75 (36.1)	65 (37.1)	58 (37.4)	85 (35.3)	89 (35.6)
2	214 (17.4)	35 (17.7)	26 (12.5)	29 (16.6)	26 (16.8)	47 (19.5)	51 (20.4)
Number of previous pregnancy losses							
1	825 (67.2)	141 (71.2)	141 (67.8)	118 (67.4)	105 (67.7)	162 (67.2)	157 (62.8)
2	403 (32.8)	57 (28.8)	67 (32.2)	57 (32.6)	50 (32.3)	79 (32.8)	93 (37.2)
Time from last loss to randomization							
≤ 4 months	651 (53.8)	106 (53.8)	112 (54.1)	109 (63.4)	81 (52.9)	115 (49.4)	127 (51.6)
5–8 months	222 (18.4)	38 (19.3)	37 (17.9)	21 (12.2)	29 (19)	44 (18.9)	53 (21.5)
9–12 months	99 (8.2)	16 (8.1)	18 (8.7)	14 (8.1)	15 (9.8)	20 (8.6)	16 (6.5)
>12 months	237 (19.6)	37 (18.8)	40 (19.3)	28 (16.3)	28 (18.3)	54 (23.2)	50 (20.3)

UW, underweight (BMI<18.5); NW, normal weight (BMI 18.5-<25); OW-OB, overweight-obese (BMI ≥25).

^a^ Income subgroups total 1227 participants because one woman was missing income data

### Effects on pregnancy and live birth

When stratified by income alone, only the highest income women (≥ $100,000) had a significant increase in live birth among those assigned to LDA compared to placebo 59% (129/217) vs. 49% (115/237); (RR 1.23, 95% CI: 1.03, 1.45, Table 2). Results were similar for hCG detected and clinical pregnancy with approximately 14% increased pregnancy rates in the highest income group (Table 2). After stratifying by the combination of both education and income (low education-low income, low education-high income; high education-low income, high education-high income), the high-high group assigned to LDA had a significantly higher clinical pregnancy rate of 77% (124/161) compared to 63% (98/156) in the placebo group (RR 1.23, 95% CI: 1.06, 1.42, Table 3). However, among this same group, the effect on live birth was attenuated (RR 1.17, 95% CI: 0.97,1.41, Table 3). In addition, the low-low group assigned to LDA had a significantly higher clinical pregnancy rate compared to placebo 68% (103/151) vs. 56% (81/145); (RR 1.22, 95% CI: 1.02, 1.46, Table 3), and the effect estimate was similar, but less precise for live birth (RR 1.23 95% CI: 0.99, 1.54, Table 3). There was no effect of LDA on pregnancy or live birth among mid-SES categories (Tables 2 and 3).

**Table 2:** Effect of low-dose aspirin (LDA) treatment versus placebo on pregnancy and live birth incidence stratified by income.

	hCG detected pregnancy		Clinically confirmed pregnancy^§^		Live birth	
	LDA	Placebo	LDA	Placebo	LDA	Placebo
**P-value for interaction by income level**	0.65		0.68		0.42	
**All women (N = 1087)**	536	551	536	551	536	551
Achieved outcome–no. (%)	405 (75.4)	380 (69.0)	374 (69.6)	350 (63.5)	309 (57.5)	288 (52.3)
Low Income, < $39,999						
No. of participants	163	169	163	169	163	169
Achieved outcome–no. (%)	119 (73.0)	116 (68.6)	108 (66.3)	108 (63.9)	89 (54.6)	90 (53.3)
Risk Ratio (95 CI)	1.06 (0.93,1.22)		1.04 (0.89,1.21)		1.03 (0.84,1.25)	
Mid Income, $40,000-$99,000						
No. of participants	156	145	156	145	156	145
Achieved outcome–no. (%)	122 (78.2)	107 (73.8)	114 (73.1)	96 (66.2)	91 (58.3)	83 (57.2)
Risk Ratio (95 CI)	1.06 (0.93,1.2)		1.10 (0.95,1.28)		1.02 (0.84,1.24)	
High Income, ≥ $100,000						
No. of participants	217	237	217	237	217	237
Achieved outcome–no. (%)	164 (75.6)	157 (66.2)	152 (70.0)	146 (61.6)	129 (59.4)	115 (48.5)
Risk Ratio (95 CI)	1.14 (1.01,1.28) ^†^		1.14 (1.00,1.30)		1.23 (1.03,1.45) ^†^	

^†^p<0.05

^§^Pregnancy identified by 6–7 week ultrasound

**Table 3:** Effect of low-dose aspirin (LDA) treatment versus placebo on pregnancy and live birth incidence stratified by education-income.

	hCG detected pregnancy		Clinically confirmed pregnancy^§^		Live birth	
	LDA	Placebo	LDA	Placebo	LDA	Placebo
**P-value for interaction by education-income**	0.07		0.04		0.59	
**All women (N = 1087)**	536	551	536	551	536	551
Achieved outcome–no. (%)	405 (75.4)	380 (69.0)	374 (69.6)	350 (63.5)	309 (57.5)	288 (52.3)
low- low						
No. of participants	151	145	151	145	151	145
Achieved outcome–no. (%)	112 (74.2)	90 (62.1)	103 (68.2)	81 (55.9)	86 (57.0)	67 (46.2)
Risk Ratio (95 CI)	1.19 (1.02,1.40) ^†^		1.22 (1.02,1.46) ^†^		1.23 (0.99,1.54)	
low- high						
No. of participants	134	142	134	142	134	142
Achieved outcome–no. (%)	95 (70.9)	100 (70.4)	86 (64.2)	93 (65.5)	74 (55.2)	72 (50.7)
Risk Ratio (95 CI)	1.01 (0.86,1.17)		0.98 (0.82,1.17)		1.09 (0.87,1.36)	
high-low						
No. of participants	90	108	90	108	90	108
Achieved outcome–no. (%)	66 (73.3)	83 (76.9)	61 (67.8)	78 (72.2)	49 (54.4)	66 (61.1)
Risk Ratio (95 CI)	0.95 (0.81,1.12)		0.94 (0.78,1.13)		0.89 (0.70,1.13)	
high- high						
No. of participants	161	156	161	156	161	156
Achieved outcome–no. (%)	132 (82.0)	107 (68.6)	124 (77)	98 (62.8)	100 (62.1)	83 (53.2)
Risk Ratio (95 CI)	1.20 (1.05,1.36)*		1.23 (1.06,1.42)*		1.17 (0.97,1.41)	

*P≤0.01

^†^P<0.05, from log binomial models evaluating the effect of LDA vs. placebo within each education-income group.

^§^Pregnancy identified by 6–7 week ultrasound

Overall preconception compliance to assigned treatments was 88%, 90%, and 89% of days compliant for low, mid, and high-income categories, respectively. Preconception percent days compliant ranged from 87 to 91% for the four education-income stratification groups corresponding to Table 3. There were no significant compliance differences between treatment and placebo groups. Similarly, analyses of interactions of compliance with all SES categories produced no evidence of significant differentiation.

### Effects on pregnancy loss

LDA did not significantly affect pregnancy loss in any group, whether women were stratified by income alone or the combination of education and income (Table 4).

**Table 4:** Effect of low-dose aspirin (LDA) treatment versus placebo on pregnancy losses.

	Any Pregnancy Loss		Clinical Pregnancy Loss	
	Among women with any pregnancy (N = 785)		Among women with clinically confirmed pregnancy (n = 724)	
	LDA	Placebo	LDA	Placebo
	405	380	374	350
Achieved outcome–no. (%)	96 (23.7)	92 (24.2)	65 (17.4)	62 (17.7)
**Income**				
**P-value for interaction by income level**	0.42		0.20	
Low Income				
No. of participants	119	116	108	108
Achieved outcome–no. (%)	30 (25.2)	26 (22.4)	19 (17.6)	18 (16.7)
Risk Ratio (95 CI)	1.09 (0.69, 1.71)		1.01 (0.57, 1.80)	
Mid Income				
No. of participants	122	107	114	96
Achieved outcome–no. (%)	31 (25.4)	24 (22.4)	23 (20.2)	13 (13.5)
Risk Ratio (95 CI)	1.18 (0.75, 1.86)		1.59 (0.85, 2.96)	
High income				
No. of participants	164	157	152	146
Achieved outcome–no. (%)	35 (21.3)	42 (26.8)	23 (15.1)	31 (21.2)
Risk Ratio (95 CI)	0.81 (0.55, 1.20)		0.74 (0.46, 1.19)	
**Education-Income**				
**P-value for interaction by education-income**	0.59		0.30	
Low- low				
No. of participants	112	90	103	81
Achieved outcome–no. (%)	26 (23.2)	23 (25.6)	17 (16.5)	14 (17.3)
Risk Ratio (95 CI)	0.89 (0.55,1.44)		0.91 (0.49, 1.71)	
Low- high				
No. of participants	95	100	86	93
Achieved outcome–no. (%)	21 (22.1)	28 (28.0)	12 (14.0)	21 (22.6)
Risk Ratio (95 CI)	0.79 (0.48,1.3)		0.61 (0.31, 1.18)	
High-low				
No. of participants	66	83	61	78
Achieved outcome–no. (%)	17 (25.8)	17 (20.5)	12 (19.7)	12 (15.4)
Risk Ratio (95 CI)	1.22 (0.68, 2.21)		1.27 (0.61, 2.65)	
High- high				
No. of participants	132	107	124	98
Achieved outcome–no. (%)	32 (24.2)	24 (22.4)	24 (19.4)	15 (15.3)
Risk Ratio (95 CI)	1.15 (0.73, 1.81)		1.40 (0.77, 2.52)	

From log binomial models evaluating the effect of LDA vs. placebo within each group

### Sensitivity analyses

Models including a random effect of site, those adjusting for insurance and hsCRP, and those additionally adjusting for age, BMI, race, and student status, produced similar effect estimates and identical patterns of statistical significance across the groups and outcomes.

### Adverse events or side effects in intervention

There was greater vaginal bleeding among the participants in the treatment arm; however, greater bleeding was not associated with any adverse pregnancy outcome. LDA therapy was generally well tolerated and these results have been previously reported in detail [18, 27].

## Discussion

In this preliminary investigation, women of higher SES consistently benefited from preconception LDA therapy, whether defined by income alone or the combination of education and income. Women with higher income had a 23% increase in live birth rate when taking daily LDA preconception until 36 weeks of pregnancy, and women attaining a Bachelor’s degree or higher and household income ≥$75,000 had a 23% increase in clinical pregnancy rate attributable to preconception LDA. Furthermore, women with the combination of both lower income and lower education benefited from LDA with an increase of clinical pregnancy rates of 22%, but LDA was not associated with better outcomes in women with only lower income. No benefit of LDA was observed among groups characterized by relatively middle income and education. These unexpected findings suggest that LDA may uniquely improve pregnancy and live birth rates among women at the low and high ends of the SES spectrum, though these results require replication.

There are various mechanisms through which LDA may improve pregnancy and live birth rates. It may be that some of these mechanisms operate more in low or high SES women, thus producing the results observed here in either end of the SES spectrum. LDA may promote implantation [16], through improved blood flow at the implantation site [28], upregulated cell adhesion molecules on the endometrial surface [29], increased systemic concentrations of prostacyclin, which relaxes smooth muscle and dilates blood vessels, decreased platelet aggregation [30], as well as increased ovarian and uterine blood flow [17]. These mechanisms have been proposed to explain the increased pregnancy rates observed in other studies [15, 31]. However, it is unclear how any of these mechanisms may differ in low or high SES populations.

Higher education and higher income are associated with increased rates of medication compliance [19]. An example of this is oral contraception where women with the lowest income and lowest education have the lowest compliance [9, 32, 33]. However, we found no difference in estimated compliance across SES groups. Thus, variance in compliance is unlikely to explain our findings. It may be that the lower SES groupings in the present study were still higher than in prior studies, limiting any impacts of SES on compliance behavior, given the overall affluence of the EAGeR study population. Alternatively, women seeking pregnancy after experiencing a pregnancy loss may be particularly motivated to adhere to medication they perceive may improve their pregnancy chances.

Another potential factor, which may explain differential effects of LDA by SES, is access to health care resources and underdiagnosed preexisting conditions. For example, in the present study, the high-income group was more likely to have health insurance (94% vs. 75%) than the low-income group. Women with health insurance have increased access to care and are more likely to seek infertility care, thereby increasing the likelihood of having certain sub-fertility conditions identified compared to women without health insurance [34]. Since any history of sub-fertility or infertility or planned use of fertility treatments was among the exclusion criteria in the EAGeR trial, it is possible that there was greater undetected infertility or sub-fertility among women of lower SES, which could have attenuated the effects of LDA. Thus, it is possible that undetected subfertility conditions may have attenuated the effect of LDA among women with the lowest income (<$40,000, representing the low income alone group) where no effect of LDA was observed here and insured rates were lower.

Lastly, SES is an independent risk factor for a chronic inflammatory state. Although poor health behaviors such as smoking, obesity, and alcohol use may contribute, they do not appear to fully account for the link between low SES and inflammation [20, 21, 35, 36]. LDA was first used to modulate inflammation in cardiovascular disease [37, 38], but LDA can also lower inflammation and increase live birth rates in women with a baseline higher hsCRP as we have previously reported [39]. This modulation of inflammation in a low SES population may explain the increased pregnancy rate in the low-low group. A significant increase in live birth rate, however, was only seen in women with high hsCRP and normal BMI previously, and while our low-low group had a somewhat higher baseline hsCRP, its participants also had higher BMIs (S1 Table), which may have attenuated the effect of LDA. Another consideration is whether LDA significantly impacted pregnancy loss in our analysis. An LDA-mediated improvement in clinically confirmed pregnancy rates without an associated increase in live birth rates (Table 3) might intuitively suggest an increased pregnancy loss rate. However, there were no effects of LDA observed on pregnancy loss when stratified by either income or the combination of education and income. The small number of pregnancy losses within the stratified groups limits our ability to interpret these data, but these findings are consistent with the main trial findings reporting no effect of LDA on pregnancy loss overall, by eligibility strata, or by loss subtype [18, 26].

The strength of our study is that this is the first major trial that prospectively recorded hCG pregnancy rates, clinical pregnancy rates, and live birth rates and included the SES of its participants seeking spontaneous conception. Prior studies have included participants’ SES in the setting of fertility treatment [22, 40], but have not included SES categorization in clinical infertility trials. One of the limitations of this study is generalizability because participants were on average more educated and had higher income compared to the US population; it is possible that outcomes may differ if studied in populations with greater socioeconomic variation. Furthermore, the highest income group unexpectedly displayed a lower distribution of education level (‘Some college, no degree’ was most common) and lesser full-time employment, coupled with a somewhat greater proportions of students, as compared to the middle income group. Since students were instructed to report household income, which could include spousal income, representation of younger college students may be higher in the highest income group compared to studies of other populations. It remains important for future trials to include women of all socioeconomic backgrounds to enable further assessment of the impact of SES on modulating treatment effectiveness. Lastly, the possibility of identifying false positive findings (i.e. type I error), due in part to multiple, stratified testing, must be acknowledged [41].

Overall, our findings indicate that LDA increased pregnancy and live birth rates in women with high income and also increased hCG and clinical pregnancy rates in women with the combination of either low education/low income or high income/high education. However, no effects of LDA were observed among middle income and middle education/income women. Different underlying mechanisms may enable a greater effectiveness of daily preconception LDA therapy for women at either end of the SES spectrum, but the specific underpinnings of these differential effects, as well as SES thresholds applicable to less affluent populations, remain unresolved. Given this lack of clarity for mechanisms underlying these differential effects, as well as the possibility of identifying false positive results due to multiple, stratified testing, it is also possible the observed effects are spurious. A continued effort to confirm the present findings and understand the various factors which may modulate the effectiveness of LDA, a low-cost and widely available treatment, for reproductive outcomes remains critical to ultimately enable both personalized clinical care as well as improvements in population-level reproductive health.

## Supporting information

S1 Table Participant characteristics by treatment arm and education-income. (DOCX)

S1 File CONSORT checklist. (DOC)

S2 File Study protocol. (PDF)
