# Supplementary material for: Effect of preconception low dose aspirin on pregnancy and live birth according to socioeconomic status: A secondary analysis of a randomized clinical trial
Source: PLoS One. 2019 Apr 18;14(4):e0200533. doi: 10.1371/journal.pone.0200533 (PMC6472730; doi:10.1371/journal.pone.0200533)
Supplement: S1 Table — (DOCX) [file pone.0200533.s001.docx]

**S1 Table.** Participant characteristics by treatment arm and education-income.

| Characteristics |  | **Low- low** | | **Low- high** | | **High-low** | | **High- high** | |
| --- | --- | --- | --- | --- | --- | --- | --- | --- | --- |
|  | **Overall Trial** | **LDA** | **Placebo** | **LDA** | **Placebo** | **LDA** | **Placebo** | **LDA** | **Placebo** |
|  | **N=1228^a^** | **N=187** | **N=184** | **N=154** | **N=153** | **N=101** | **N=114** | **N=171** | **N=162** |
| Age, y | 28.7 (4.8) | 26.7 (5) | 26.6 (4.5) | 28.2 (4.4) | 28.2 (4.6) | 29.4 (3.8) | 29.2 (4.1) | 31.3 (4.4) | 31.2 (4.3) |
| BMI |  |  |  |  |  |  |  |  |  |
| kg/m^2^ | 26.3 (6.6) | 27.6 (8.2) | 27.3 (7.2) | 26.8 (6.5) | 26.7 (6.6) | 25.7 (5.8) | 26.1 (6.2) | 24.4 (5.2) | 25.5 (5.3) |
| % UW/NW/OW-OB | 3.5/48.8/47.7 | 3.2/43.8/52.9 | 5/39.7/55.4 | 2.6/46.4/51 | 6.8/41.2/52 | 3/54/43 | 4.4/46.5/49.2 | 1.2/70.1/28.8 | 1.9/50.6/47.6 |
| Waist:hip ratio | 0.81 (0.07) | 0.82 (0.07) | 0.82 (0.08) | 0.82 (0.08) | 0.81 (0.07) | 0.81 (0.06) | 0.81 (0.06) | 0.79 (0.07) | 0.81 (0.07) |
| CRP |  |  |  |  |  |  |  |  |  |
| mg/L (Geometric mean and std) | 1.1 (2.9) | 1.4 (3) | 1.2 (3) | 1.2 (2.8) | 1.3 (2.9) | 1.1 (2.9) | 1.1 (2.9) | 0.9 (2.9) | 1.1 (2.7) |
| % low/mid/  high | 33.5/33.1/  33.4 | 26.6/29/  44.4 | 32.7/28.5/  38.8 | 29.9/40.3/  29.9 | 32.1/30/  37.9 | 33.7/32.6/  33.7 | 34.9/38.5/  26.6 | 45.8/30.1/  24.1 | 32/38.6/  29.4 |
| White (vs. non-white) race | 1162 (94.6) | 168 (89.8) | 171 (92.9) | 147 (95.5) | 149 (97.4) | 97 (96) | 107 (93.9) | 162 (94.7) | 159 (98.1) |
| Marital status: married or living with partner (vs. other) | 1198 (97.6) | 180 (96.3) | 171 (92.9) | 154 (100) | 150 (98) | 101 (100) | 112 (98.2) | 170 (99.4) | 159 (98.1) |
| Student, Yes | 185 (15) | 33 (17.6) | 43 (23.4) | 31 (20.1) | 25 (16.3) | 13 (12.9) | 16 (14) | 9 (5.3) | 15 (9.3) |
| Health Insurance, Yes | 1089 (88.9) | 131 (70.1) | 149 (81.4) | 144 (94.1) | 138 (90.2) | 92 (91.1) | 106 (93.8) | 168 (98.2) | 159 (98.1) |
| Employment: |  |  |  |  |  |  |  |  |  |
| Not employed | 276 (23.3) | 59 (33.5) | 50 (28.7) | 32 (21.1) | 38 (25.9) | 20 (20) | 20 (18) | 26 (15.8) | 31 (19.5) |
| Part-time | 287 (24.2) | 46 (26.1) | 41 (23.6) | 40 (26.3) | 35 (23.8) | 30 (30) | 22 (19.8) | 39 (23.6) | 34 (21.4) |
| Full time | 608 (51.4) | 71 (40.3) | 81 (46.6) | 78 (51.3) | 73 (49.7) | 49 (49) | 68 (61.3) | 98 (59.4) | 90 (56.6) |
| Smoke while pregnant, Yes | 105 (9.3) | 23 (14.2) | 22 (13.8) | 11 (7.7) | 17 (12.1) | 5 (5.3) | 9 (8.3) | 9 (5.6) | 9 (5.8) |
| Drink while pregnant, Yes | 52 (4.7) | 11 (6.8) | 13 (8.2) | 1 (0.7) | 8 (5.8) | 4 (4.3) | 2 (1.9) | 6 (3.9) | 7 (4.6) |
| Exercise per week  %Low/Moderate/High | 26.2/40.7/  33 | 26.9/34.9/  38.2 | 30.4/34.2/  35.3 | 24.7/40.9/  34.4 | 32/34.6/  33.3 | 20.8/46.5/  32.7 | 22.8/44.7/  32.5 | 26.9/47.4/  25.7 | 22.2/47.5/  30.2 |
| Prior live birth |  |  |  |  |  |  |  |  |  |
| 0 | 571 (46.5) | 89 (47.6) | 82 (44.6) | 63 (40.9) | 65 (42.5) | 43 (42.6) | 65 (57) | 87 (50.9) | 76 (46.9) |
| 1 | 443 (36.1) | 65 (34.8) | 72 (39.1) | 59 (38.3) | 58 (37.9) | 41 (40.6) | 35 (30.7) | 55 (32.2) | 57 (35.2) |
| 2 | 214 (17.4) | 33 (17.6) | 30 (16.3) | 32 (20.8) | 30 (19.6) | 17 (16.8) | 14 (12.3) | 29 (17) | 29 (17.9) |
| Number of previous pregnancy losses |  |  |  |  |  |  |  |  |  |
| 1 | 825 (67.2) | 130 (69.5) | 125 (67.9) | 101 (65.6) | 86 (56.2) | 69 (68.3) | 81 (71.1) | 120 (70.2) | 111 (68.5) |
| 2 | 403 (32.8) | 57 (30.5) | 59 (32.1) | 53 (34.4) | 67 (43.8) | 32 (31.7) | 33 (28.9) | 51 (29.8) | 51 (31.5) |
| Time from last loss to randomization |  |  |  |  |  |  |  |  |  |
| ≤ 4 months | 651 (53.8) | 99 (52.9) | 83 (45.6) | 75 (50) | 70 (46.4) | 63 (63.6) | 78 (69) | 92 (55.8) | 89 (55.6) |
| 5-8 months | 222 (18.4) | 35 (18.7) | 36 (19.8) | 31 (20.7) | 33 (21.9) | 12 (12.1) | 13 (11.5) | 25 (15.2) | 37 (23.1) |
| 9-12 months | 99 (8.2) | 17 (9.1) | 18 (9.9) | 11 (7.3) | 12 (7.9) | 8 (8.1) | 10 (8.8) | 14 (8.5) | 9 (5.6) |
| >12 months | 237 (19.6) | 36 (19.3) | 45 (24.7) | 33 (22) | 36 (23.8) | 16 (16.2) | 12 (10.6) | 34 (20.6) | 25 (15.6) |

UW, underweight (BMI<18.5); NW, normal weight (BMI 18.5-<25); OW-OB, overweight-obese (BMI ≥25).

^a^Education-income subgroups total 1226 participants because one woman was missing income data and another woman was missing education data.
