# Supplementary material for: Effect of preconception low dose aspirin on pregnancy and live birth according to socioeconomic status: A secondary analysis of a randomized clinical trial
Source: PLoS One. 2019 Apr 18;14(4):e0200533. doi: 10.1371/journal.pone.0200533 (PMC6472730; doi:10.1371/journal.pone.0200533)

*E*ffects of *A*spirin in *Ge*station and *R*eproduction
Study Protocol
Version 2.2: 09/10/07

The EAGeR Study is a randomized clinical trial with study centers at the University at Buffalo and the University of Utah and a Data Coordinating Center at the University of Haifa. The study is funded and organized by the National Institute of Child Health and Human Development, with Enrique F. Schisterman, PhD as the Project Officer.

For more information, go to the EAGeR study website:
http://www.EAGeRtrial.org

University at Buffalo Study Center
University at Buffalo—EAGeR Trial
Department of Social and Preventive Medicine
65 Farber Hall
Buffalo, NY 14214
(716) 829-3128
Principal Investigator: Jean Wactawski-Wende, PhD

University of Utah Study Center
University of Utah—EAGeR Trial
Department of Obstetrics and Gynecology
30 North 1900 East, 2B200
Salt Lake City, Utah 84132
(801) 581-4128
Principal Investigator: Robert Silver, MD

University of Haifa Data Coordinating Center
University of Haifa—EAGeR Trial
Department of Statistics
Mount Carmel
Haifa, Israel 31905
Principal Investigator: Noya Galai, PhD

Effects of Aspirin in Gestation and Reproduction (EAGeR)

Study Protocol

Table of Contents **Page**

A. Study Description and Objectives 3

B. Background 5

- B.1. Blood Flow and Reproduction 5
- B.2. Aspirin and Reproduction 7
- B.3. Aspirin and Fertility 9
- B.4. Aspirin and Pregnancy Loss 10
- B.5. Aspirin and Obstetric Complications 11
- B.6. Remaining Questions 13

C. Study Design 14

- C.1. Introduction 14
- C.2. Eligibility criteria 14
- C.3. Treatment groups 16
- C.4. Outcome measures 17

D. Participant Management 23

- D.1. Recruitment 23
- D.2. Study visits and contacts overview 23
- D.3. Screening Process 24
- D.4. Baseline study visit 25
- D.5. Randomization 26
- D.6. Active follow-up 28
- D.7. Passive follow-up 31
- D.8. Pregnancy follow-up 33
- D.9. Specimen collection 37
- D.10 Retention Plan and Compensation 38
- D.11 Sub-study 39

E. Study Timeline 40

F. Study Administration 41

G. Statistical Analysis 42

H. Data Management Procedures 49

I. Human Subject Protection and Confidentiality Procedures 55

J. References 62

K. Appendices 69

Figures

Figure 1. Participants' management in the EAGeR Trial	24
Figure 2. Timeline for participant follow-up during attempt to conceive	32
Figure 3 Timeline for participant follow-up during pregnancy	36
Figure 4. Probability of live birth versus implantation probability per menstrual cycle	44

Tables:

Table 1. Activities performed during pregnancy follow-up	36
Table 2. Study phases and activities by months from start of funding	40
**Table 3.** Power for comparing live birth rates in treatment and placebo groups with no interim looks.	45
**Table 4.** Power for one or two interim looks assuming	45

A. STUDY DESCRIPTION AND OBJECTIVES

Human reproduction is a remarkable but inefficient process. Maximally fertile couples have only an approximately 30 percent chance of conception in a given menstrual cycle, and average monthly fecundity is 20%. Successful conception does not guarantee a live birth. Estimates of miscarriages and early pregnancy loss vary and range between about 15 to 31%. Available data suggest that low dose aspirin (LDA) has the potential to favorably impact on several aspects of reproduction including conception, implantation, early pregnancy loss, late fetal death, preeclampsia, small for gestational age fetus, placental insufficiency and preterm birth. LDA is an ideal therapy; it is apparently safe, widely available, has few maternal side effects, and is inexpensive. However, much remains to be learned. It is unclear which patients would benefit from LDA, what the optimal dose and duration of treatment should be, whether mechanisms other than an influence on blood flow contribute to the effects of LDA, and what the adverse effects of different doses and durations of treatment might be. Prior studies have focused on narrow aspects of the effect of LDA such as IVF outcome or the development of preeclampsia. Reproduction is unique in that each stage is inextricably linked. Better blood flow at the time of conception and implantation may ultimately lead to improved placental function, and a reduced risk of preeclampsia, SGA, and preterm birth.

Unanswered questions about LDA and pregnancy can best be answered through a comprehensive, carefully designed, prospective randomized clinical trial. Observational epidemiological studies of endpoints such as these are often limited in their ability to properly evaluate implantation and early pregnancy loss as this requires a very careful daily monitoring of hormonal levels based on collection of daily samples of first morning urine, necessitating both a very committed population and a well designed system to help women to comply with this demanding regimen. As such, we propose a prospective randomized controlled trial to evaluate the "Effects of Aspirin in Gestation and Reproduction (EAGeR)," with the following objectives:

Study Objectives

The **primary objective** of the study is:

To determine the effect of low-dose aspirin (LDA) in combination with folic acid on the incidence of live births relative to placebo plus folic acid.

Secondary objectives will include:

a To determine the effect of LDA in combination with folic acid on the incidence of hCG detected pregnancy relative to placebo plus folic acid.

b To determine the effect of LDA in combination with folic acid on the incidence of clinically recognized pregnancy (by 6.5 week ultrasound) relative to placebo plus folic acid.

c To determine the effect of LDA in combination with folic acid on specific pregnancy outcomes (gestational age, preterm birth, birthweight [small for gestational age], major neonatal complications, length of hospital stay for infant, preeclampsia, and cost) relative to placebo plus folic acid.

Additional objectives are:

d To evaluate the safety of LDA in the participants and fetuses.

e To create a repository of tissue samples for future evaluation.

B. BACKGROUND

Infertility is a prevalent problem which is getting much attention and is causing a growing burden on health services in the western world. Estimates of miscarriages and early pregnancy loss vary and range between about 15 to 31%. A prospective observational study conducted in China reported that 8% of all detectable conceptions, ended in clinical spontaneous abortion, and 25% in early pregnancy loss. Of the cycles with chemically (but not clinically) detected pregnancies 14% ended in early loss.[1]

Maximally fertile couples only have an approximately 30% chance of conception in any given menstrual cycle,[2] and average monthly fecundity is estimated at 20%.[3] Successful conception does not guarantee a live birth. Over 30% of conceptions are lost early in gestation.[4] Many of these are due to problems with implantation and may not be clinically apparent. Additionally, 12–15% of conceptions result in clinically recognized pregnancy loss.[5]

The causes of subfertility or infertility, implantation failure, miscarriage, fetal death, and pregnancy complications are myriad and often poorly understood. For example, pregnancy loss may be due to genetic abnormalities, uterine malformations, hormonal abnormalities, immunologic disorders, as well as other causes.[6] In many cases an etiology is never determined. Nonetheless, a unifying feature of some cases of infertility, pregnancy loss, and obstetric complications is a decrease in uterine, ovarian, and placental blood flow.

B.1. Blood Flow and Reproduction

a. Fertility

Impaired uterine blood flow has been suggested as a cause for infertility.[7] This is supported by the observation that embryo implantation failure is higher in women undergoing in vitro fertilization (IVF) who have impaired uterine perfusion as measured by Doppler ultrasound.[8-9] Also, a relatively thin endometrial lining has been associated with decreased likelihood of successful pregnancy.[10] Since endometrial growth requires blood flow for delivery of oxygen, hormones and nutrients,[11] suboptimal endometrial growth and development may be a direct consequence of inadequate uterine perfusion. In turn, poor endometrial growth decreases the odds of successful implantation and pregnancy outcome. It is likely that impaired ovarian perfusion may also contribute to the risk of sub- or infertility. Ovarian perfusion is critical to many mechanisms in the process of ovulation, which is a critical step for reproduction.

b. Pregnancy Loss

Abnormal placental blood flow also has been implicated as a cause of both early and late pregnancy loss. The majority of clinically recognized pregnancy losses are early,[12]

sporadic in nature, and due to genetic abnormalities.[13] However, it has been recognized for decades that recurrent pregnancy loss (usually defined as three or more losses with no more than one live birth[6] is associated with antiphospholipid syndrome (APS). APS is an autoimmune disorder characterized by the presence of specified levels of antiphospholipid antibodies (aPL) and one or more clinical features, including pregnancy loss, thrombosis, or autoimmune thrombocytopenia.[14] The histologic findings of placental infarction, necrosis, and vascular thrombosis in some cases of pregnancy loss associated with antiphospholipid antibodies[15-16] have led to the hypothesis that thrombosis in the uteroplacental circulation may lead to placental infarction and ultimately, pregnancy loss. Numerous studies have linked recurrent first trimester pregnancy loss with APS[14,17-18] and aPL are detected in approximately 10 – 15% of these women.[14,17-18]

Recurrent early pregnancy loss has been associated with heritable thrombophilias as well. These disorders typically involve deficiencies or abnormalities in anticoagulant proteins or an increase in pro-coagulant proteins, and have been associated with the risk of vascular thrombosis and pregnancy loss. Several case series and retrospective studies reported an association between the Factor V Leiden mutation (associated with abnormal factor V resistance to the anticoagulant effects of protein C), the G20210A mutation in the promoter of the prothrombin gene, and deficiencies of the anticoagulant proteins antithrombin III, protein C, and protein S, and pregnancy loss.[19]

In most studies, thrombophilias were more strongly associated with losses after 10 weeks of gestation as opposed to anembryonic or embryonic losses. A recent meta-analysis indicated an odds ratio of 2.0 for “early” and 7.8 for “late” recurrent pregnancy loss for women with the factor V Leiden mutation, and an odds ratio of 2.6 for “early” recurrent fetal loss in those with the prothrombin gene mutation.[20] APS was shown to be more strongly associated with fetal death as opposed to anembryonic or embryonic losses.[21] These observations are not surprising given the high frequency and myriad causes (mostly genetic) of early losses.

c. Obstetric Complications

Pregnancies resulting in live births also may be complicated by abnormal uterine and placental blood flow. Examples include preeclampsia, small for gestational age fetus (SGA), and placental insufficiency resulting in fetal hypoxia or acidemia. Women with APS have a markedly high risk for preeclampsia and SGA.[22] These conditions also are associated with heritable thrombophilias,[23] although findings among studies are inconsistent.[23-24]

Regardless of thrombophilia status, numerous studies demonstrate an association between decreased uterine artery blood flow and disorders associated with placental insufficiency.[25-27] A meta-analysis of 27 studies concluded that abnormal uterine artery Doppler studies increase the likelihood of developing preeclampsia six-fold, and the risk of SGA over three-fold.[28] It is noteworthy that results are inconsistent.[28] Also, histologic evidence of thrombosis and infarction are common features of preeclampsia and SGA,[29-30] suggesting a vascular etiology for these conditions.

Abnormal uterine blood flow may influence placental function in many ways. Decreased flow later in gestation leads to a reduction in oxygen and nutrients for the fetus with predictable adverse sequelae. Early in gestation, decreased perfusion may actually cause abnormal placental growth and development. Fisher and colleagues demonstrated that oxygen tension profoundly influences cytotrophoblast proliferation and invasion.[31] Inadequate maternal arterial blood flow may adversely affect the ability of cytotrophoblast to differentiate, inhibiting normal placental growth and development.[31] This may account for the shallow or absent invasion of uterine spiral arteries associated with preeclampsia.[32] Indeed, abnormal blood flow at the very beginning of pregnancy may harm placental development, leading to clinical consequences (e.g. the development of preeclampsia) that are not apparent until later in gestation.

Given the substantial impact abnormal vascular flow can have on ovulation, fertility, implantation, placental growth and development, miscarriage, fetal death, preeclampsia, fetal growth, and preterm birth, a medication that improves blood flow to reproductive organs and prevents placental thrombosis is extremely desirable. Ideally, the medication would be safe for mother and fetus both during conception and pregnancy, inexpensive, have few side-affects, and be widely available. Aspirin may be just such a medication.

B.2. Aspirin and Reproduction

Aspirin is best known among lay individuals for its analgesic properties. The drug has many other effects including anti-inflammatory and antipyretic actions. Aspirin also is a potent inhibitor of platelet aggregation, contributing to its anti-thrombotic effects. The primary biologic effects of aspirin are mediated by inhibition of the enzyme cyclo-oxygenase (Cox), also termed prostaglandin G/H synthase. There are at least two isoforms of Cox, Cox-1 and Cox-2, both of which are inhibited by aspirin.[33] These enzymes catalyze the conversion of arachidonic acid to eicosanoids, or lipid mediators. The end products of arachidonic metabolism are varied and influenced by local enzymes in specific cell types. Thromboxane (TXA_2) is the major Cox product in platelets and is prothrombotic since it promotes platelet aggregation and vasoconstriction. At high doses, aspirin inhibits prostacyclin formation (PGI_2) in endothelial cells. This effect also is prothrombotic since PGI_2 causes vasodilation and decreases platelet aggregation. However, at low doses (typically 70 – 150mg), aspirin effectively inhibits platelet production of TXA_2 with little effect on endothelial PGI_2.[34-35] This is primarily due to the first pass effect in the liver wherein circulating platelets are exposed to the aspirin but at low doses, the drug is metabolized before reaching vascular endothelium. Thus low dose aspirin (LDA), results in a net increase in the PGI_2:TXA_2 ratio, decreasing thrombosis and increasing blood flow.

The anti-inflammatory properties of aspirin also are desirable in the prevention and treatment of reproductive pathology. In part, the pathophysiology of some cases of infertility, implantation failure, idiopathic miscarriage, APS, and preeclampsia is immunologically mediated. Although success has been mixed, anti-inflammatory medications have been used to treat these conditions. Thus, aspirin may reduce the rate of obstetric complications by suppressing the immune response in addition to improving blood flow.

LDA is generally considered to be efficacious in the prevention of certain types of thrombosis such as ischemic events in patients with coronary artery disease.[36] The drug also may reduce the risk of thrombosis associated with essential thrombocythemia.[37-39]

LDA is extremely attractive as a therapy for reproductive disorders because of its' high safety profile. Randomized clinical trials in thousands of pregnant women showed no increase in adverse fetal sequelae in doses < 150 mg per day.[40-42] The safety of higher doses is less clear. Initial reports suggested that prolonged exposure to high doses of Cox inhibitors such as indomethacin or other non-steroidal anti-inflammatory drugs (NSAIDs) may cause adverse fetal effects. These include premature closure of the ductus arteriosus, pulmonary hypertension, renal damage, intraventricular hemmorhage, and necrotizing enterocolitis.[43-44] More recently, a meta-analysis concluded that indomethacin use was not associated with adverse fetal effects.[45] It is controversial as to whether aspirin use increases the risk for fetal malformations. Aspirin was not associated with an overall increase in the risk of congenital malformations in a meta-analysis of 22 studies.[46] The same meta-analysis reported a possible increase in the risk of gastroschisis (OR 2.37; 95% Confidence Interval (CI,) 1.44 – 3.88) in infants exposed to aspirin (325 mg per day) in the first trimester.[46] This is biologically plausible since this malformation may be caused by vascular disruption of mesenteric vessels.[47] Results were not confirmed in a recent population based case-control study[48] and the association between aspirin use and gastroschisis remains uncertain. One study indicated an increased risk of miscarriage in women taking aspirin or other NSAIDs during the prenatal period.[49] However, the study included few (53) women taking NSAIDs, did not include information regarding dose and duration of treatment, was subject to selection bias, and did not account for some confounding variables such as aspirin use in women undergoing assisted reproductive technology or those with APS. Indeed, aspirin is often used empirically to treat infertility, recurrent miscarriage and APS (see below). Studies regarding safety are difficult to compare due to different doses, duration, and timing (with regard to pregnancy) of aspirin use. Nonetheless, the majority of data indicates minimal fetal risk from *in utero* LDA exposure.

The discovery of two Cox isoforms led to hopes that selective inhibition of one isoform might allow for the beneficial effects of Cox inhibition, without the untoward effects. Cox-1 was thought to be constitutively expressed while Cox-2 was inducible in response to infection or inflammation, as well as normal labor.[50] The Utah group was one of the first to demonstrate this in gestational tissues in a murine model of infection-mediated fetal death.[51] It appeared as though inhibition of Cox-2 (expressed under abnormal conditions) provided the beneficial effects of NSAIDs while inhibition of Cox-1 (normally expressed) led to unwanted consequences, making it an attractive potential therapy for preterm labor.[50-51] Unfortunately, selective inhibition of Cox-2 may lead to the same untoward fetal effects observed after non-selective Cox inhibition.[52-53] Newer Cox-2 selective agents may yet prove to be effective and safe, but none are ready for general clinical use.[50]

B.3. Aspirin and Fertility

Interest in the potential therapeutic effects of LDA on human fecundity began with the demonstration of its beneficial use in women with APS. The aPL that characterize the syndrome have been linked to reproductive abnormalities such as recurrent pregnancy loss. Women with recurrent pregnancy loss in turn have been shown to develop a higher than expected incidence of unexplained infertility. Because abnormal implantation may be a common etiology for both pregnancy loss and unexplained infertility, aPL have been hypothesized to play a role in both conditions. This in part prompted the hypothesis that LDA may enhance fecundity. Aside from aPL, other potential pathologic mechanisms that may reduce fecundity but may be treated by LDA have been proposed including diminished uterine perfusion, decreased endometrial thickness, uterine/endometrial inflammation, abnormal folliculogenesis, diminished ovarian progesterone production, and luteinized unruptured follicle syndrome. Diminished uterine perfusion may be a root abnormality initiating each of the other hypothesized mechanisms. Each of these potential mechanisms is amenable to experimental verification, but studies to date have not proven efficacy.

a. Uterine Perfusion - It has been suggested that poor uterine perfusion is a proximate cause of infertility.[7] Indeed, LDA appears to improve uterine blood flow as measured by Doppler pulsatility index.[54-56] One postulated underlying therapeutic mechanism of LDA on uterine blood flow is the vasodilatory effect of an increased PGI_2:TXA_2 ratio.[55] Another possible benefit of LDA is the inhibition of platelet aggregation and thereby physiologic endometrial “hyperthrombosis”.

Paradoxically, the prostaglandin inhibitory effect of aspirin mediated by the irreversible acetylation of Cox can compromise implantation in women.[57-58] The net reproductive effect of aspirin appears to depend upon a balance of complex interactions between such opposing mechanisms as vasodilation and prostaglandin inhibition and possibly other dose-dependent effects of aspirin on prostaglandin economy. Although increased uterine perfusion may benefit implantation, it does not appear that the effects of aspirin are mediated by altered circulating sex steroid concentrations or by increased endometrial thickness as measured by transvaginal ultrasonography.[55,59-60]

b. Antiphospholipid Antibodies (aPL) and Infertility - Levels of aPL have been reported to be higher in women with infertility than in fertile controls.[61-62] It is unclear how these autoantibodies interfere with fecundity. One possibility is altered reproductive cell activity secondary to antibody binding to cell surface antigens (possibly containing phospholipids). Gametes, zygote cells and trophoblast could all be targets. Cell activity alterations also could disrupt hemostatic mechanisms leading to clinically significant microthrombi at the tissue level.[63] In turn, this may diminish implantation and subsequent fecundity.

A meta-analysis of seven studies including women with aPL undergoing in vitro fertilization (IVF), but not specifically treated for the presence of aPL, did not show an impact of the antibodies on implantation or pregnancy rates.[64] However, studies of the association of aPL and fecundity have included such wide variation of study

populations, antibodies tested, and treatment regimens, that conclusions regarding an association of aPL and fecundity, and the efficacy of treatment remain controversial.

The only randomized placebo-controlled crossover trial performed to date did not show a benefit to treatment in the small subset of aPL positive women with previously documented IVF implantation failure.[63] Women were treated with heparin and LDA precluding assessment of the independent effect of aspirin. Because LDA was not started until the day of embryo transfer, the effect of LDA and heparin on endometrial development could not be ascertained.

c. Aspirin Therapy and Outcome of In Vitro Fertilization (IVF) - Contributing factors to low baseline human fecundity have not been fully characterized but decreased implantation appears to be a major component. The protean effects of aspirin include both potentially helpful and harmful consequences upon the process of implantation. Therefore aspirin has been employed experimentally in attempts to enhance implantation. IVF offers an opportunity to analyze sequential steps in the early reproductive processes.

Available studies using aspirin during IVF have studied heterogeneous populations. These have included women whose endometriums were thin, previous poor responders to follicle stimulating hormone (FSH) of ovarian stimulation, donor oocyte recipients, and women whose oocytes are undergoing intracytoplasmic sperm injection (ICSI). Some studies included unselected populations (all infertility diagnoses and combinations) undergoing IVF. Also, aspirin protocols in published IVF studies vary as to dose, duration and cycle starting point. Not surprisingly, the conclusions vary regarding the efficacy of aspirin for an infertile IVF population and mechanistic effects cannot be precisely defined.

A total of 2,500 IVF patients have been randomized to aspirin versus placebo or no treatment in seven trials. The largest study (N = 1380) used unselected IVF patients and reported a statistically significant benefit for the use of LDA.[65] The possibility remains that routine use of aspirin for IVF (as is currently being done in many reproductive centers) may confer benefit. Fortunately, no serious side effects of aspirin use for IVF have been reported.

B.4. Aspirin and Pregnancy Loss

LDA was first used to treat recurrent pregnancy loss in women with APS. Use of the drug was prompted by recognition that thrombosis was central to the pathophysiology of APS, as well as the observation that IgG fraction from women with APS increases placental production of TXA_2 in humans.[66] Aspirin reduced TXA_2 under similar conditions without affecting PGI_2, thus decreasing the TXA_2:PGI_2 ratio.[67] It is somewhat difficult to assess the independent effect of aspirin in women with APS since most have been treated with additional agents such as heparins and or prednisone. Systematic reviews[68-69] of interventions intended to improve obstetric outcome in women with aPL identified three trials comparing aspirin alone to no treatment.[70-72] Aspirin alone had a relative risk of 1.05 (95% CI, 0.66, 1.68) for live birth compared to no treatment.[68-69] It is noteworthy that these trials were very small, included many women who do not meet

criteria for APS (either low titers of antibody or too few losses or medical problems), and were not all placebo controlled.[70-72] The authors conclude that a small benefit of aspirin alone cannot be rejected on the basis of available studies.[68] Unfractionated heparin combined with aspirin showed a relative risk of 0.46 (95% CI, 0.29 – 0.71) for pregnancy loss in two trials including 140 women with recurrent pregnancy loss and APS compared to aspirin alone.[68-69] A third trial showed no difference between low molecular weight heparin and aspirin compared to aspirin alone.[73] Both groups in the latter trial had excellent outcomes.

LDA also has been used to treat recurrent pregnancy loss in women without aPL. Most trials used heparin as well as aspirin, making it difficult to determine the independent effect of aspirin. A systematic review of anticoagulants for the treatment of women with recurrent pregnancy loss (defined as two early losses or one unexplained fetal death) identified two controlled trials.[74] One included 54 women and compared LDA to placebo where LDA had a relative risk for live birth of 1.00 (95% CI, 0.78 – 1.56).[72] The other included women with heritable thrombophilias and fetal death.[75] Although heparin improved the odds of live birth in this cohort compared to women treated with LDA,[75] the presence of a heritable thrombophilia may introduce a substantial bias in the findings.

The gestational age of prior losses may influence whether or not LDA is effective in improving subsequent pregnancy outcome. Rai and colleagues assessed the effect of LDA on pregnancy outcome in women with recurrent pregnancy loss.[76] The study was retrospective and not randomized, although the group was large (805 women with early miscarriage and 250 with late losses). LDA did not have a statistically significant increase in the live birth rate in women with early losses (OR 1.24, 95% CI, 0.93-1.67). In contrast, in women with unexplained fetal death, the OR for live birth associated with LDA was 1.88 (95% CI, 1.04-3.37).[76] The Utah group had similar findings in an uncontrolled retrospective cohort of 230 women with unexplained fetal death.[77] In univariate analysis, LDA was associated with an OR of 0.41 (95%CI 0.25-0.68) for subsequent pregnancy loss. After controlling for confounders in a multivariate analysis, LDA was associated with an OR of 0.12 (95% CI 0.05-0.32) in women 35 years of age or older.[77] Thus, LDA appears to be more helpful in women with prior fetal death as opposed to recurrent first trimester losses. We speculate that LDA may improve the utero-placental circulation, decreasing the risk of placental thrombosis, infarction, and insufficiency which have been associated with fetal death.[77-78] On the other hand, anembryonic and embryonic losses are common in normal individuals and often are due to *de novo* non-dysjunctional aneuploidy. Accordingly, aspirin may be less likely to help patients with recurrent early pregnancy loss. There is still a potential role for LDA in the treatment of some cases of early pregnancy failure. Aspirin (as well as heparin) modulates trophoblast apoptosis in BeWo cells and placental explants in vitro, providing another mechanism for aspirin to improve pregnancy outcome.[79]

B.5. Aspirin and Obstetric Complications

The best studied use of LDA in reproduction is the prevention of preeclampsia. As with APS, there are several reports indicating an increase in the TXA_2:PGI_2 ratio (metabolites) in serum in women with preeclampsia compared to normotensive controls.[80-81] Vainio and colleagues demonstrated an improvement in this ratio in women

treated with LDA.[81] Recent data from a murine model also support this paradigm.[82] Depression of platelet TXA_2 while preserving reproductive function (intended to mimic the effects of LDA) decreased platelet aggregation and prevented thrombosis.

Many large randomized controlled trials have been conducted in populations with both high and low risk for the development of preeclampsia. Duley et al performed a systematic review that included 51 trials involving 36,500 women treated with antiplatelet agents for the prevention of preeclampsia.[83] Forty-four of the trials involved the use of aspirin alone (compared with placebo or no treatment) while the remainder included other treatments, often in conjunction with aspirin. Overall, use of antiplatelet agents conferred a 19% reduction in the risk of preeclampsia (RR 0.81; 95% CI, 0.74-0.96). LDA was associated with a risk reduction for the development of preeclampsia associated with proteinuria, regardless of whether women were at moderate or high risk for preeclampsia, gestational age at trial entry, or dose of LDA.[83] There was a greater risk reduction in women treated with doses greater than 75 mg/day (RR 0.49, 95% CI 0.38-0.65) compared to lower doses (RR 0.86, 95% CI 0.79-0.93). Others reviews emphasize the increased benefit from LDA in women with historical risk factors (high risk for preeclampsia) and suggest that focusing on at risk groups would decrease the number of women it is necessary to treat to prevent a single case of preeclampsia.[84-86] Of course, the “risk:benefit” ratio also is influenced by risk, which appears to be quite low for LDA. Thus, treatment of low risk women may yet prove to be justified.

LDA also may reduce the risk of other adverse perinatal outcomes such as SGA fetus and late fetal death. In 32 trials of 24,310 women, antiplatelet therapy conferred an 8% reduction in SGA (RR 0.92, 95% CI 0.85-1.00) in women treated with the intent to prevent preeclampsia.[83] A small trial showed no benefit from LDA in women treated after the diagnosis of SGA fetus.[87] LDA also has not been effective when started after the diagnosis of preeclampsia.[88-89] The review by Duley and colleagues noted a 16% reduction in combined fetal, neonatal, and infant mortality in women taking antiplatelet therapy (RR 0.84, 95% CI 0.74-0.96). Perinatal death and SGA fetuses were not primary end points of these trials and results should be interpreted with caution.

Doppler velocimetry of the uterine, umbilical, and fetal arteries have been used to estimate flow and vascular resistance during pregnancy.[90-91] Although the positive predictive value is poor, especially in low risk populations, there is a recognized increase in subsequent risk of preeclampsia, SGA, and fetal death in women with abnormal uterine artery Doppler studies in the second trimester.[28] Several investigators have treated women with abnormal uterine artery Doppler studies with LDA in attempt to improve perinatal outcome. In women at high risk for the development of preeclampsia and SGA with abnormal uterine artery Doppler studies, LDA can reduce the rate of subsequent preeclampsia.[92-94] In contrast, LDA did not improve outcome in most investigations conducted in low risk populations with abnormal Doppler studies.[95-97]

Prevention of preterm birth is a very important potential benefit of LDA since prematurity is a major cause of perinatal morbidity and mortality. The drug may decrease the risk of preterm birth indirectly by reducing the risk of preeclampsia, SGA, and placental insufficiency. These conditions often result in iatrogenic preterm birth in an attempt to improve perinatal outcome. LDA also may have a direct effect on preterm labor and

delivery since Cox inhibition decreases uterine contractility and high dose aspirin use has been associated with delayed onset of labor. In the systematic review by Duley et al., data were available regarding preterm birth from 28 studies including over 30,000 women. The authors noted a 7% reduction in delivery < 37 weeks gestation in women treated with antiplatelet agents (RR 0.93, 95% CI 0.89-0.98). These findings are intriguing because even a small reduction in the rate of preterm birth would have dramatic effects on perinatal morbidity and health care costs.

B.6. Remaining Questions

Available data suggest that LDA has the potential to favorably impact on several aspects of reproduction including conception, implantation, early pregnancy loss, late fetal death, preeclampsia, SGA fetus, placental insufficiency and preterm birth. LDA is an ideal therapy; it is apparently safe, widely available, has few maternal side effects, and is inexpensive. The possibility that such a drug could have a major impact on obstetric outcome is extremely attractive. It is unclear which patients would benefit from LDA. Prior studies have focused on narrow aspects of the effect of LDA such as IVF outcome or the development of preeclampsia. Reproduction is unique in that the different stages are inextricably linked. Better blood flow at the time of conception and implantation may ultimately lead to improved placental function, and a reduced risk of preeclampsia, SGA, and preterm birth. Unanswered questions about LDA and pregnancy can only be answered through a comprehensive, carefully designed, prospective randomized clinical trial, such as that proposed in the EAGeR trial.

C. STUDY DESIGN

C.1. Introduction

The EAGeR study is a two-site prospective randomized double-blind placebo-controlled clinical trial evaluating the effect of low-dose aspirin use on gestation and reproduction outcomes. The target population is women aged 18-40 who have had one or two pregnancy losses in the past and who are actively trying to conceive. Women shall be screened at enrollment to ensure they are not pregnant and eligible with the goal of recruiting and randomizing 1600 completed subjects into two clinical sites. Subjects shall be randomly assigned to receive low dose aspirin (81 mg) plus folic acid (400 mcg) or identical looking placebo plus folic acid. These enrollees will be actively followed for two menstrual cycles or until becoming pregnant, whichever comes first. Active follow-up entails collection of questionnaire data as well as regular home specimen collection. Women not becoming pregnant within the first two cycles shall remain in the study under passive follow-up for an additional four menstrual cycles or until becoming pregnant, whichever comes first. Passive follow-up entails intake of Study Pills and folic acid and use of fertility monitors, pregnancy testing, and follow up questionnaires, but no home specimen collection. Women who become pregnant shall be followed throughout gestation for pregnancy outcomes and will continue to take Study Pills and folic acid throughout pregnancy until their 36th gestational week.

The trial will be conducted at two clinical sites, the University of Utah in Salt Lake City, Utah and the University at Buffalo in Buffalo, New York. The Utah site includes four medical centers and the Buffalo site will be conducted at a free standing research clinic at the University enrolling participants from the western NY region. Henceforth the term "clinical center" refers to each of the five clinics participating in the trial at the two sites. The study will take a total of 5 years to complete including start up activities (Phase I), recruitment and follow-up of pregnancy outcomes in all subjects (Phase II) and wrap-up and analysis (Phase III). Participants will be recruited from throughout the community and enrolled over approximately a three to four year time period. By the end of the study, it is expected the University of Utah will have enrolled 1100 complete subjects, two thirds of the study population, and the University at Buffalo will have enrolled 500 complete subjects. **To be considered a complete subject, participants need to complete at least 90% of their scheduled clinic visits.** The number of required visits will depend on the participant's length of follow-up in the trial.

C.2. Eligibility Criteria

The target population is women of childbearing age who experienced one or two spontaneous pregnancy losses in the past and are actively trying to get pregnant. Women must meet the following eligibility criteria to be considered for participation in the EAGeR trial:

Inclusion Criteria:

a Women experiencing one or two pregnancy losses at any point in gestation in the past that were not elective termination(s). At least one of these losses must be well documented by one of the following:

- Sonogram demonstrating anembryonic loss, embryonic loss or fetal death.
- Histologic confirmation of products of conception that were spontaneously passed per vagina or surgically obtained.
- Hospital records of fetus delivery
- Late menses and positive serum hCG or positive urine hCG documented by hospital or clinic records followed by either a negative hospital/clinic pregnancy test or a decline in urinary hCG level over 3 days.*

b No more than 5 pregnancies in total including the pregnancy loss(es)**.

c Up to two prior pregnancies that did not end in a loss **

d Presence of intact tubes (both), ovaries (both), and uterus.

e Between 18 and 40 years of age at time of baseline visit

f Regular menstrual periods between 21 – 42 days in length (within the last 12 months). Regular menstrual periods are defined as no more than an 8-day difference between the woman's shortest and longest cycle.

g No more than one missed menses in the past 12 months (other than those missed due to pregnancy or breastfeeding).

h Actively trying to conceive with a male partner and not using contraception by the baseline visit.

i Not currently pregnant at the baseline or randomization visits.

j Ability and willingness to give informed consent.

k Willingness to be randomized and to take daily study pills for 6 months to a possible 15 months.

* Home pregnancy tests without hCG confirmation from a healthcare provider (either serum or urine) will not be accepted.

** Women may have up to two pregnancies beyond 20 weeks that were not losses, two spontaneous pregnancy losses at any time in the past, and up to one therapeutic or elective termination (two therapeutic or elective terminations if no other pregnancies). Ectopic and molar pregnancies would, for the purpose of enrollment, be considered in the same category as therapeutic termination pregnancies. Women with more than two live births or those with more than two losses, regardless of the week of gestation of the loss, are excluded.

Exclusion Criteria:

a Known allergies to aspirin or non-steroidal anti-inflammatory agents.

b Clinical indication for anticoagulant therapy. These include prior or current thrombosis, antiphospholipid syndrome (APS) or known major thrombophilia.

c Clinical indication for chronic use of NSAIDs such as rheumatoid arthritis.

d Indication for additional folic acid supplementation, such as prior infant with neural tube defect (NTD), seizure disorder.

e Medical contraindication to aspirin therapy. These include uncontrolled asthma, nasal polyps, bleeding disorders, or history of gastrointestinal ulcer.

f Presence of major medical disorders (regardless of severity). These include diabetes, hypertension, systemic lupus erythematosus (SLE), untreated or active cancer (any cancer in remission or non-melanoma skin cancer is not included in the exclusion criteria), liver disease, renal disease, rheumatoid arthritis, cardiac disease, pulmonary disease other than mild asthma, neurologic disease requiring medical treatment, uncontrolled hypothyroidism, uncontrolled seizure disorder. Untreated vitamin B_{12} deficiency, severe anemia (Hct < 30%), hemophilia, gout, nasal polyps, among others.

g Currently undergoing/planned use of assisted reproductive techniques during trial (IVF; IUI; Clomid)

h History of infertility or sub-fertility. This includes any of the following:

- No conception after ≥ 1 year of unprotected intercourse and actively trying to conceive.
- Any prior medical treatment for infertility.
- Prior treatment for known pelvic inflammatory disease.
- Known male infertility or sperm abnormality (current partner)
- Known tubal occlusion, anovulation, uterine abnormality, or endometriosis stage III or IV.
- History of polycystic ovarian syndrome.

i Presence of unstable mental disorder. These include bipolar illness, schizophrenia, uncontrolled depression, uncontrolled anxiety disorder.

j Known current or recent alcohol abuse or illicit drug use.

k Current diagnosis of sexually transmitted infection (STI) (temporary exclusion)

C.3. Treatment groups

The trial aims to evaluate the effects of low-dose aspirin on gestation and reproductive outcomes. In addition to the LDA, all participants will be provided a folic acid supplement to be taken daily, which is currently routinely recommended for women attempting to become pregnant.

Participants will be randomized into two groups receiving one of the following:

- Aspirin 81 mg taken orally daily plus folic acid 400 μg taken orally daily
- Placebo plus folic acid 400 μg taken orally daily

Aspirin and placebo capsules will be identical in look and weight to maintain blinding. The duration of treatment regimens will be up to 6 menstrual cycles, with those who become pregnant continuing until week 36 of pregnancy.

For the remainder of the protocol, the term “Study Pills” will be used to refer to aspirin and placebo to differentiate it from folic acid.

C.4. Outcome Measures

a hCG recognized pregnancy (implantation)

Home pregnancy kits (Quidel Quickview) will be used to identify pregnancies at the time of implantation. A positive qualitative result on a home pregnancy test followed by confirmation of quantitative hCG levels ≥ 0.025 ng/mL in urine specimens will define hCG pregnancy. Positive spot urine test at clinic visits will be also considered as hCG pregnancy. Quantitative hCG analysis will be performed from batched urine samples sent to the NIH repository.

b Clinically recognized pregnancy

Clinical recognition of pregnancy will be determined by

1) Documentation of the gestational sac from an ultrasound scan(s) at 6.5 weeks or later (or fetal heart tones with histologic confirmation of gestational tissue). We will utilize physicians’ reports from routinely scheduled prenatal care as well as the study ultrasound performed early in pregnancy.

OR

2) Positive serum hCG test and missed menses

c Early pregnancy loss (EPL)

Pregnancy loss is a measure of impaired human fecundity and encompasses two types of losses: 1) early pregnancy loss or hCG detected pregnancy loss and 2) clinically recognized pregnancy loss. It is estimated that approximately one-third of all pregnancies are spontaneously aborted of which 2/3 are hCG pregnancy losses.

- hCG pregnancy loss

  An hCG pregnancy loss will be defined as a urinary hCG level ≥ 0.025 ng/mL for at least 3 consecutive days followed by a decline. The rise and fall of hCG will be evaluated during the 10 days prior to the next menstrual period through day 5 of the next menstrual period. Quantitative hCG analysis will be performed from batched urine samples sent to the NIH repository.

- Clinically recognized spontaneous abortion

A clinically recognized spontaneous abortion will be defined as that detected by the woman or her doctor before 20 weeks completed gestation and having had a confirmed pregnancy by documentation of the gestational sac or heart tones (see above).

d Pregnancy losses occurring less than 10 weeks may be anembryonic, embryonic, or unknown

- Anembryonic pregnancy loss

  An anembryonic pregnancy is a gestation in which embryonic development does not occur. Sonographic criteria include:

  1. Mean gestational sac diameter of ≥ 16 mm without an embryo
  2. Mean gestational sac diameter of ≥ 8 mm without a yolk sac
  3. No visible embryo two weeks after detection of gestational sac of any size.
  4. Positive serum hCG with missed menses and no sac visible on sonogram.

- Embryonic pregnancy loss

  Pregnancy loss occurring before 9 weeks completed gestation. Sonographic criteria include:

  1. Embryo with crown rump length ≤ 30 mm with no cardiac activity.

e Fetal pregnancy loss

Pregnancy loss occurring at 10 to 19 completed weeks gestation. Death of a fetus with crown rump length of > 30 mm and composite mean gestational age (based on biparietal diameter, abdominal circumference and femur length) < 20 weeks completed gestation.

f Stillbirth

Pregnancy loss occurring AT OR after 20 completed weeks gestation. The complete expulsion or extraction from its mother, after at least 20 completed weeks pregnancy, of a product of conception in which, after such expulsion or extraction, there is no breathing, beating of the heart, pulsation of the umbilical cord, or unmistakable movement of voluntary muscle.

h. Ectopic Pregnancy

A pregnancy that has implanted somewhere other than the uterus as confirmed by sonography, laparoscopy or laparotomy.

i. Molar Pregnancy (determined by pathology)

1. Complete hydatidiform mole: Hydropic degeneration and swelling of villous stroma, absence of blood vessels in the swollen villi, proliferation of trophoblastic endothelium, and absence of a fetus and amnion.
2. Partial hydatidiform mole: Hydropic degeneration and swelling of some (usually avascular) villi, with sparing of other villi. Focal trophoblastic hyperplasia. The fetus usually has features of triploidy and the karyotype is usually triploid.

j. Live birth

Birth of a fetus with any sign of life (e.g. pulsation of umbilical cord, purposeful movements)

k. Preeclampsia

The following definitions for preeclampsia will be used as recorded in participants' medical charts during pregnancy and obtained with chart abstraction.

Mild pregnancy related hypertension is defined as a systolic blood pressure ≥ 140 or diastolic blood pressure ≥90 on two occasions 4-240 hours (10 days) apart on or after 20 weeks 0 days of gestation. The first of the two blood pressures must have been obtained prior to 24 hours postpartum. All blood pressures obtained during hospital admissions will be counted, except intra-operative blood pressures and systolic blood pressures during labor.

Normotensive: A woman is considered normotensive if she was not previously diagnosed as being hypertensive. A women is considered to be previously hypertensive if she reports a physician documented history of elevated blood pressure outside of pregnancy or she manifests elevated blood pressure (systolic >140 mmHg or diastolic >90 mmHg, documented by physician) any time between conception and 140 days of gestation.

Note that the screening for EAGeR trial excludes women with known history of hypertension and those treated with anti-hypertensive drugs.

Proteinuria is defined by any of the following criteria
1) A total protein excretion value of ≥ 300 mg in a 24-hour urine
2) The finding of ≥ 2+ on dipstick or a protein creatinine ratio ≥ 0.35 if a 24-hour urine is not available.
3) If a 24-hour urine is not available, the finding of two or more urine dip sticks value of ≥ 1+ in the absence of a urinary tract infection (defined by a positive culture or nitrite dipstick that was regarded by the managing clinician as representing infection). The two values must be obtained from 4-240 hours of each other.

The 24-hour urine is the definitive test for proteinuria and supersedes all previous dipstick values. Thus if two dipstick readings show ≥ 2+ but the follow-up 24-hour urine collections shows protein < 300 mg, the patient is not considered to have proteinuria.

Mild preeclampsia is defined as mild pregnancy related hypertension and proteinuria, as defined above, if at least one elevated blood pressure measurements (systolic >140 mmHg or diastolic >90 mmHg) is recorded within 72 hours of diagnosis of proteinuria.

Severe preeclampia

Several criteria are used:

1) Severe pregnancy related hypertension plus proteinuria. Severe pregnancy-related is defined as a systolic blood pressure ≥ 160 or diastolic blood pressure ≥110 on two occasions 4-240 hours (10 days) apart on or after 20 weeks 0 days of gestation in a previously normotensive woman. The first of the two blood pressures must have been obtained prior to 24 hours postpartum. All blood pressures obtained during hospital admissions will be counted, except intra-operative blood pressures and systolic blood pressures during labor. A single systolic blood pressure ≥ 160 mmHg or diastolic blood pressure ≥ 110 mmHg qualifies for this diagnosis if the participant was treated with anti-hypertensive medication.
2) Mild preeclampia with severe proteinuria (24-hour urine collection ≥ 5 grams protein)
3) Mild pregnancy related hypertension and oliguria (24-hour urine collection < 500 ml volume)
4) Mild pregnancy related hypertension and pulmonary edema (confirmed by chest x-ray)
5) Mild pregnancy related hypertension and thrombocytopenia (a platelet count < 100,000/mm3)

HELLP Syndrome

Pregnancy related hypertension (mild or severe) with all of the following occurring within 72 hours of the elevated blood pressure measurement:
a. Thrombocytopenia: platelet count < 100,000/mm^3
b. SGOT (AST) ≥ 100 U/L
c. Hemolysis: either LDH ≥ 600 U/L or total bilirubin ≥ 1.2 mg/dl or peripheral blood smear showing nucleated RBCs, schistocytes, or an elevated reticulocyte count

Eclampsia

Pregnancy related hypertension (mild or severe) and a seizure during pregnancy

Hierarchy of conditions severity

If a participant satisfies the criteria for more than one of the categories (for example, if she satisfies the criteria for mild preeclampsia but also has a platelet

count less than 100,000/mm3) she will be diagnosed in the highest numbered category (arbitrarily considering severity to be in the order – mild preeclampsia, severe preeclampsia, HELLP, eclampsia).

l. Small for gestational age infant

Infant birthweight is ≤ 10% for gestational age (weight < 10th percentile), as defined by the standards of Kramer, et al .

(**http://www.pediatrics.org/cgi/content/full/108/2/e35**).

m. Preterm birth

Delivery prior to 37 weeks of completed gestation. Spontaneous preterm labor and delivery and preterm premature rupture of membranes will be distinguished from indicated preterm birth due to for example preeclampsia or SGA fetus.

n. Abnormal fetal testing

Non-reactive non-stress test (NST), positive contraction stress test (CST), abnormal biophysical profile (BBP ≤ 6).

o. Fetal intolerance of labor

Cesarean delivery due to abnormal fetal heart rate tracing.

p. Abruption or vaginal bleeding

Abruption or vaginal bleeding that results in hospitalization, Cesarean delivery, or pre-term birth.

q. Length of hospital stay for the infant.

r. Cost: Cost data collected will include charges (rather than actual cost) for maternal and neonatal inpatient care. Data will be collected from the hospital of delivery for live births. Data regarding charges will be obtained through hospital administrative electronic records. Additional data regarding this endpoint will include total length of stay (maternal and neonatal) as well as ICU admission. These data will be obtained from the medical record.

For the above outcomes, gestational age will be determined by the following:

1) Gestational age will be based on the 6.5 week study sonogram finding.
2) For participants with a pregnancy loss before the 6.5 week ultrasound, gestational age will be defined as the number of days from ovulation to the loss and then add 14 days onto that.
3) If the 6.5 week study ultrasound is not available and the pregnancy loss is believed to be after the point when the 6.5 week ultrasound should have been done, use the earliest sonogram obtained clinically in pregnancy and compare it to the last menstrual period (LMP). If within the following limits, use the LMP as the gestational age. If not, then use the sonogram results.

Gestational age according to first ultrasound	Limit of agreement to accept LMP
Up to 19 weeks, 6 days	±7 days
20 weeks, 0 days to 29 weeks, 6 days	±14 days
30 weeks, 0 days or greater	±21 days

If there are no available sonograms but the date of ovulation is available, then gestational age will be defined as the days from ovulation to delivery, plus 14.

If there are no available sonograms and date of ovulation is not available, the LMP will be used for the gestational age (ie delivery date-lmp date)

D. PARTICIPANT MANAGEMENT

D.1. Recruitment

The recruitment plan for the EAGeR trial will be site-specific.

Recruitment plan summary

Potential study participants will be recruited from multiple sources so as to reach a diverse study population that includes women who do and do not routinely access the health care system. Two main recruitment methods will be employed to reach potential subjects: **clinic-based recruitment** and **community-based recruitment**. Recruitment methods will include attempts to enroll women of various racial and socioeconomic backgrounds.

The main recruitment strategy of this trial will be based on the clinical settings, where women who have recently had a pregnancy loss are readily identified and approached. These settings will include hospital-based emergency departments, hospital-based ultrasound units, and private obstetrician/gynecology practices. Clinical sites will be sent a letter describing the study objectives, the goals of the trial and the need for subjects, and will be given study brochures and/or study contact forms for use in their office. The brochures will include a description of the study, some basic Q&A and a telephone number with information on how to reach study personnel. Women can either contact study coordinators directly or give consent for study coordinators to contact them.

Community-based recruiting will be done through a wide variety of mechanisms, including disseminating information about the study through advertisement in the media, direct mail, placement of flyers in community centers, and appropriate website posts. Because of the success of tear-off posters, flyers and word of mouth in recruiting for previous studies, these methods will be employed with EAGeR as well.

D.2. Study Visits and Contacts Overview

The planned follow-up schedule for the EAGeR trial includes an initial telephone screen for general eligibility, an in-clinic screening visit including baseline assessments and measures, and then an enrollment/randomization visit (day 2-4 of menstrual cycle), followed by two menstrual cycles of active follow-up (or until becoming pregnant, whichever comes first), followed by four months of passive follow-up (or becoming pregnant, whichever comes first). Active follow-up entails collection of questionnaire data and frequent specimen collection. Women not becoming pregnant within the first two cycles will remain in the study under passive follow-up for four additional cycles (or until becoming pregnant, whichever comes first). Those not pregnant by the end of passive follow-up will complete study participation. Women who become pregnant in the six months of study follow-up will be followed throughout gestation for pregnancy outcomes. During the one month following conception, the pregnant women will undergo close monitoring with specimen collection and clinical evaluations. Details of study visits are as described below.

To be considered a completer, a study participant must complete at least 90% of her scheduled clinic study visits (number of scheduled visits will vary depending on the length of follow up).

The outline of participants' follow-up in the EAGeR trial is depicted in Figure 1.

Figure 1. Participants' management in the EAGeR Trial

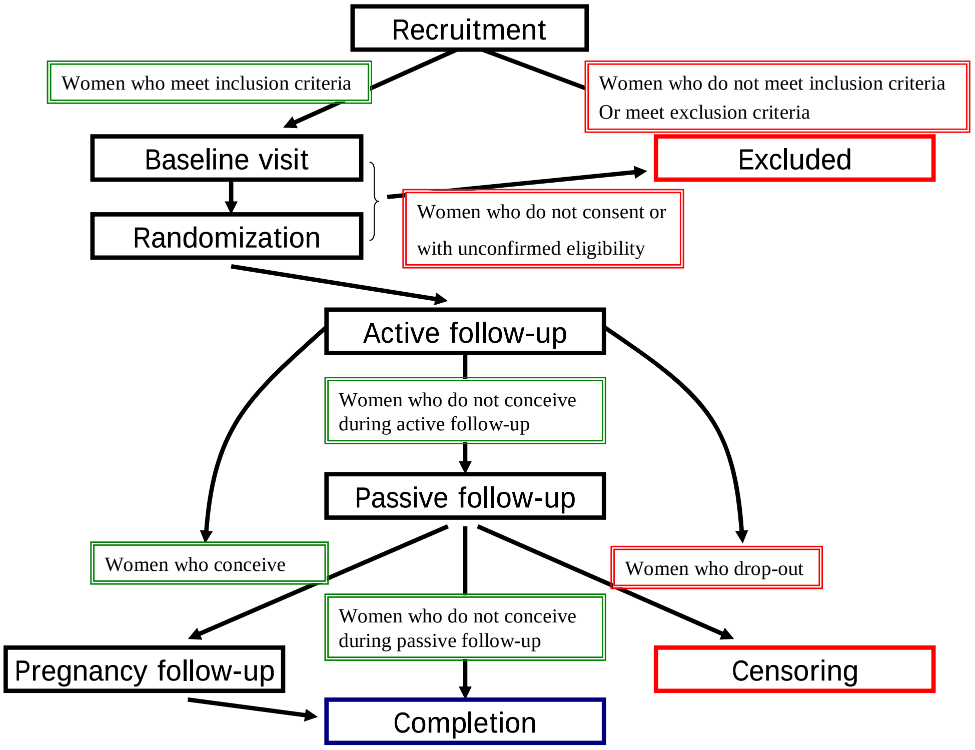

D.3. Screening process

Initial screening may take place in person or by telephone. Women who meet initial screening criteria will be invited to participate in the study and scheduled for a baseline visit in the study clinic at the earliest convenient date. Study personnel will obtain informed consent at the baseline visit for all participants.

Telephone Screen:
Preliminary contacts with interested women can be made directly with study personnel by phone or by email/web inquiries. Regardless of how initial contact is made, each potential participant will be contacted by telephone by one of our staff.

When the initial call comes into the clinic, the potential participant will be greeted warmly and the staff will ask a few introductory questions including asking their name, address, telephone number(s), how they heard about the study and their current age. The staff will then provide a basic overview of the study including the objectives and the basic

study requirements. They will be given an opportunity to ask questions. After the initial questions have been answered, the caller will be told that there are some initial screening questions that we will need to be asked to determine their basic eligibility. They will be asked to answer a series of eligibility questions (see Appendix 1). The telephone screen will be completed in its entirety, regardless of their answers to any individual question. The complete list of general eligibility questions (entire telephone screen) will be asked before a determination is made regarding eligibility in order to better characterize the prospective participants (all the reasons they are ineligible). Questions on the source of referral and how they heard about the EAGeR trial will be included on the screening questionnaire for recruitment tracking purposes.

If the woman is found to be *ineligible*, she will be informed that there are certain criteria that are predefined for study inclusion and that she does not meet these eligibility criteria. They will be thanked for their time and interest and the call will be ended. If they are determined to be *eligible* based on initial screening criteria, we will inform them that they do in fact meet our initial eligibility criteria. They will then be provided with additional details about the study and asked to schedule a baseline appointment at their convenience. They will be asked to provide additional contact information. A screening form script has been developed to ensure standardization of the screening process and to ensure information given to study participants is consistent (see Appendix 1).

If a baseline appointment is made after the telephone screening, the informed consent form will be mailed to the participant to read over before the baseline appointment. The consent form will be discussed in detail and signed at the baseline visit.

D.4. Baseline study visit (1st clinic visit, V-BL)

The baseline visit will entail informed consent, detailed eligibility, and data collection including administered questionnaires, physical measurements and laboratory testing (urine samples).

At this first baseline clinic visit, each potential participant will meet with the study coordinator or research assistant. At this visit, study objectives, participation requirements, and the consent form will be described in detail. After signed consent is obtained, a detailed eligibility questionnaire will be administered to ensure that the woman fits the inclusion criteria and has no conditions that could fit the exclusion criteria. Next, a urine specimen will be collected and a urine pregnancy test will be performed to confirm the woman is not pregnant (if pregnant, she will be excluded from the study). Study ID number will be generated at this visit.

Baseline questionnaires will be interviewer-administered and entered on computerized Web-forms.

The following questionnaires will be administered to elicit information on socio-demographic characteristics, health-related behavior and medical history (See Appendix 2 for the baseline questionnaires). Reported menstrual and reproductive history will be recorded in detail.

- Demographic Questionnaire

- Health and Reproductive History Questionnaire Part B
- Exercise Questionnaire
- Lifestyle Questionnaire
- Occupation Questionnaire

Participants will be given the Personal Information Questionnaire, Family Medical History Questionnaire and Part A of the Health and Reproductive History Questionnaire to be completed at home. Participants will bring the completed forms to the next clinic visit (the randomization visit) where study staff will review the forms for completeness and answer any questions. These questionnaires are sent home with the participant to reduce the amount of time spent at the research clinic and since the participants may need to use home records or other contacts in order to complete the questionnaires.

In addition to these questionnaires, blood pressure and anthropometric measurements (height, weight, waist to hip ratio, skin fold measurements) will be taken (See Appendix 2 for the Physical Measurements Form). Participants will also be asked to insert a vaginal swab to provide a Gram stain sample.

Participants will also receive brief instructions for the use of fertility monitors and specimen collection kits. These directions will be reinforced in more detail at the randomization visit.

At the end of the baseline visit, those subjects that remain interested and eligible will be scheduled for an active study visit where final enrollment and randomization will take place. Based on the menstrual history and the expected date of the start of the next menstrual cycle, the next appointment will be made to coincide with day 3 (2-4) of the next menstrual cycle. Delay of randomization until this visit allows additional opportunity to assess the commitment of the participants and to minimize dropout during the trial.

In addition to the baseline questionnaires, participants will be asked to provide authorization (i.e. release) to send a brief questionnaire to their gynecologist to secure details regarding the reported spontaneous abortion (SA) including date and results of the serum hCG, date and gestational age at the time of the SA, ultrasound findings (if completed), histology, and course following SA (i.e. D&C). (See Appendix 3 for the event verification form).

D.5. Randomization

D.5a. Randomization Visit (V-RD)

Following the baseline visit, participants will return for the final enrollment and randomization visit. The randomization visit constitutes the first active follow-up visit and its content aside from the randomization is detailed in the next section, ***D.6a.***

Briefly, during this visit, participants will be randomized following the protocol described below, receive their assigned treatment (3 month supply), and receive the testing materials for the study, i.e., fertility monitors, specimen collection kits, and daily diaries. The study staff will review the instructions with the participant again. Randomization will

be in the ratio of 1:1, treatment to placebo stratified by eligibility group and clinical center. Participants will be counseled to begin taking treatment daily starting that day. The next active follow-up visit will be scheduled for approximately the mid point of the current menstrual cycle.

Pill Dispensation: Study Pills and folic acid will be provided in bottles with a three month supply. Each Study Pill bottle will have enough pills to amply cover cycles 35 days long (105 capsules). Folic acid will be supplied in bottles containing 100 tablets (due to how the folic acid is commercially available). Bottle labeling and bar code verification will be established as described under "Randomization protocol section D5b below. Bottles, both unopened and opened, will be brought in for weighing at each clinic visit throughout the study to evaluate adherence.

At the randomization visit, participants will be given fertility monitors to help them predict their fertile period and time intercourse to maximize the likelihood of becoming pregnant. The fertility monitor will also provide information on the participant hormone levels and cycle for research purposes. The fertility monitor translates urine dipstick hormone measurements into predictions for the fertile period and is suitable for women whose menstrual cycle length falls within 21-42 days regardless of regularity. The monitor tracks the urinary metabolite of estradiol – estrone-3-glucuronide (E3G) and luteinizing hormone (LH) determined from dipsticks and displays the corresponding results (i.e., low, high or peak fertility days). The peak fertility days will also be used to schedule subsequent appointments during active follow-up.

D.5b. Randomization Protocol

Randomization of subjects will be carried out to obtain the 1:1 allocation ratio between the treatment and placebo arms. Randomization will be stratified by center and by eligibility criteria group. Two eligibility groups will be considered: group I including all women who have had only one spontaneous abortion in gestation week <20 and within the last 12 months, no more than one live birth in the past and no pregnancy losses occurring on or beyond 20 completed weeks of gestation; group II includes women who do not fit group I but are eligible by the modified criteria. A computer algorithm will create the random assignment to one of the treatment arms based on random permuted blocks design with randomly varied block size.

The random sequence of treatment assignment will be linked to a pre-determined list of study ID numbers generated at the Haifa DCC for each clinical center and to a corresponding non-informative list of medication bottle numbers for the treatment assignment. This list will reside on the local computers at the DCC and a mirror copy (encrypted) will be kept in a different secured location. As soon as an allocation is done, the information will be sent automatically to the server and the master randomization file will be updated accordingly.

A list of subject Study ID numbers will be created a-priori for each clinical center. At the baseline visit, when a new participant is enrolled, a new Study ID will be automatically generated through a secured link in the study's Web-based system. In the randomization

visit, the computer system will generate the treatment assignment for the current participant and the Bottle ID number for Study Pill will appear according to the randomization scheme. A subject will not be randomized without an indication of a signed informed consent and a confirmed eligibility.

There are several layers of masking mechanisms. The Study ID numbers are generated automatically, and the clinical center staff will not be able to obtain IDs without first enrolling a participant; the randomized treatment assignment will also take place automatically without any possibility of change at the clinical site end. The boxes containing the Study Pills are pre-packaged with identically looking pill bottles containing either aspirin or placebo. During the entire duration of the study, neither the participant nor the clinical centers' personnel will have any access to data that will enable de-coding the randomization scheme. Unblinding procedures are in place as needed but are anticipated to occur rarely.

The automated randomization scheme allows the process to take place in distant geographical locations as it does not require direct contact with a DCC staff member. However, in case any problems arise, the Haifa DCC operates a telephone hotline for reporting any urgent problems allowing for quick resolution.

D.6. Active follow-up

The active phase of follow-up will last for up to two menstrual cycles of participants and will entail collection of the following data: daily urine specimens, blood samples at mid-cycle 1, end-cycle 1, mid-cycle 2, end-cycle 2 by venipuncture, and daily diaries.
Active enrollment also entails collection of brief questionnaire data to evaluate adherence to treatment and the occurrence of possible adverse reactions to the Study Pill and folic acid given on trial. Once a women tests positive on a pregnancy test she will transition into the pregnancy follow-up schedule described in section D.8. In the following description of the non-pregnancy follow-up we use the terms "cycle" and "month" interchangeably. However, the exact study duration for each woman in this phase will be based on her cycle length and is therefore variable among participants.

D.6a. Active Follow-up Visits:

The first active follow-up activities are performed at the randomization visit (V-RD). The date of this visit will be scheduled to coincide with day 3 (day 2-4) of the participant's expected next menstrual cycle. In addition to the activities described above for the randomization visit (Section D5), the following activities will be performed at this visit:

- Again review the study requirements, answer any participant questions and review a brief update of eligibility criteria (any changes since baseline visit).
- Review part A of the Health and Reproductive History Questionnaire, the Family Medical History Questionnaire, and the Personal Information Questionnaire that the participant will have completed at home prior to the visit
- Provide the daily diary and instructions on how to complete it. Participants will be asked to complete the diary for the previous day and this will be reviewed with them to ensure understanding and completeness.
- Blood and urine samples will be collected.

- Provide the participant with the Clearblue Fertility Monitor (and testing sticks) and urine collection materials and review instructions for the use of the fertility monitors as well as instructions for home urine collection and storage.
- Provide study bags and a small cooler to be used for transport of diaries, monitors and home urine specimens.
- Provide the participant with a 3-month supply of Study Pill and folic acid. Instruct participants to bring all pill bottles back to each visit whether or not empty.
- Schedule the next visit to correspond to mid-cycle, about day 14 of a 28 day cycle (adjusted for cycle length). They will be asked to bring in their diaries and monitors and all urine samples collected in to the visit.

Between these active follow up appointments, participants will collect daily urine specimens (self storage by patient), complete daily diaries, and conduct fertility monitoring.

Specimen collection
First morning urine specimens will be collected daily by the women during follow-up in order to measure levels of human chorionic gonadotropin (hCG), as well as other relevant hormones. All women will begin collecting urine on day 3 (2-4) of their first study menstrual cycle (at the randomization visit) and continue collection at home through active follow-up or until they become pregnant. A two-week supply of pre-labeled urine specimen kits will be given to each participant at the beginning of the study and they will be resupplied as necessary. All specimens will be maintained in the participant's freezer and collected approximately every two weeks at the time of clinic visit where serum collection will also be done. Serum will be collected every two weeks during active follow-up on approximately the 14th and 3rd day of each cycle. During pregnancy, serum and urine will be collected according to study protocols (Section D.8).

The main purpose in collecting daily urine specimens during the active phase (and first month of pregnancy) is to capture early, sub-clinical losses by quantitative hCG testing. Qualitative hCG determinations will be used to document pregnancy, implantation, and early pregnancy loss. These data, taken together with clinical and ultrasound data, as well as serum hCG, will help categorize outcomes into biochemical or clinical pregnancies.

Stored serial urine samples can also be used retrospectively for determination of the LH surge and the progesterone metabolite pregnanediol glucuronide as a urinary marker of ovulation. These measurements promise more accurate laboratory determinations of LH surge than the Clearblue Fertility Monitor can provide. About 9% of cycles with the Clearblue Monitor do not have a clearly identified LH surge. Stored urine samples also will be used to assess treatment compliance (by assessing aspirin metabolites), physiologic potency of the aspirin therapy (by assessing eicosanoid metabolism), and as a repository for future assays.

Specimen repository
Samples collected that are not tested as part of the current protocol will be used to generate a specimen repository. All specimens will be processed and transported to the NICHD in a schedule according to study protocols.

D.6b. Active Phase Diaries (See Appendix 4)
Daily Diaries: Variables to be recorded daily during active conception attempts

- Use and results of the Clearblue Fertility Monitor
- Taking of study pills and folic acid (adherence measure)
- Bleeding or allergic signs or symptoms referable to the use of aspirin
- Intercourse occurrence
- Menstrual bleeding
- Unusual observed pelvic complaints such as pain and bleeding
- The use of self-imposed formulas, herbs or pharmaceuticals for conception (or contraception) attempts
- Concurrent illness
- Concurrent medications used
- Alcohol, tobacco and caffeine exposure
- Stress

D.6c. Active Visit 1 (Month 1, Mid-Cycle) (V-A1): At the mid-cycle visit for month 1 (about day 14 of a 28 day cycle) we will again review the study requirements and answer any participant questions. In addition we will:

- Collect home (frozen) urine specimens from participants; provide participant with another kit for the next two weeks of home urine collection.
- Collect in clinic blood and urine specimens.
- Collect and review daily diaries.
- Do an assessment of adherence using the Adherence questionnaire (See Appendix 5) and weighing pill bottles– address any problems reported.
- Weigh pill bottles to verify self-report and then return bottles.
- Conduct a safety interview regarding aspirin use (See Appendix 5).
- Collect information from fertility monitors (and discuss any issues found regarding daily use or other problems detected).
- Review instructions for next reporting period for urine collection, monitor and diaries.
- Schedule next appointment (for the estimated end of cycle date + 3 days to coincide with day 3 of the next menstrual cycle).

D.6d. Active Visit 2 (Month 1, End-Cycle) (V-A2): At the end-cycle visit for month 1 (day 3 [day 2-4] of the next menstrual cycle) we will again review the study requirements and answer any participant questions. In addition to activities done at the mid-cycle 1 visit (V-A2), we will verify pill adherence by weighing the pill bottles and will do a pregnancy test on collected urine (only if the participant has not started menstruating by this visit). The visit will include:

- Collect home (frozen) urine specimens from participants.
- Collect in clinic blood and urine specimens.
- Pregnancy test (urine stick) in clinic only if the participant has not started menstruating by this visit.*
- Collect and review daily diaries.

- Do a verbal assessment of adherence – address any problems reported (see Appendix 5).
- Do bottle weighing to verify self-report and return pill bottles.
- Conduct a safety interview.
- Collect information from fertility monitors (and discuss any issues found regarding daily use or other problems detected).
- Review daily diaries.
- Review instructions for next reporting period (urine collection, monitor use and diaries).
- *If the pregnancy test is negative, we will provide an updated calendar for testing and schedule next appointment (for about the mid-point of cycle 2). These subjects will have one additional month of active follow-up (see Active Month 2). HOWEVER, if the urine stick test is positive, subjects will transition in to pregnancy follow-up. Those subjects will be seen according to 1st Pregnancy Month visit schedule.
- If the participant has not started menstruating by this visit but the clinic pregnancy test is negative, she will be instructed to take a home pregnancy test (HPT) 5 days later. If the HPT is positive, she will need to return to the clinic within 3 days to start the pregnancy follow-up. If the HPT is negative (or she starts menstruating before the 5 days), the participant will continue as previously scheduled. If she does menstruate this month and continues to have a negative HPT, she will be considered as having an anovulatory cycle.

D.6e. Active Visit 3 (Month 2, Mid-Cycle (V-A3) (for subjects not pregnant in month 1) (V-A3): At the mid-cycle visit for month 2 (about day 14 of a second 28 day cycle) the protocol for V-A1(Month 1,Mid-Cycle visit) will be performed again (see above, D.6c).

D.6f. Active Visit 4 (Month 2, End-Cycle) (V-A4): The end-cycle visit for month 2 occurs on the expected day 3 (day 2-4) of the next menstrual cycle. The visit will include all activities as listed in Month 1, End-Cycle visit **V-A2** (see above, D.6d). In addition to the above listed V-A2 activities, participants will also be provided with another 3-month supply of Study Pills and folic acid (note: pills are provided one month in advance so that participants always have at least a one month supply). Participants will return the final daily diaries and home urine specimens. If pregnant, participants will transition into pregnancy follow-up. If not pregnant, participants will be instructed on the requirements for passive follow-up. At this visit, participants will be provided with a HPT and at least three days of emergency collection tubes to be used in case of a positive HPT. The next telephone contact and the following End-Cycle passive follow-up clinic visit will be scheduled.

D.7. Passive Follow-up

Women who do not conceive during the active follow-up phase will continue with treatment and enter passive follow-up for an additional four cycles or until becoming pregnant (whichever comes first). The passive follow-up schedule is less frequent than the active schedule and has fewer demands for specimen collection and diary reporting. Participants will continue taking study pills and conducting home fertility monitoring.

They will not need to collect urine for storage and freezing at home (they will continue using the monitors). Participants will have monthly visits with study staff to collect clinic samples and data, replenish pills, and to assess safety, adherence and pregnancy.

Phone calls between visits will be done at the mid-cycle point to assess any adverse event occurrences and reinforce adherence to the study pills.

D.7a. Passive Visits (V-P1 to V-P4): At the end-cycle monthly passive visits (Month 3, 4, 5 and 6 of study follow-up) participants will come into the clinic. The visits will occur at end-cycle + 3 days of the menstrual cycle (i.e., day 3 of menstruation).

At day 1 of expected menstruation, **ALL women without menses** will take a home pregnancy test (HPT). If the HPT is positive, women will use the three emergency urine collection tubes they should have and collect first morning urine until they come in to the clinic on their regularly scheduled visit at which point they will enter into pregnancy follow-up. She will be instructed to write the date on each of the urine collection tubes. If the HPT is negative, the women will still come in at the regularly scheduled visit but will not need to collect urine.

Passive follow-up visits will include:

- Completion of the passive-follow up questionnaire (See Appendix 6)
- Collection of a urine sample. Perform a pregnancy test (urine stick) in clinic only if the woman has NOT started menstruating.
- A verbal assessment of adherence – address any problems reported.
- Pill bottle weighing to verify self-report of adherence. (Distribute additional pills at V-P4).
- Completion of a safety interview.
- Collection of information from fertility monitors (discuss issues or other problems detected).
- Review of instructions for next reporting period (e.g., continue Study Pills and folic acid, continue using fertility monitor, conduct pregnancy testing if needed, complete the phone contacts at the mid-cycle, and come in for the next clinic visit at approximately end-cycle +3 day of the next menstrual cycle).
- If the pregnancy test is negative, we will provide an updated calendar for testing and schedule the next appointment. If the urine test is positive, subjects will provide a serum specimen for banking, and they will be seen according to 1st Pregnancy Month schedule.
- Instruct participant that any time she has a positive home pregnancy test, she needs to call the study coordinator to come into the clinic within 3 days of the positive test and to start collecting and freezing first morning urines until the day of the visit. She will be instructed to include the date on each of the urine collection tubes. She will need to bring the frozen urine samples to the clinic on the visit day.

D.7b. Passive Follow-Up Phone Contacts

The study staff will call participants at mid-cycle during passive follow-up. During these calls, following the script, the staff will administer the Safety Questionnaire and the Adherence Questionnaire. We will also reinforce the importance of adherence.

At the end of passive follow-up (end of 6th menstrual cycle), if no pregnancy is confirmed, subjects will be **closed** from regular participation in the study (no additional clinic visits). The non-pregnancy follow-up scheme is depicted in Figure 2.

Figure 2. Timeline for participant follow-up during attempt to conceive *

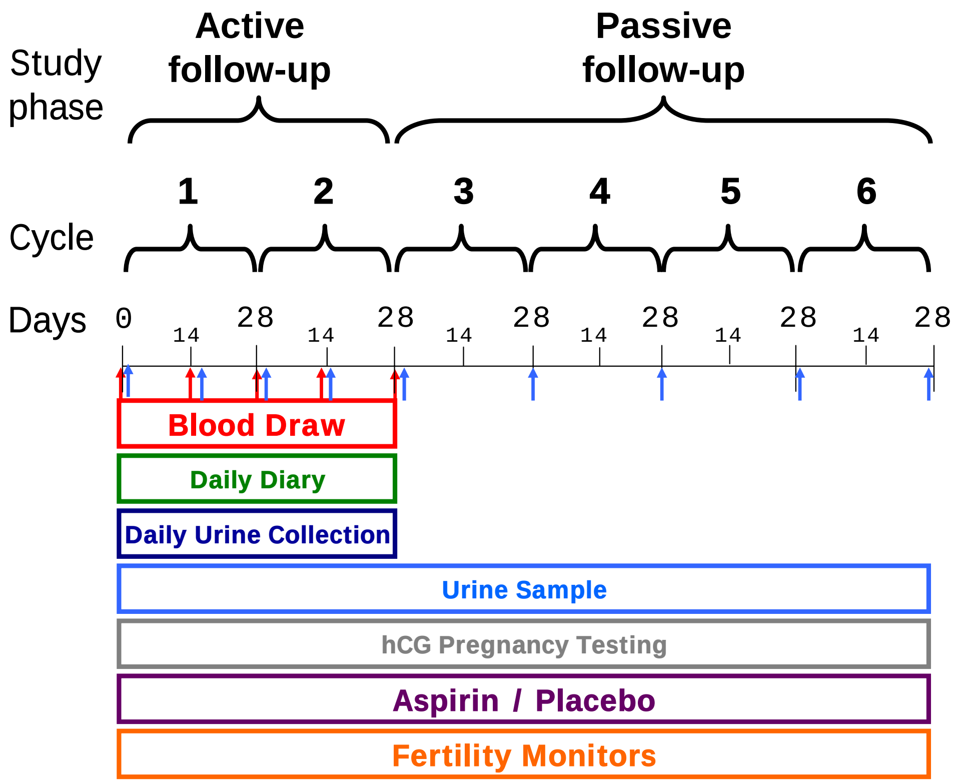

* Note: We use cycles of length 28 days as an example with day 14 indicating mid-cycle.

D.8. Pregnancy follow-up

Women will continue taking assigned study treatment assignment until 36 weeks gestation (or pregnancy outcome if sooner than 36 weeks) to avoid some potential risks associated with late aspirin use in pregnancy, including issues related to patent ductus arteriosus, bleeding with abruption and potential contraction inhibiting effects. Study personnel will actively follow participants for weeks 4-8 post detected pregnancy and then with periodic contact until the end of pregnancy. Women who experience an early hCG detected pregnancy loss (i.e., prior to clinical confirmation of pregnancy) will return to active or passive follow-up (depending on where they left off) until they have completed a total of six cycles of follow-up or become pregnant again, whichever comes first. The outline or pregnancy follow-up is described in Figure 3.

Pregnant women will be encouraged to receive prenatal care. Although this is not the responsibility of the trial, study coordinators will help facilitate access to care for willing participants without established providers.

1st Month of Pregnancy Visits: If participants become pregnant (regardless whether during active or passive follow up), they will conduct one additional month of home collection and storage of urines. In order to have samples to assess changes in urine hCG in subjects (both who miscarry and those who continue to maintain their pregnancy), we will collect urine for this entire month (30 days). During this first month, participants will also complete a pregnancy daily diary and have an early ultrasound at week 6.5 of gestation.

During pregnancy the following activities will occur:

- Telephone and personal contact with research nurses/staff to collect data, specimens, distribute pills, assess adherence, and conduct medication safety interview
- Participants will continue daily Study Pills (aspirin or placebo) and folic acid (given as three-month supply bottles) until week 36 of gestation.
- Daily home urine collection and storage for one month.
- Daily pregnancy diaries for one month.
- Completion of pregnancy follow-up questionnaire monthly either by phone or in clinic (See Appendix 7)
- Completion of Safety and Adherence Questionnaires as well as weighing the of the pill bottles to evaluate adherence in clinic visit.
- Completion of the Short Physical Measurements Form at each clinic visit
- A blood draw and urine sample at each study clinic visit.
- An additional study sonogram at 6.5 weeks of gestation.
- Obtain information from routine clinically indicated obstetric care including sonograms through medical record abstraction.
- Chart abstraction of medical records after pregnancy to assess obstetric outcome, sonograms, and prenatal laboratory values including the standard serum screen around 15-18 weeks' gestation (which is strongly correlated with placental insufficiency[128-129]).
- Maternal sera will be obtained at least once each trimester at the time of clinically indicated blood draw (initial visit, second trimester serum marker screen, glucola screen, and time of delivery (in Utah only)).
- In Utah, women will be given a container to collect tissue should they experience miscarriage during the study.

The University of Utah will perform seven visits during pregnancy based around routinely scheduled prenatal clinic visits whenever possible to minimize the burden on participants. When study visits are completed at the participant's physician's office (rather than the research clinic), all typical visit activities will still be performed except not all the information on the Short Physical Measurements Form may be available. The week 8 pregnancy visit needs to occur at the research clinic since new randomized pill

bottles will need to be obtained for the participant. This supply of pills should be enough to cover the period until week 36 of pregnancy. In Utah, the approximate times of the visits will be: week 4, 8, 12, 20, 28, 36 and parturition (See Table 1 below for timing of contacts and activities). The last active or passive follow-up visit where the positive pregnancy test was obtained is considered week 4 of pregnancy. Specimen collection will be obtained at the 8, 12, 20 and 28 week visits. If the 12, 20, and 28 week visits are done at the participant's physician's office and routine labs are to be drawn at that visit, every effort will be made to obtain the study labs at the same time. There will be no specimen collection at the 36 week visit. Utah will perform monthly phone calls at weeks 16, 24, and 32 to conduct the safety interview, adherence assessment, and the monthly pregnancy questionnaire. The study ultrasounds will be performed at separate visits at one of the four research clinic sites and will be conducted at 6 ½ weeks to confirm and date the pregnancy. Utah will also collect placenta at the time of miscarriage (when feasible) and after delivery. Cord blood will also be collected at the time of delivery when feasible.

The University at Buffalo will perform five pregnancy visits at weeks 4, 8, 12, 20, and 28. The last active or passive follow-up visit where the positive pregnancy test was obtained is considered week 4 of pregnancy. Specimen collection will be obtained at the 8, 12, 20 and 28 week visits. A study ultrasound will be performed at 6 ½ weeks to confirm and date the pregnancy. In between study visits (at weeks 16, 24, 32, and 36), Buffalo will perform monthly phone calls to conduct the safety interview, adherence assessment, and the monthly pregnancy questionnaire.

The outline or pregnancy follow-up is described in Figure 3 below.

Figure 3: Timeline for participant follow-up during pregnancy

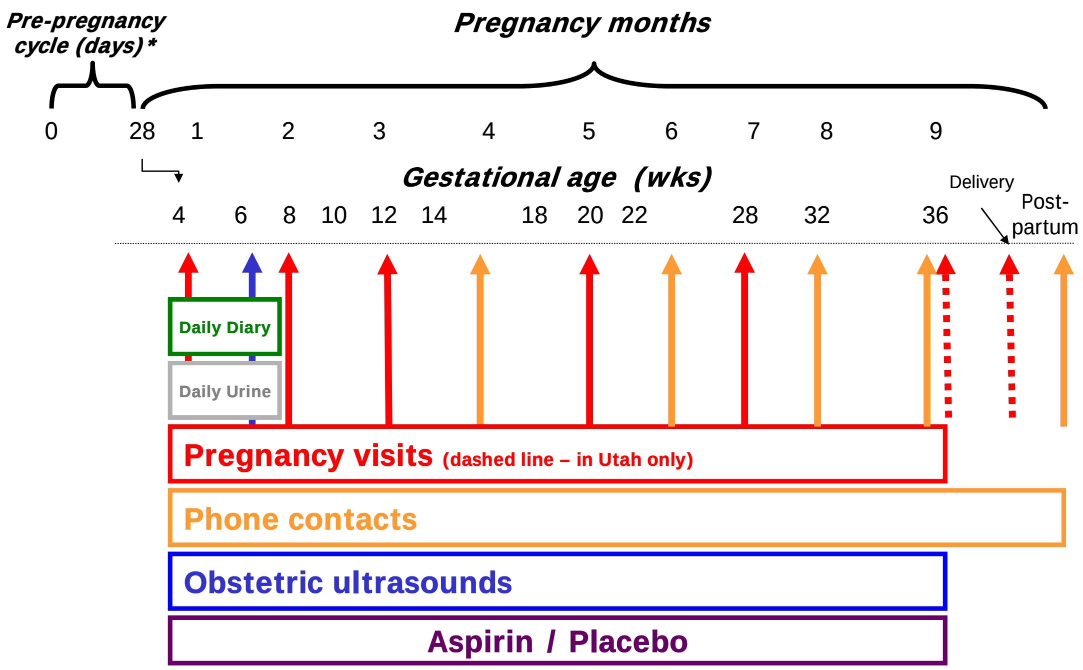

* Day 28 represent the end of cycle visit with a positive pregnancy test

Table 1. Activities performed during pregnancy follow-up visits

	Pregnancy Visits							
	Week gestational age							
**Utah & Buffalo**	4	6.5	8	12	20	28	36 (Utah)	Parturition
Blood Specimen	X		X	X	X	X		X (Utah)
Urine Specimen	X		X	X	X	X		
Home Daily Urine Collection	X	→	X					
Pregnancy Daily Diaries	x	→	x					
Study Ultrasound		X			X (Utah)			
Blood Pressure	X		X	X	X	X	X	
Anthropometric Measurements	X		X	X	X	X	X	

Pregnancy Questionnaire			X	X	X	X	
Safety Interview	X	X	X	X	X	X	
Adherence Questionnaire	X	X	X	X	X	X	
Placenta/Cord Blood Collection							**X (Utah)**

Closeout of Pregnant Participants and Outcome Ascertainment:
Closeout will occur for all pregnant subjects within six to eight weeks of their final outcome (miscarriage, stillbirth, live birth, other). A postpartum questionnaire will be conducted over the phone approximately six weeks after the participant's endpoint occurrence to obtain information about late complications for mother and child. Closeout will occur after the completion of the chart abstraction form for the course of pregnancy, delivery and late complications.

Miscarriage: For those experiencing SA information on dates, symptoms, procedures and location of visits to physician(s) will be asked. Where applicable, pathology records will be requested. A release(s) for records from their personal physician, any testing or treatment centers will also be secured to verify outcomes reported and dates. Information on support groups or counseling will be provided to these participants. The University of Utah will be performing karyotyping of the miscarriage tissues where tissue is available.

Stillbirth: For those with pregnancy loss after 20 weeks (stillbirth), information on dates, symptoms, procedures and location of visits to physician(s) will be asked. Where applicable, pathology records will be requested. A release(s) for records from their personal physician, any testing or treatment centers will also be secured to verify outcomes reported and dates. Complete information on the course of this pregnancy outcome will be abstracted from the physician and hospital records including information on both maternal and fetal complications. Information on support groups or counseling will be provided to these participants.

Live birth: For those who have a live birth, regardless of gestational age, information on dates, course of delivery, procedures and location of birth will be asked. A release(s) for records from their personal physician, any testing or treatment centers will also be secured to verify outcomes reported and dates. Complete information from hospital and physician records regarding gestational age, fetal growth, birth defects and type of delivery will be abstracted. Information on both the mother and the infant will be collected from the records using standardized abstraction forms. Those live births with complications occurring in either the mother and/or infant will be treated with careful sensitivity. Information on support groups or counseling will be provided to participants who have negative events occurring during or after delivery.

D.9. Specimen Collection

All blood, tissue and urine specimen (including clinic urine samples and the daily home urine samples) collected during the EAGeR study will be initially processed and stored at the local sites in -80F freezers. .

Later, ALL specimens collected from the clinical sites will be sent to the NIH Tissue Repository (Fisher Bioservices: 627 Lofstrand Lane, Rockville, MD 20850 – 301-762-1772). The exact schedule and protocol for shipping the specimen to the central repository will be determined at a later date, taking into account the rate of enrollment and the storage capacity at each center.

D10. Retention Plan and Compensation

D.10a. Retention Plan: Retention of the research subjects will be one of the most difficult tasks of this study. There are frequent clinical visits that require blood and urine collection at each. Timing of these visits is important. We are enrolling subjects of an age that include many working women and mothers. As such, we need to minimize burden as much as possible. Our belief is that retention begins at the time of recruitment. At recruitment it is essential to establish a positive rapport with the research subjects. This includes providing information in a friendly manner, being responsive to their concerns, providing subjects with enough detail to understand the requirements of participation and stressing the importance of the potential research findings. For this study, the importance of timing and frequency of study visits and sample collection need to be stressed clearly. Awareness of the time commitment will be integral. The following steps will be taken:

1. **Retention begins with recruitment.** Spending time at recruitment to fully explain the time commitment and importance of the study. Enroll only those subjects who understand the commitment. An informed participant is the best participant.
2. **Limit burden where possible.** Assess every aspect of the study to find ways to limit participant burden without compromising the study goals. Limit questionnaires and procedures to the most relevant measurable items.
3. **Provide an inviting research environment.** Staff need to be fully accommodating and friendly. The clinical space should be clean and professional. Make the participant feel welcome and at home in the clinic.
4. **Be on time.** Never make your participants wait. Show them that their time is valuable to you!
5. **Provide easy access to the clinic and close convenient parking.** Easy access for parking and using public transportation is a must.
6. **Listen.** Listening to their concerns is a key step in finding a solution. Keep communication open. Our staff have all been trained on *motivational interviewing*, a technique used to aid in retention of subjects having difficulty. MI allows the participant to reevaluate reasons for participation and negotiate their own plan to get back on goal.
7. **Obtain additional contact information.** Obtain a phone number of a friend or relative of the subject (one they are comfortable with), so that if they move or change their number, they can still be contacted.
8. **Incentives.** We use all sorts of inexpensive incentives to keep participants on track. They are fun and intended to reward subjects. Some examples are “gold stars” for those who complete forms, small tokens such as pens, refrigerator magnets bags or other items as rewards for participation, recognizing

anniversaries, and follow-up notes that are personalized thanking them for their valuable time.

D.10b. Compensation:

Participant's compensation for the EAGeR trial will be site specific.

University of Utah
Participant compensation

Participants will receive a total of up to $ 250.00 remuneration for participation in the study. This will be distributed on a serial basis so as to avoid coercion and reward compliance as outlined below.

1. Baseline: $10.00 upon completion.
2. Enrollment: $40.00 upon completion. This will be paid after completion of the randomization visit.
3. Pregnancy: $100.00 upon becoming pregnant. Must have a positive pregnancy test at the clinic site and return the fertility monitor to receive payment. Will be paid for one positive pregnancy while in the study.
4. Completion: $100.00 upon completion of the study. Must turn in fertility monitor to receive payment.
5. Mileage reimbursement: $0.485/mile or the federal allowance rate for study visits at the site with the research nurse.

The participant will be required to sign the participant reimbursement form at each study visit. This form will be kept in the patient chart.

University of Buffalo
Participant compensation
In order to increase both recruitment rate and retention, all subjects will be compensated for their travel expenses. During this time they will also be required to keep diaries, return questionnaires and provide blood and urine samples. This is considered exceptional time burden over a fairly condensed period of time. The amount of money we offer is modest ($10 per clinic visit) and includes payment for participant travel expenses and effort. These payments will be processed every 3 months. Participants will complete vouchers at the end of each visit (required by University rules).

D.11 Sub-study

One sub-study will be conducted in the EAGeR trial to evaluate compliance with Study Pills. The sub-study will be based on analysis of urine samples for the presence of aspirin metabolites. The evaluations will be done on a 5% sample of the women participating in the study; that is about 80 samples in total. The sampling scheme will be developed by the DCC. For the sub-study to provide the most useful information, the sampling plan needs to consider possible variability in compliance among subjects, over

time and with pregnancy status of the woman. We plan to sample 30 participants in month 2, 30 in month 4 of the non-pregnancy follow-up and 20 in the second month of pregnancy. The sample will include women in both treatment arms with a ratio of about 4-5:1 of aspirin versus placebo. This condition will help maintain the masking of treatment allocation and also allow compliance evaluation for women in the placebo arm who may be taking aspirin independently. The sampling scheme will also allow for weighting of probabilities of selection by the reported adherence of the participants.

E. STUDY TIMELINE

The study duration is five years. The first 6-8 months are considered as Phase I of the study and are used for study set-up, developing and writing of the study protocol, MOP, questionnaires and developing the study's web-site and computerized data entry system and securing IRB approval. This time will also be used to obtain the study pills and all the materials needed for the study as well as the recruitment materials. Phase II of the study starts with recruitment and continues with participants' follow-up and data collection until the end of months 57. The last three months of the trial are Phase III which is the wrap-up time used for final analyses and writing of reports. The timing of the segments of the study from the start of funding is shown in Table 2 below.

Table 2. Study phases and activities by months from start of funding

		**Phase**										
		**I**						**II**				**III**
		**Year 1**		**Year 2**		**Year 3**		**Year 4**		**Year 5**		
	Time (Mos.)	**0-6**	**7-12**	**13-18**	**19-24**	**25-30**	**31-36**	**37-42**	**43-48**	**49-54**	**55-57**	**58-60**
Task												
Set-up and training	0-6											
Recruitment	7-40											
Active follow-up	8-43											
Passive follow-up	10-47											
Pregnancy f-u	8-57											
Close out	58-60											

F. STUDY ADMINISTRATION

Groups and subcommittees

The Steering Committee

The Steering Committee for the trial provides leadership in ensuring the scientific integrity of the study, while conforming to the ethics and standards of practice promulgated by the American College of Epidemiology. This is in addition to adherence to protection of human subjects according to the specifications of the authorizing IRBs. The steering committee includes the principle investigators at the clinical sites and the DCC as well as the Project Officer at NICHD. This committee will make all final protocol decisions.

Data and Safety Monitoring Board

A Data and Safety Monitoring Board (DSMB) will be established to provide independent guidance to the study conduct. The members of the DSMB will include experts in relevant fields, potentially to include endocrinology, fertility, reproductive epidemiology, biostatistics and bioethics. DSMB members are appointed by the Director of the NICHD. The DSMB reports to the NICHD director through the NICHD Project Officer and the DESPR Director. The DSMB is charged with reviewing all interim data for safety and efficacy and for recommending to the Project officer any actions that should be taken to insure the ethical conduct of the trial. Results of all such reviews will be available to the investigators upon completion of the trial and unblinding. Final decisions for study continuation or discontinuation will be made by the NICHD Director. The DSMB will also have to provide periodic reports for submission to site IRB's stating the study continues and other necessary updates.

Other study committees

Additional special committees including endpoints committee and publishing committee will be established during the evolution of the trial.

G. STATISTICAL ANALYSIS

G.1 Overview

The EAGeR trial is designed as a multi-site randomized double blind clinical trial with two treatment arms: folic acid + aspirin compared to folic acid + placebo. A fixed sample size of 1600 randomized complete subjects is planned. Thus, for planning and statistical power computations we assume that this is the number of subjects who complete the trial and does not include dropouts.

The primary analysis plan is based on an "intention-to-treat" approach comparing the two treatment arms based on the randomized assignment, ignoring later changes in treatment. This analysis is described in Section G.2. The Haifa DCC will perform periodic partial analyses and present interim reports to the DSMB as requested, during Phase II of the trial. It is anticipated that partial analyses will be performed every 6-12 months. The final analysis will be performed upon completion of data collection and editing in Phase III of the trial. Also one full formal interim analysis is planned and the power calculations with considerations for the choice of optimal time for the analysis are given in Section G.3.

In addition to the intention to treat analysis, the Haifa DCC will engage in methodological research with the Project Officer at the NICHD. The focus is on developing statistical methods for clinical prediction models of reproductive outcomes as described in Section G.4, analysis of intermediate endpoints (Section G.5) and evaluating the effect of dropouts and compliance on the study results (Section G.6).

G.2 Study endpoints and intention to treat analysis

G.2.1 Study endpoints: operational definition and verification

Study endpoints will be determined based on self-reports, self-administered pregnancy tests, clinic pregnancy tests, medical record evaluations (including routine pregnancy follow-up, ultrasound) and laboratory tests. Whenever possible, we will verify the endpoints using different sources of information. For example, a report by a woman of a positive pregnancy test will be verified against the laboratory results for urine hCG around the same date of the self-reported pregnancy. Similarly, self-reports of pregnancy loss will be ascertained by medical records (in case of a clinically recognized pregnancy) or by blood and urine tests around the same time point (in cases of early loss <12 weeks' gestation).

Operational definitions for a study outcome events are detailed in Section C.4. The sources of information for study endpoints ascertainment:

a) Live birth – based on hospital records
b) hCG detected pregnancy (implantation) – a self-report of a positive home pregnancy test followed by a spot urine test in the clinic and quantitative hCG evaluations in urine and serum specimens.

c) Early loss of pregnancy (ELP) – hCG pregnancy loss will be defined as a rise of urinary HCG to >0.025 ng/mL for at least 3 consecutive days followed by a decline. The rise and fall of HCG will be evaluated during the 10 days prior to the next menstrual period through day 5 of the next menstrual period. Clinically recognized pregnancy losses will be identified based on self-report followed by confirmation on ultrasound and medical records,
d) Specific pregnancy outcomes: Cesarean section, preeclampsia, gestational age, preterm birth birthweight [small for gestational age], major neonatal complications or death, and severe post-partum maternal morbidity will be determined based on hospital records and medical chart abstraction.

All events will be reviewed and verified by a central outcome review that will be performed by the NICHD Project Officer and the Principal Investigators at the study clinical sites and the Data Coordinating Center.

G2.2 Intention to treat analysis

The primary analysis of the clinical trial will be "intent to treat" (ITT) analysis based on the total cohort of randomized patients. This approach will be applied to the primary endpoint (live birth) as well as designated secondary endpoints. The difference between the incidence rates of live births for the two treatment groups will be examined by a two-sided test based on the standard Z-score (using the normal approximation to the binomial distribution). The common null hypothesis states that the effect of aspirin on the outcome is null compared to the placebo.

The secondary objectives (as described in Section A) with binary endpoints will be analyzed similarly as comparison of proportions. For binary outcomes such as pregnancy, the time to event may be of interest. In this case, survival analysis methods will be applied with the log-rank test for comparison of the two study treatment groups. For continuous outcome such as birthweight either parametric or non-parametric methods will be used as appropriate. If transformations to approximate a normal distribution are feasible, they will be carried out prior to the parametric analysis. The normality of the data will be examined both graphically using Q-Q plots and by formal tests (Wilks- Shapiro test). For data (either on original or transformed scale) assumed to be normally distributed the comparison of the two treatment groups will be carried out by a t-test. Alternatively if a non-parametric test is required, the Wilcoxon-Mann-Whitney test will be used.

G.3 Interim analysis

For a multi-year trial such as this one it is useful to examine the data as it accumulates in order to see if the study should be ended early or to catch any surprising developments. Such interim analysis needs to be planned formally. One of the most widely used procedures for interim analysis of a clinical trial is the α spending function approach of Lan and DeMets.[98-99] This method uses a function of time to specify the rate at which the total Type I error probability is to be spent during the trial interim looks so that this quantity does not exceed the desired overall Alpha level. For the EAGeR trial the spending function corresponding to the O'Brien-Fleming procedure will be used. The O'Brien- Fleming boundaries were chosen since for a given sample size they do not significantly change the overall power and are conservative early in the trial. By

conservative we mean that the proportions of success must be extremely different between the treatment arms to result in statistical significance early in the trial.

For our implementation the rate at which Type I error is spent is a function of elapsed time. We consider live birth as the primary trial endpoint and can expect events to start appearing approximately 10 months after subjects are randomized, that is month 16 of the trial. The accrual period will continue through month 40 while Phase II ends by month 57. Thus, for the interim analysis we consider the time interval of 16-56 month as the period when the primary outcome of live birth accumulates. Realistically though, only interim looks prior to month 40 of the trial will affect future recruitment while looks between 40 and 56 months can only affect decisions regarding continuing active or passive follow-up of subjects already enrolled in the trial.

The following Tables 3 and 4 present results of power calculations for various interim looks plans for the EAGeR trial. These demonstrate the implications of different choices for the number and timing of interim looks. We assume throughout an alpha level of 0.05 and a sample size of 1600 women divided equally to the aspirin and placebo arms of the trial. Previously published studies[100] suggest that for the control group a probability of about 0.70 - 0.80 for live birth given pregnancy (P_{preg}) can be expected. Further, the probability of implantation per cycle ($P_{implant}$) was assumed to be constant and in the range between 0.175 and 0.25. Figure 4 shows the probability of live birth (PLB) considering six cycles of menstruation (including both active and passive follow-up):

$$\mathrm{P}LB = \mathrm{P}_{preg}\, P_{implant} \sum_{c=1}^{6} (1 - P_{implant})^{c-1}$$

Figure 4. Probability of live birth versus implantation probability per menstrual cycle

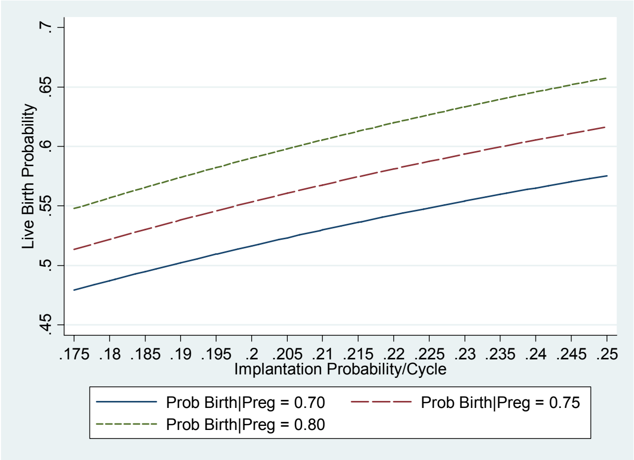

Hence a conservative estimate for the live birth rate would be a combination of implantation rate or 17.5% per cycle and a probability of live birth given pregnancy of 70% yielding an estimated value of PLB = 0.479. The final calculations will be done after the population from which participants are drawn will be identified. A refinement of the calculation can easily incorporate non-fixed probabilities of implantation per cycle

In the following, we consider a range of probabilities for the placebo + folic acid arm of 0.46, 0.48 and 0.50. The effect of the intervention will be measured as the rate ratio (RR) of live birth in the aspirin + folic acid arm compared to placebo + folic acid. We consider effect sizes of 1.15, 1.20 and 1.25. The calculations were preformed using PASS software. Table 3 presents the resulting power for no interim looks and Table 4 shows the power for one or two interim looks for selected outcome probabilities and effect size.

Table 3. Power for comparing live birth rates in treatment and placebo groups with no interim looks.

Live Birth Probability in placebo arm	Live Birth Rate Ratio to be Detected		
	1.15	1.20	1.25
0.46	.77	.96	.99
0.48	.81	**.97**	1.0
0.50	.84	.98	1.0

Table 3 shows for example that with 800 women in each treatment arm, we have >80% power to detect effects ≥ 1.15 for an assumed overall live birth rate of 0.48 in the placebo group.

Table 4 gives the power for one or two interim looks and the Z-score boundaries, assuming PLB = 0.48 in the placebo arm and a rate ratio of 1.20. For the period 16 – 56 months when live births accumulate, an interim look at 0.45 and 0.6 of the way correspond to 18 and 24 months into the planned recruitment period. In other words, stopping at month 18 of recruitment (24th month of the trial) will "save" the last 6 months of recruitment while by 24 months of recruitment (30th month of the trial) the target sample size should be reached and the stopping will apply to cessation of follow-up and data collection. Alternatively if at this stage aspirin is shown to be effective one could switch the placebo patients to aspirin and continue the follow-up. The decision to stop early will not be made solely on the basis of these statistical tests but will be made by the DSMB taking into account all available information including evaluation of secondary outcomes and ethical issues.

Table 4. Power for one or two interim looks assuming PLB = 0.48, RR = 1.2

Number of interim looks	Time of interim looks	Boundaries	Power
1	0.45<br>1.00	±2.571<br>±2.010	0.490<br>0.964
1	0.60<br>1.00	±2.366<br>±2.038	0.718<br>0.963
2	0.45<br>0.60<br>1.00	±2.571<br>±2.485<br>±2.050	0.490<br>0.696<br>0.962

Thus for example, compared to the naive power of 0.97, one interim look at 0.45 of events accumulation period will results in a very small loss of total power to 0.964

Even with two interim looks there is very little power loss. Two interim looks at 18 and 24 months into the planned recruitment period yield a high total power of 0.962 and is the proposed plan for the trial.

The high power shown for assessing an effect size of RR =1.2 for the primary outcome of the EAGeR trial indicate that more modest effects could be detected with high probability.

The Lan-DeMets methodology is flexible in that the total number and timing of the interim looks does not need to be specified in advance. Based on results of periodic partial analyses additional looks can be planned and the test boundaries can be adjusted according to the spending function.

G.4 Prediction models and secondary analyses

In addition to the ITT analysis the data collected in the EAGeR trial will be utilized to develop prediction models for various endpoints in the gestation and reproductive process. These models will enhance our understanding of these processes and the factors that may affect outcomes. In that, the study will provide important information beyond the evaluation of the effects of LDA.

The prediction models approach is described here only briefly.

Prediction of live birth

Various prediction models can be built for this binary event considering baseline and follow-up information. Since women were randomized to receive one of two treatments, we will initially develop separate models for the groups based on the intention to treat assignment with the population at risk being the complete study population. We propose a few modeling approaches as an example.

A model to predict the probability of live birth may be developed based on the information collected at the baseline visit such as demographic information, medical history, and time since last spontaneous abortion. Three methods can be used to build such a model: (a) Logistic regression (b) Neural networks (c) Regression trees. While logistic regression is a standard technique, the other two methods will allow us to consider more complicated associations between risk factors and outcome (e.g. interactions and optimal cut-off points) as well as discrete outcomes with several possible categories (e.g. early pregnancy loss, pregnancy complications, birth). These aspects may be especially interesting for the EAGeR trial where a number of sequentially occurring endpoints are evaluated.

Validation methods will be used to compare these approaches whereby the data will be divided randomly into a training (e.g. 2/3 of the data) and validation sets (e.g. 1/3 of the data). The models will be fit using the training set and their predictive ability will be compared on the validation set. ROC (receiving operating characteristic) curves will be used to compare the different models and determine which is to be preferred.[101-102] The optimal probability threshold for the logistic and neural network models will be chosen using the Youden Index.[103-105]

In addition to the models using only baseline information there is interest in considering additional clinical and biomarker information gathered during follow-up. In particular, assessing the influence of true exposure to treatment, taking into consideration compliance to assigned treatment and additional aspirin use. Compliance would need to be accounted for either in a cumulative manner (e.g. >80% compliance over time) or by considering lagged variables with compliance being measured over specified time intervals (e.g. trimesters of pregnancy). In contrast to prediction models based on baseline information, such models would be restricted to prediction for subjects who have reached various stages, for example examining the effect of compliance in say the first trimester of pregnancy conditional on completing the first trimester. For these analyses the sample sizes will be lower and models will include covariates. Therefore, specific power analyses will be conducted to assess the power available for testing specific hypotheses of interest.

Other analytic approaches including survival analysis will be applied for secondary endpoints such as time to becoming pregnant or time from hCG detected pregnancy to early pregnancy loss (EPL). Both Cox regression models and neural networks will be considered for covariates adjustment. Finally, for outcomes that may be repeated over time such as symptoms assessed in the safety interview, methods of longitudinal data analysis with repeated measurements will be applied. These include marginal models, both parametric and semiparametric (e.g. generalized estimating equations approach) and random effect models.

One aspect of the study design that will be addressed carefully in the analysis is the subgroup of women who experience an hCG pregnancy loss and return to follow-up with a chance of becoming pregnant again. The rationale for including this group in the primary analysis of live birth is that typically this type of pregnancy loss would not have been detected. However, these women may contribute more than one pregnancy to the analysis (at most two) and up to two losses. These women may be different from other participants in their characteristics or in how aspirin affects them. Therefore sensitivity analysis will be performed to assess the influence of censoring these women at the time of first event on study results.

Secondary outcomes for infants' complications
For these outcomes, expected to be rare, an intention to treat analysis will be performed using Poisson regression methods to compare the overall rate of these (adverse) events in the two treatment groups. Additional analyses will use live-birth as the denominator to calculate rates.

A general note:
In general, for some analyses comparison between study arms will not be based strictly on intention to treat approach. Rather, the denominator will be defined to include only those sub-groups that are at risk for the outcome event according to their course of follow-up. For example, although live birth is the primary outcome and will be analyzed with the ITT principle, we may want to look at live birth rates out of those women who became pregnant to compare the two groups. All these additional analyses will be interpreted with caution to maintain a coherent summary of the effect of LDA on all reproduction and gestation outcomes.

G.6 Evaluation of drop outs and compliance
<u>*Evaluate the effect of drop outs and non-compliance*</u>
The Haifa DCC will co-develop with the Project Officer statistical methods for evaluation of drop out and compliance for the future analyses of the study results. Both drop-outs and non-compliance introduce a problem when the processes do not occur at random. Drop-outs potentially lead to selectively missing data that may bias crude intention-to-treat comparisons. Non-compliance to assigned treatment can also be influenced by intermediate study outcomes and therefore introduce additional bias. Marginal structural models (MSM) suggested in the causal inference framework[106-108] have been used in observational studies to account for time-dependent covariates that are on the pathway to disease outcome or self-selection into treatment.[109-110] MSM have also been useful in

clinical trial for handling non-compliance to provide unbiased estimates of the causal effect of treatment building upon the theory of causal inference and the idea of 'potential outcomes.[111-112] A key component to these models is inverse probability weighting set to model the exposure (e.g. aspirin use) with the end result providing an estimate of the relative risk of the outcome of interest among the treated group compared to placebo, with appropriate confidence intervals.

G7. Analysis for the sub-study on compliance

The sub-study to assess compliance based on laboratory assessments of the aspirin metabolite salicylate (salicylic acid) in urine samples was described above in section D10. The sampling design for this sub-study is somewhat challenging given that little data is available on the pharmacokinetic behavior of LDA metabolites in the urine. Moreover, an important question is how sensitive are salicylate levels of to non-adherence; for example, what is the effect of skipping medication for one day on this biomarker? We propose to perform first a pilot study analyzing a sequential series of daily urine samples for few individuals (on aspirin and also controls) to better understand the trajectory of aspirin metabolites in the urine over time. Compartmental models and other pharmacokinetic analyses will be applied. Based on the analysis of the pilot and the estimated time to steady state and half-life of low-dose aspirin metabolite we will plan the sampling of individuals and times for evaluation of self-reports on adherence.

G.8 Implementation of statistical analysis

Data analysis will be performed on personal computers at the Haifa DCC using SAS, STATA and R software. Questions related to data analysis and methodological issues will be discussed periodically in the weekly staff meetings at the DCC. Interim results will be summarized and presented at the DSMB meetings and discussed with the Project Officer. Holding regular discussions of study analysis is crucially important to ensure that study aims are met and that the interpretation of data is valid. Publication quality summaries of the results of the statistical analyses will be prepared by the Haifa DCC team for interim and final reports, presentations and manuscripts.

H. DATA MANAGEMENT PROCEDURES

H.1. Internet-based clinical trials

The University of Haifa DCC will develop an internet-based system for the EAGeR trial in close collaboration with the investigators and staff members at the Clinical Sites.

Over the last decade increasing numbers of randomized controlled clinical trials as well as other clinical multi-center studies are performed using internet-based technologies. Some of the advantages of such systems include minimization of errors, real time data reporting, saving in resources for recording of the data, less paper and less storage space required.[113-114]
However there are some difficulties that need to be addressed when considering an internet -based clinical trial. The development phase requires time and an understanding of the environment where the system is going to be deployed and its goals. Curley and his colleagues report on such difficulties but note that the system developed for asthma clinical studies is being successfully used.[115] Others have also noted possible difficulties in implementing the new technologies in clinical settings that lack the needed infrastructure or trained staff.[116]

.

H.2. EAGeR web-based system

The web-based system developed for the EAGeR trial is designed to provide an efficient and secure platform for data entry, management, coordination and monitoring of the trial. The system will meet the following goals:

a) Provide a state-of-the-art web-based platform for the EAGeR study to facilitate communications between all study investigators and staff to enhance the study coordination and monitoring.
b) Provide an electronic data capture (**EDC**) system that is simple to use and incorporates edit checks with full screen support.
c) Accessibility – enable the data to be easily retrieved and reorganized according to need.
d) Security – ensure the safety of the data by limiting access to authorized personnel and allowable procedures.
e) Recoverability – devise a backup system that will enable reconstruction of the data to any state at any time to allow for the re-creation of any analysis files and reports that existed in the past.
f) Audit trail – automated audit trail

H.2a Study web-site

The study website will be used for:

1) providing information for potential participants, and study subjects including a brief background on the study and contact information for the clinical sites
2) access to study roster , committees members
3) centralized data handling for participants screening and enrollment
4) obtaining randomized treatment and access the computerized forms
5) links to study documentation including protocol, MOP, forms, codebook
5) access monitoring and progress reports
6) links to newsletter, announcements, meeting minutes.

Access to the study website and the data entry system will be through dial-up, broad-band or wireless connections using available web-browsers.

H.3. Questionnaires and other data forms

All necessary forms and documentation material will be available on-line through the study website and can also be printed on location at the Clinical-sites as needed. The availability of information on line ensures that all are working with the same most updated version

Forms will be designed to be clear, concise, and convenient to use and for the web-based forms also include imbedded coding and initial error checking mechanisms. Data collection for the EAGeR trial will begin with screening, through the baseline visit, active follow-up (with daily diaries and specimens) during the first two menstrual cycles, a passive follow-up during the next four cycles and follow-up during pregnancy. Except for the self-administered daily diaries and certain other questionnaires that require participants to do additional follow-up (family history, medical history, personal information contacts), the principal mode of data collection for the EAGeR trial will be web-based.

All data forms developed will be pre-tested prior to final implementation and modifications made. Questionnaires will include brief specifications and instructions on how to complete them with detailed explanations given in the questionnaire's MOP.

Questionnaires

The study questionnaires and forms are listed in Section D above. Briefly, baseline questionnaires will elicit socio-demographic information, medical and reproductive history and health-related habits. Follow-up visits and phone questionnaires will obtain information on adherence to and safety of Study Pills and brief behavior summaries. Questionnaires are interviewer-administered and entered on a computerized form through the study web system.. A few questionnaires will be given to the participant to be self-administered but all will be reviewed by the study nurse/staff to verify understanding and completion. Paper versions of the questionnaires will be available on the study website in case the access to the web system is not possible. These forms will be entered later with a flag.

Previously completed questionnaires for the same woman will be retrievable from the system permitting on-site checking for consistency of new with old data and allowing the verification of any changes with the participant.

Daily diaries

These will be filled daily by each subject during the active follow-up period of two menstrual cycles, or until pregnancy, whichever comes first and during the first four weeks of identified pregnancy. These forms, eliciting information on treatment compliance, intercourse, exposures to caffeine, tobacco, alcohol and stress, possible adverse effects including bleeding and illnesses are designed to be simple and

convenient to encourage response. The completed forms will be collected by the Clinical Sites according to the study MOP and will then be shipped to the DCC for data entry.

Abstraction forms
Information on the course of pregnancy and delivery will be obtained through abstraction of medical records from routine pre-natal clinic visits, routine tests (e.g. blood tests, ultra-sound, amniocentesis) and any hospitalization, OBGYN, other PMD, or emergency room visits. Chart abstraction forms are formatted and will be entered on the computer web-system similar to other forms.

Biospecimen tracking
For each visit where specimens are being collected a series of barcodes is provided to be printed on labels and placed on tubes and containers per MOP. Each specimen sample before and after processing is barcoded and computerized laboratory forms are used to record the specimen handling and transport. The barcodes contain information on the Study ID number, the date and the type of specimen and tube. Mapping of the repository trays will allow the location of any specific specimen as needed. Shipment from local repository to the central study repository will be documented and performed according to the SOP for the lab specimen and in collaboration with the central repository. The specimen tracking information will be saved in separate data files at the DCC.

a. *Information collected from unscheduled report forms*

In addition to data collected during clinic visits and phone interviews there are a number of forms and reports that are used at any time during follow-up. These include the:

I. Case Report Form (CRF) - used to report any adverse or unusual events
II. Adverse Events Follow-up Form – used to update sequelae of adverse event reported in the CRF
III. Endpoint Report Form – used to report occurrence of study endpoints including pregnancy, loss or termination of pregnancy and delivery
IV. Protocol Violation Form
V. Withdrawal Form – used to report participant's early withdrawal from follow-up or stopping of Study Pills or folic acid
VI. Unscheduled Contact Form
VII. Change Form – used to make any changes in participants information that was entered before on one of the questionnaires.
VIII. Additional Pills/Barcodes Form

Data from each of these unscheduled forms is handled similarly to the visit associated form.

Report of adverse events:
For the EAGeR trial possible adverse events may include spotting or bleeding at anytime during pregnancy, vomiting and other gastrointestinal effects, intrauterine growth restriction, preterm labor, premature preterm rupture of membranes, congenital anomaly, intrauterine fetal death, placental and other birth complications, neonatal death, severe

maternal morbidity requiring hospitalization, and maternal death. It should be noted that these are symptoms that are also generally consistent with pregnancy, therefore may not be unexpected.

If a severe adverse event is identified while the woman is on follow-up, the staff will alert the site PI and complete the CRF within 2 days of becoming aware of the event. The DCC will update the Project Officer and the DSMB to discuss if any action needs to follow, including stopping of Study Pills/folic acid or unblinding to a health care provider.

Other local forms

Other study forms including informed consent and various local administrative form used for local participant management will be developed at the clinical sites in consultation with the DCC.

Data entry procedures

Procedures for data entry are defined and detailed in the MOP. Electronic forms are either a special application (e.g. screening and personal information) or a PDF writable form. Submitted forms need to have a digital signature of the person filling in the information. Information is saved in the study data base and also as a completed PDF form that can be retrieved and serve as a source document.

The Haifa DCC will develop training sessions to guide data entry staff in data entry procedures, coding schemes, and error checking ensuring completeness and accuracy of the data entered. The procedures will include instruction about conducting personal or telephone interviews, recording biospecimen tracking information and abstracted medical information.

Confidentiality procedures

Strict confidentiality procedures ensure that information collected for the EAGeR Trial could not be linked to participant's identifier except for cases where medical attention may be needed. Participants' personal information will only be kept locally at each clinical center and kept separately from the data collected during the study where only the Study ID for each subject will appear. The locator information will be kept in a secured local server with paper forms in a locked secure cabinet/area.

Quality control procedures

The DCC will perform ongoing check of all data entered newly into the database. These will be part of the SAS data management programs and will include checks for discrepancy between information obtained through different forms and/or at different follow-up times.

Ongoing assessments of adherence to the study protocol and evaluations of data quality are essential for the success of the trial. Quality control processes will be established to identify any suspected deviations from what is expected and discrepancies in information obtained from various sources. Routine error and completeness reports will be sent to the clinical center. Those will be returned to the DCC in a timely manner to resolve identified problems and correction of errors. Concise summaries of the findings of the quality control procedures will be presented periodically on the study web-site to alert all study personnel to common problems and suggest resolution as well as any resulting modifications to study protocol.

Quality control for clinical monitoring
The DCC with the NICHD will conduct annual field audits of the clinical sites to assure adherence to the study protocol and requirements. The audits will include examination of the data collection process, specimen collection and processing, data entry procedures, local data management and record keeping. In additional, more frequent audits will be executed by the local project coordinators at each center based on an audit plan developed with the DCC. This will include a sample of about 5% of forms to be entered in parallel by two staff members and checking specimen tracking information.

Data system safety and backups

Data safety
The study servers will be kept in secured locations one in the US and one at the University of Haifa. Access to the study website and database server will be restricted to authorized personnel as determined by the DCC. All transmissions of data over the internet will be encrypted. Servers will be protected by firewall. An automated audit trail will log all entries to the study web-site and the data entry system.

Backup Protocol
The two EAGeR trial study servers are synchronized simultaneously and thus provide a real-time backup for the entire database. Backup procedures will be performed daily for the servers, work stations and the personal computers used for the project. In case of a hardware or software failure, files can be retrieved to the time of their last backup, that is, the previous day. Version for the previous 30 days will be kept. Backup copies will be created on external magnetic media (e.g. tapes, DVD) once a month and global backups, performed every 3 days, will allow retrieval of data up to the previous six months. In addition, the internet-based system creates audit trails with a date-stamp copy of every transaction and therefore the current database can be reconstructed to any state that has occurred since its implementation.

Reports and newsletter

The Haifa DCC will be responsible for preparation and distribution of interim and final reports, quarterly newsletter, presentations and other publications in coordination with

the Project Officer at the NICHD and in collaboration with the study investigators. Reports will include summaries of recruitment and follow-up status, by Clinical Site, graphical and tabular presentations and results of interim statistical analysis. The reports will have clear explanations of the methods used, interpretations and point out questions or problems that need special attention.

Special reports of adverse events occurring during the course of the study will be prepared periodically and submitted to the Project Officer and the DSMB.

The quarterly newsletter with be made available on the study Web-site and contain information on the progress and performance of the clinical centers.

I. HUMAN SUBJECT PROTECTION AND CONFIDENTIALITY PROCEDURES

This section details the issues relevant to protection of human subjects involved in the EAGeR trial.

I.1. Risks and Benefits

A. Risks and Protection Against Risk

1. Procedure Risks

Study Pills and folic acid: Any drug (including aspirin) or dietary supplement (including folic acid) can have harmful side effects, especially when mixed with other products or if the participant is allergic to aspirin or folic acid. Participants who are allergic to the Study Pills or folic acid will be ineligible to participate. Research personnel will monitor participants' pill intake (including supplements and herbal remedies) through phone calls and the information provided in the Daily Diary. Aspirin use can result in serious side effects, such as bleeding ulcers, bleeding in the brain, renal failure, and some kinds of strokes. Some medical conditions, such as hypertension, bleeding disorders, uncontrolled asthma, history of gastrointestinal ulcers, and hepatic and renal disease, could increase the risk of adverse effects from aspirin; therefore, patients with these conditions will be ineligible for participation. Along with its needed effects, a dietary supplement such as folic acid may cause some unwanted effects. Although folic acid does not usually cause any side effects, it can cause fever, general weakness or discomfort, reddened skin, shortness of breath, skin rash or itching, tightness in chest, trouble breathing, and wheezing. These more serious side effects are rare and will be monitored by the research staff through telephone contact and review of Daily Diary entries. If any adverse events are identified, these will be reported to the PI to determine if the participant should discontinue the pills. Any serious adverse events will be reported to the IRB and to the DCC. The DCC will provide periodic summaries of these events to the DSMB.

Blood Sample: This procedure is likely to involve discomfort or minimal pain as well as bruising. Rarely, blood draws include clotting, infection, and fainting. Blood is drawn by certified healthcare personnel in research and/or medical facilities.

Anthropometric Measurements: There are no anticipated physical risks associated with anthropometric measurements. However, there could be some discomfort and/or embarrassment when having the measurements taken. This procedure is conducted by experienced and certified healthcare personnel to minimize this risk.

Home Urine Collection and Fertility Monitor Testing: There are no known risks associated with these procedures. These are standard tests that are easily conducted in the comfort of a participant's home.

Interview: Baseline information will be obtained from each participant addressing her sociodemographic; medical and reproductive history; somatic body type; diet; and

lifestyle. This could be uncomfortable and or embarrassing. This interview will be conducted by trained personnel experienced in interviewing participants.

Daily Diary: Recording information every day can be difficult and frustrating at times. However, the diary has been designed to collect information in a concise fashion to minimize the time spent on this component of the study.

Medical Records Review: The risks associated with this procedure are minimal and include the accidental or incidental disclosure of medical information. This risk is minimized by adhering to strict confidentiality policies (see below).

2. Subject Confidentiality

There is a risk that the participant's information from existing medical records and study data could be released unintentionally. This risk is remote, however, since all research personnel are well-trained (HIPAA and human subject protection training is required) in these issues and will not release information to anyone outside of parameters of the protocol. Data intended to be shared between study sites will be free of identifiers that would permit linkages to individual research participants and variables that could lead to deductive disclosure of the identity.

Study documents/data will be stored in well-established and secure research offices. This information will be stored in locked filing cabinets and on password-protected computers.

3. Subject Privacy

The risk of loss of privacy also exists. However, great consideration is taken when interviewing participants and performing procedures. These interviews and procedures are performed in private and comfortable settings by experienced research staff. By following these safeguards, we anticipate that the risk of compromising a woman's privacy is minimal.

4. Provisions for Intervention in the Event of Adverse Events

All procedures are performed in medical or research centers where emergency assistance is available. Personnel performing study-related procedures are trained and experienced in the procedures they are performing and can identify potential adverse effects.

B. Benefits to Subjects and Others

Women in this study may not realize a direct benefit as a result of their participation. However, during the medical evaluations and interviews, information about their medical care and pregnancy may be discovered that may not have been without participating in

this study. In this event, that information will be relayed to the woman and her referring physician so that management of these issues can be pursued.

It is possible that women may have a more positive pregnancy outcome as a result of participation in this study. However, this cannot be guaranteed.

Information derived from this study will potentially identify a new medical treatment that may significantly impact pregnancy outcomes for many women in the future.

C. Benefits and Importance of the Knowledge to be Gained

Available information shows that low-dose aspirin (LDA) can impact several aspects of reproduction including conception, implantation, early pregnancy loss, late fetal death, preeclampsia, small for gestational age (SGA) fetus, placental problems, and preterm birth. LDA is an ideal therapy; it is apparently safe, widely available, has few maternal side effects, and is inexpensive. The possibility that such a drug could have a major impact on obstetric outcome is very important. However, there is a lot to learn. It is not clear which patients would benefit from LDA, whether other things that can influence blood flow add to the effects of LDA, and what the side effects of this treatment would be. Earlier studies have only focused specific effects of LDA such as in-vitro fertilization outcome or the development of preeclampsia. Reproduction is different in that each stage is linked together. Better blood flow at the time of conception and implantation may ultimately lead to a healthier placenta during pregnancy, and a lower risk of preeclampsia, SGA, and preterm birth.

The potential risks to the participants in this study are relatively low and are reasonable in relation to the importance of the knowledge we anticipate gaining. Procedures outlined in this protocol such as anthropometric measurements, blood draws, ultrasounds, fertility monitor testing, and interviews are minimal and we do not anticipate that the risks are above those anticipated in the course of a person's daily life.

The protocol clearly identifies risks to the participants and measures have been included to the decrease the risks anticipated in this study. By identifying a new medical intervention that may be associated with positive pregnancy outcomes, these risks are reasonable within the context of the benefits to the participants and women in the future.

D. Alternative Procedures

Since there are no proven medications to minimize adverse pregnancy outcomes, there are no alternative procedures to offer to participants. Women may choose not to participate in this study and continue attempts at conceiving a pregnancy on their own or after consultation with a physician.

I.2. Protection of Human Subjects from Research Risks – Data Coordinating Center Responsibilities

The responsibility of the Data Coordinating Center (DCC) to protect human subjects from research risk will focus on the information aspects. The potential risks involved in this study include clinical ones (e.g. adverse reaction to the aspirin or folic acid), emotional ones (e.g. increased anxiety, embarrassment due to questions related to sexual behavior) and risks to their privacy. The clinical sites have developed a clear informed consent document to make sure that participants understand the study and know the risks involved, however minimal. Participants' personal information, test results and other data will be kept confidential throughout the trial and stored in secured data archives. Personal information will be kept in a locked safe with limited access to specific study staff. Interviews (personal and by telephone) and specimen collection will be conducted in privacy. Personal information is never released without prior permission from participants. Locator information is stored separately from the data identified by participants study ID number. Linking documents are kept secure by authorized staff members. Procedures to release information in case of an adverse event will be established in coordination with the Project Officer and the DSMB.

All DCC personnel will complete human subject protection training. Any harm suffered by a participant, either physical or emotional, or alleged infringement on privacy will be documented and reported to the IRB and to the DSMB.

I.3. Subject Population

A. Women and Minorities

Women 18-40 years of age will be recruited for participation this study. All women who have experienced a pregnancy loss in the last 12 months will be provided with information about the study. In addition, information about the study will be posted in physician offices in the form of "tear-off sheets" or study brochures. No efforts will be made to exclude any women based on minority, racial, or ethnic status. Given the focus of this particular study, women will be the only participants in this study.

Participants will be requested to complete a brief questionnaire at the time of informed consent that identifies ethnicity and racial designation(s). The participant will be asked for ethnicity first and then will be able to identify one more racial designations.

The trial is potentially open to all women who fit the inclusion/exclusion criteria. The DCC will work with the clinical sites to identify potential sources of data to characterize as best as possible the race/ethnicity composition of the population to be screened for participation in the trial. Typically, trial populations do not accurately represent the target population. However, effort will be made to direct the screening to allow a heterogeneous group to be included. The DCC will generate monthly summaries to each site with the age and ethnic composition of the population screened and the group recruited. In case a growing imbalance develops, the issue will be discussed with the clinical sites and with the project officer to come up with necessary changes in recruitment strategies to minimize exclusion of minorities for any reason. No sub-group analysis is planned but statistical modeling of the data will assess the impact of age and race/ethnicity on study endpoints.

B. Children

The EAGeR trial will follow participating women (age 18 or older) throughout pregnancy and obtain information about outcomes including live birth. Although children are not recruited as active participants in the trial, some information on the newborn babies will be collected. This point will be made clear in the consent form. The Haifa DCC and the clinical sites will apply the strict confidentiality procedures that will be implemented for all study data also to information collected on the newborns in the EAGeR trial.

In case a woman agrees to participate in the trial but later refuses to allow any information on the baby to be collected, a note will be made in the woman's file to remind the clinical sites not to collect that information.

Given the scope of this proposal, children under the age of 18 are not appropriate participants since the issues of reproductive health (e.g., miscarriage, fetal death, etc.) are not identified until women have reached adult age. Subjecting children to the risks, although considered minimal in this study, cannot be justified in such a way that would satisfy current DHHS regulations (45 CFR 46.406.).

C. Special Populations and Circumstances

We will not be recruiting and enrolling special classes or participants such as children, prisoners, institutionalized individuals, or others who are likely to be vulnerable populations.

This proposal does not include plans to include such special conditions such as the use of recombinant DNA molecules, human embryonic germ cells, and human embryonic stem cells.

D. Pregnant Women, Fetuses, Placenta

Women are excluded from the study if they are already pregnant. However, since this study is focused on improving the outcome of reproductive health, it will be necessary to follow women if they become pregnant during the course of the study. All stipulations outlined in 45 CFR 46.204 (Research Involving Pregnant Women and 45 CFR 46.206 (Research Involving, After Delivery, the Placenta, the Dead Fetus, or Fetal Material) will be followed to maintain research integrity and protection to the participants.

E. Exclusion if Unable to Consent

Although this is identified in the inclusion/exclusion above, it is important to reiterate that we will not enroll anyone who does not have the ability to provide effective informed consent.

I.4. Confidentiality Procedures

Confidentiality procedures:
To ensure the strict confidentiality of participants' information and data we will establish the following procedures:
a) Study IDs – upon recruitment, each subject will be assigned a study identification number (study ID). This study ID will be used by project staff on all data forms, laboratory specimens and in the study's main databases. In this manner, the identity of the participant will not be linked directly to the data collected during the study. The assignment of ID numbers will be done by the DCC using random components that will make the sequence difficult to predict.
b) A separate locator file will have the identifying information for each subject (including name and address) linked to the study ID number. This file will be kept in a secure place and will only be available to the site personnel, not the DCC.
c) Reports generated based on the collected data will not include any identifying information and thus preserve the confidentiality of the participants.
d) The only occasion where participant identity may be linked to the collected data is in case of an adverse event that requires medical intervention; these cases will be discussed with the Project Officer on a case by case basis.
e) The need to ensure strict confidentiality of the data collected from participants will be stressed as part of the training of the study personnel involved in data collection. The importance of preserving confidentiality as a moral duty of researchers will be explained. The data may include sensitive information about sexual history and contacts and other personal data such as medical history. The willingness of participants to give such information is based on the trust that this data will be used for the research purposes only. Therefore, it is essential that study staff understand this and protects the data collected not only through the study protocols but also information gathered through incidental observations of study participants.
f) The data collected during the study will be presented in an aggregated form as totals (means, medians etc) or percentages and will not include specific individual results.
h) Access to the database will be limited to authorized study staff and will be protected with a changing password.

The DCC will not have any locator information in the study database. All locator information will be located at the local clinical sites. The subjects' code number and identity will be kept in locked files in the research center of the study Principal Investigators. Only key staff and investigators will have access to the records. In order to monitor this research study, representatives from the clinical sites' Institutional Review Boards, and other federal agencies such as NIH (National Institutes of Health), and OHRP (Office of Human Research Protection) may inspect the research records. A certificate of confidentiality will be requested from the federal government and provides further confidentiality protection by authorizing study staff to protect the privacy of subjects in this study.

Data Quality Monitoring: Details of the specific plans for training in data quality monitoring are described briefly in the following paragraph.

The staff members to be trained in data quality monitoring include personnel in the clinical sites involved in collection and entry of data (including the person supervising these activities at each clinical site) as well as the personnel in the Haifa DCC. Staff members involved in data collection and recording need to understand the importance of obtaining full and accurate data as well as understand the reason for each data item collected. Written training materials will be developed by the Haifa DCC staff and used in training sessions together with the procedures manuals. The training programs will follow the outline of the manual. For each form there will be a data entry specification sheet providing specific instruction and references. Each form will be discussed so that the staff is clear about the logical flow of the questions, skip patterns, and content. The training materials will also include practice exercises to familiarize the trainees in various situations and problems that may arise in the process of collecting and recording data. The Haifa DCC will generate sample data that the trainees will need to enter into the computerized forms through the study web-site. These ‘made-up’ data will include errors and missing information that will need to be detected and resolved for the data to be entered and submitted. Similar training examples will be developed for laboratory specimen data and for abstraction of medical records. Instruction will include options for getting help and resolving data-related problems including emails to the DCC data management team.

As part of quality monitoring, there will be special attention to the uniformity of procedures for data collection across all study sites. At the end of the training session, personnel involved in data collection and entry should demonstrate complete understanding proficiency in the procedures using all data instruments and the study web-site.

In each clinical site, one person will be designated as supervisor of data collection and entry. The supervisor will be trained by the DCC staff in performing routine checks to monitor the adherence to study protocol for data collection and entry.

J. References

1. Wang X, Chen C, Wang L, et al. Conception, early pregnancy loss, and time to clinical pregnancy: a population-based prospective study. Fertil Steril 2003;79:577-84.
2. Zinaman MJ, Clegg ED, Brown CC, et al. Estimates of human fertility and pregnancy loss. Fertil Steril 1996;65:503-9.
3. Evers JH. Female subfertility. Lancet 2002;360:151-8.
4. Wilcox AJ, Weinberg CR, O'Connor JF, et al. Incidence of early loss of pregnancy. N Engl J Med 1988;319:189-94.
5. Boklage CE. Survival probability of human conceptions from fertilization to term. Int J Fertil 1990;35:75-93.
6. Silver RM, Branch DW. Sporadic and recurrent pregnancy loss. In Reece EA, Hobbins JC, eds. Medicine of the fetus and mother. Second edition. Philadelphia: J.B. Lippincott Company, 1999, 195 - 216.
7. Goswamy RK, Williams G, Steptoe PC. Decreased uterine perfusion - a cause of infertility. Hum Reprod 1988;3:955-9.
8. Battaglia C, Larocca E, Lanzani A, et al. Doppler ultrasound studies of the uterine arteries in spontaneous and IVF stimulated ovarian cycles. Gynecol Endocrinol 1990;4:245-50.
9. Steer CV, Mills C, Campbell S, et. al. The use of transvaginal color flow imaging after in vitro fertilization to identify optimum uterine conditions before embryo transfer. Fertil Steril 1992;57:372-6.
10. Abdalla HI, Brooks AA, Johnson MR, et al. Endometrial thickness: A predictor of implantation in ovum recipients? Hum Reprod 1994;9:363-5
11. Norwitz ER, Schust DJ, Fisher SJ. Implantation and the survival of early pregnancy. NEJM. 2001 Nov 8;354(19):1400-8.
12. Goldstein SR. Embryonic death in early pregnancy: a new look at the first trimester. Obstet Gynecol 1994;84:294-297.
13. Klein J, Stein Z. Epidemiology of chromosomal anomalies in spontaneous abortion: prevalence, manifestation and determinants. In: Bennett MI, Edmonds DK, eds. *Spontaneous and recurrent abortion.* Oxford, UK: Blackwell Scientific Publications, 1987:29-50.
14. Levine JS, Branch DW, Rauch J. The antiphospholipid syndrome. N Engl J Med 2002;346:752-63.
15. Silver RM, Draper ML, Lyon JL, Byrne JLB, Ashwood EA, Branch DW: Unexplained elevations of maternal serum alpha-fetoprotein in women with antiphospholipid antibodies: A harbinger of fetal death. Obstet Gynecol, 1994;83:150-5
16. Van-Horn JT, Craven C, Ward K, Branch DW, Silver RM. Histologic features of gestational tissues from women with antiphospholipid and antiphospholipid-like syndromes. Placenta 2004;25:642-8.
17. Parrazzini F, Acaia B, Faden D, et al. Antiphospholipid antibodies and recurrent abortion. Obstet Gynecol 1991;77:854-858.
18. Parke AL, Wilson D, Maier D. The prevalence of antiphospholipid antibodies in women with recurrent spontaneous abortion, women with successful pregnancies, and women who have never been pregnant. Arthritis Rheum 1991;34:1231-1235.

19. Kujovich JL. Thrombophilia and pregnancy complications. Am J Obstet Gynecol 2004;191(2):412-24.
20. Rey E, Kahn SR, David M, Shrier I. Thrombophilic disorders and fetal loss: a meta-analysis. Lancet 2003;361(9361):901-8.
21. Oshiro BT, Silver RM, Scott JR, Yu H, Branch DW. Antiphospholipid antibodies and fetal death. Obstet Gynecol 1996;87:489-93.
22. Branch DW, Silver RM, Blackwell JL, et al. Outcome of treated pregnancies in women with antiphospholipid syndrome: an update of the Utah experience. Obstet Gynecol 1992;80:614-620.
23. Infante-Rivard C, Rivard GE, Yotov WV, et al. Absence of association of thrombophilia polymorphisms with intrauterine growth restriction. N Engl J Med 2002;347:19-25.
24. Livingston JC, Barton JR, Park V, et al. Maternal and fetal inherited thrombophilias are not related to the development of severe preeclampsia. Am J Obstet Gynecol 201;185:153-7.
25. Steel SA, Pearce JM, McParland P, Chamberlain GVP. Early Doppler ultrasound screening in prediction of hypertensive disorders of pregnancy. Lancet 1990;335:1548-51.
26. Bewley S, Cooper D, Campbell S. Doppler investigation of uteroplacental blood flow resistance in the second trimester: A screening study for preeclampsia and intrauterine growth retardation. Br J Obstet Gynaecol 1991;98:871-9.
27. Frusca T, Soregaroli M, Valamonico A, et al. Doppler velocimetry of the uterine arteries in nulliparous women. Early Hum Dev 1997;48:177-85.
28. Chien PF, Arnott N, Gordon A, et al. How useful is uterine artery Doppler flow velocimetry in the prediction of preclampsia, intrauterine growth retardation and fetal death? An overview. Br J Obstet Gynaecol 2000;107:196-208.
29. Salafia CM, Pezzullo JC, Lopez-Zeno JA, et al. Placental pathologic features of preterm preeclampsia. Am J Obstet Gynecol 1995;173:1097-105.
30. Moldenhauer JS, Stanek J, Warshak C, Khoury J, Sibai B. The frequency and severity of placental findings in women with preeclampsia are gestational age dependent. Am J Obstet Gynecol 2003;189:1173-7.
31. Genbacev O, Zhou Y, Ludlow JW, Fischer SJ. Regulation of human placental development by oxygen tension. Science 1997;277:1669-72.
32. Meekins JW, Pijnenborg R, Hanssens M, et al. A study of placental bed spiral arteries and trophoblast invasion in normal and severe preeclamptic pregnancies. Br J Obstet Gynaecol 1994;101:669-74.
33. Vane JR, Botting RM. The mechanism of action of aspirin. Thromb Res 2003;110:255-8.
34. Patrono C, Coller B, Dalen JE, et al. Platelet-active drugs: the relationships among dose, effectiveness, and side effects. Chest 2004;119:39S-63S.
35. FitzGerald GA, Oates JA, Hawiger J, et al. Endogenous biosynthesis of prostacyclin and thromboxane amd platelet function during chronic administration of aspirin in man. J Clin Invest 1983;71:676-88.
36. Patrono C, Garcia-Rodriguez LA, Landolfi R, Baigent C. Low-dose aspirin for the prevention of atherothrombosis. N Engl J Med 2005;353:2373-83.
37. Landolfi R, Gennaro LD, Novarese L, Patrono C. Aspirin for the control of platelet activation and prevention of thrombosis in essential thrombocythemia and polycythemia

vera: current insights and rationale for future studies. Semin Thromb Hemost 2006;32:251-9.
38. CAST (Chinese Acute Stroke Trial) Collaborative Group. CAST: randomized placebo controlled trial of early aspirin use in 20,000 patients with acute ischaemic stroke. Lancet 1997;349:1641-9.
39. Erkan D, Merrill JT, Yazici Y, et al. High thrombosis rate after fetal loss in antiphospholipid syndrome: effective prophylaxis with aspirin. Arthritis Rheum 2001;44:1466-7.
40. CLASP Collaborative Group. Low does aspirin in pregnancy and early childhood development: follow up of the collaborative low dose aspirin study in pregnancy. Br J Obstet Gynaecol 1995;102:861-8.
41. Knight M, Duley L, Henderson-Smart DJ, King JF. Antiplatelet agents for preventing and treating preeclampsia. Cochrane Database Syst Rev 2000;CD000492.
42. Duley L, Henderson-Smart D, Knight M, King J. Antiplatelet drugs for prevention of preeclampsia and its consequences: systematic review. BMJ 2001;322:329-33.
43. Norton M, Merril J, Cooper B, et al. Neonatal complications after the administration of indomethacin for preterm labor. N Engl J Med 1993;329:1602-7.
44. Souter D, Harding J, McCowan L, et al. Antenatal indomethacin: adverse fetal effects confirmed. Aust N Z J Obstet Gynaecol 1998;38:11-6.
45. Loe SM, Sanchez-Ramos L, Kaunitz A. Assessing the neonatal safety of indomethacin tocolysis. Obstet Gynecol 2005;106:173-9.
46. Kozer E, Nifkar S, Costei A, et al. Aspirin consumption during the first trimester of pregnancy and congenital anomalies: A meta-analysis. Am J Obstet Gynecol 2002;187:1623-30.
47. Werler MM, Sheehan JE, Mitchell AA. Maternal medication use and risks of gastroschisis and small intestinal atresia. Am J Epidemiol 2002;155:26-31.
48. Norgard B, Puho E, Czeizel A, et al. Aspirin use during early pregnancy and the risk of congenital abnormalities: A population based case-control study. Am J Obstet Gynecol 2005;192:922-3.
49. Li DK, Liu L, Odouli R. Exposure to non-steroidal anti-inflammatory drugs during pregnancy and risk of miscarriage: population based cohort study. BMJ 2003;327:
50. Vermillion ST, Robinson CJ. Antiprostaglandin drugs. Obstet Gynecol Clin N Am 2005;32:501-17.
51. Silver RM, Edwin SS, Trautman MS, et al. Bacterial lipopolysaccharide mediated fetal death: Production of a newly- recognized form of inducible cyclooxygenase (COX-2) in murine decidua in response to lipopolysaccharide. J Clin Invest, 1995;95:725-31.
52. Peruzzi L, Gianoglio B, Porcellini MG, et al. Neonatal end-stage renal failure associated with maternal ingestion of cyclo-oxygenase-type-2 selective inhibitor. Lancet 1999;354:1615.
53. Landau D, Shelef I, Polacheck H, et al. Perinatal vasoconstrictive renal insufficiency associated with maternal nimesulide use. Am J Perinatol 1999;16:441-4.
54. Wada I, Hsu CC, Williams G, Macnamee MC, Brinsden PR. The benefits of low-dose aspirin therapy in women with impaired uterine perfusion during assisted conception. Hum Reprod 1994;9:1954-7.
55. Kuo Hong-Chang, Hsu Chao-Chin, Wang Shan-Tair, Huang Ko-En. Aspirin improves uterine blood flow in the peri-implantation period. J Formos Med Assoc 1997;96:253-7.

56. Rubinstein M, Marazzi A, Polak de Fried E. Low-dose aspirin treatment improves ovarian responsiveness, uterine and ovarian blood flow velocity, implantation, and pregnancy rates in patients undergoing in vitro fertilization: a prospective, randomized, double-blind placebo-controlled assay. Fertil Steril 1999;71:825-9.
57. Li D-K, Liu L, Odouli R. Exposure to non-steroidal anti-inflammatory drugs during pregnancy and risk of miscarriage: population based cohort study. BMJ 2003;327:368
58. Norman RJ, Wu R. The potential danger of COX-2 inhibitors. Fertil Steril 2004;81:493-4.
59. Hsieh Y-Y, Tsai H-D, Chang C-C, Lo H-Y, Chen C-L. Low-dose aspirin for infertile women with thin endometrium receiving intrauterine insemination: a prospective, randomized study. J Assist Reprod Genet 2000;17:174-7.
60. Duvan CI, Ozmen B, Satiroglu H, Atabekiglu CS, Berker B. Does addition of low-dose aspirin and/or steroid as a standard treatment in nonselected intracytoplasmic sperm injection cycles improve in vitro fertilization success? A randomized, prospective, placebo-controlled study. J Assist Reprod Genet 2006;23:15-21.
61. Birkenfeld a, Mukaida T, Minichiello L, Jackson M, Kase NG, Yemini M. Incidence of autoimmune antibodies in failed embryo transfer cycles. Am J Reprod Immunol 1994;31:65-8.
62. Birdsall MA, Lockwood GM, Ledger WL Antiphospholipid antibodies in women having in-vitro fertilization. Hum Reprod 1996;11:1185-9.
63. Stern C, Chamley L, Norris H, Hale L, Baker HWG. A randomized, double-blind, placebo-controlled trial of heparin and aspirin for women with in vitro fertilization implantation failure and antiphospholipid or antinuclear antibodies. Fertil Steril 2003;80:376-83.
64. Hornstein MD, Davis OK, Massey JB, Paulson RJ, Collins JA. Antiphospholipid antibodies and in vitro fertilization success: a meta-analysis. Fertil Steril 2000;73:330-333.
65. Waldenstrom U, Hellberg D, Nilsson S. Low-dose aspirin in a short regimen as standard treatment in in vitro fertilization: a randomized, prospective study. Fertil Steril 2004;81:1560-4.
66. Peaceman AM, Rehnberg KA. The effect of immunoglobulin G fractions from patients with lupus anticoagulant on placental prostacyclin and thromboxane production. Am J Obstet Gynecol 1993;169:1403-6.
67. Peaceman AM, Rehnberg KA. The effect of aspirin and indomethacin on prostacyclin and thromboxane production by placental tissue incubated with immunoglobulin G fractions from patients with lupus anticoagulant. Am J Obstet Gynecol 1995;173:1391-6.
68. Empson M, Lassere M, Craig JC, Scott JR. Recurrent pregnancy loss with antiphospholipid antibody: A systematic review of therapeutic trials. Obstet Gynecol 2002;99:135-44.
69. Empson M, Lassere M, Craig J, Scott J. Prevention of recurrent miscarriage for women with antiphsopholipid antibody or lupus anticoagulant. The Cochrane Database of Systematic Reviews 2005, Issue 2:CD002859.pub2.
70. Cowchock S, Reece EA. Do low-risk pregnant women with antiphospholipid antibodies need to be treated? Am J Obstet Gynecol 1997;176:1099-100.
71. Pattison NS, Chamley LW, Birdsall M, et al. Does aspirin have a role in improving pregnancy outcome for women with the antiphospholipid syndrome? A randomized controlled trial. Am J Obstet Gynecol 2000;183:1008-12.

72. Tulppala M, Marttunen M, Soderstrom-Anttila V, et al. Low dose aspirin in prevention of miscarriage in women with unexplained or autoimmune related recurrent miscarriage: Effect on prostacyclin and thromboxane A2 production. Hum reprod 1997;12:1567-72.
73. Farquharson RG, Quenby S, Greaves M. Antiphospholipid syndrome in pregnancy: A randomized controlled trial of treatment. Obstet Gynecol 2002;100:408-13.
74. Di Nisio M, Peters L, Middeldorp S. Anticoagulants for the treatment of recurrent pregnancy loss in women without antiphospholipid syndrome. Cochrane Database Syst Rev 2005;CD004734.
75. Gris JC, Mercier E, Quere I, et al. Low molecular weight heparin versus low-dose aspirin in women with one fetal loss and a constitutional thrombophilic disorder. Blood 2004;103:3695-99.
76. Rai R, Backos M, Baxter N, Chilcott I, Regan L. Recurrent miscarriage-an aspirin a day? Hum Reprod 2000;15:2220-3.
77. Frias AE, Luikenaar RA, Sullivan AE, et al. Poor obstetric outcome in subsequent pregnancies in women with prior fetal death. Obstet Gynecol 2004;104:521-6.
78. Branch DW, Silver RM. Criteria for antiphospholipid syndrome: early pregnancy loss, fetal loss, or recurrent pregnancy loss? Lupus 1996;5:409-13.
79. Bose P, Black S, Kadyrov M, et al. Heparin and aspirin attenuate placental apoptosis in vitro: Implications for early pregnancy failure. Am J Obstet Gynecol 2005;192:23-30.
80. Chavarria ME, Lara-Gonzalez L, Gonzalez-Gleason A, et al. Prostacyclin/thromboxane early changes in pregnancies that are complicated by preeclampsia. Am J Obstet Gynecol 2003;188:986-92.
81. Vainio M, Riutta A, Koivisto AM, Maenpaa J. Prostacyclin, thromboxane A and the effect of low-dose ASA in pregnancies at high risk for hypertensive disorders. Acta Obstet Gynecol Scand 2004;83:1119-23.
82. Yu Y, fan J, Chen XS, et al. Differential impact of prostaglandin H synthase 1 knockdown on platelets and parturition. J Clin Invest 2005;115:986-95.
83. Duley L, Henderson-Smart DJ, Knight M, King JF. Antiplatelet agents for preventing preeclampsia and its complications. The Cochrane Database of systematic reviews 2003, Issue 4:CD004659.
84. Heyborne KD. Preeclampsia prevention: Lessons from the low-dose aspirin therapy trials. Am J Obstet Gynecol 2000;183:523-8.
85. Coomarasamy A, Honest H, Papaioannou S, Gee H, Khan KS. Aspirin for prevention of preeclampsia in women with historical risk factors: A systematic review. Obstet Gynecol 2003;101:1319-32.
86. Coomarasamy A, Braunholtz D, Song F, Taylor R, Khan KS. Individualizing use of aspirin to prevent preeclampsia: a framework for clinical decision making. BJOG 2003;110:882-8.
87. Newnham JP, Godfrey M, walters BJN, Phillips J, Evans SF. Low dose aspirin for the treatment of fetal growth restriction: A randomized controlled trial. Aust NZ J Obstet Gynaecol 1995;35:4:370-4.
88. Low dose aspirin in prevention and treatment of intrauterine growth retardation and pregnancy induced hypertension. Italian study of aspirin in pregnancy. Lancet 1993;341:396-400.

89. CLASP: a randomized trial of low-dose aspirin for the prevention and treatment of preeclampsia among 9364 pregnant women. CLASP (Collaborative Low-dose Aspirin Study in Pregnancy) Collaborative group. Lancet 1994;343:619-29.
90. Papageorghiou AT, Roberts N. Uterine aretrey Doppler screening for adverse pregnancy outcomes. Curr Op Obstet Gynecol 2005;17:584-90.
91. Baschat AA. Pathophysiology of fetal growth restriction: implications for diagnosis and surveillance. Obstet Gynecol Surv 2004;59:617-27.
92. Bower SJ, Harrington KF, Schucter K, McGirr C, Campbell S. Prediction of preeclampsia by abnormal uterine Doppler ultrasound and modification by aspirin. BJOG 1996;103:625-9.
93. Vainio M, Kujansuu E, Iso-Mustajarvi M, Maenpaa J. Low dose acetylsalicylic acid in prevention of pregnancy-induced hypertension and intrauterine growth retardation in women with bilateral uterine artery notches. BJOG 2002; 109:161-7.
94. Ebrashy A, Ibrahim M, Marzook A, Yousef D. Usefulness of aspirin therapy in high-risk pregnant women with abnormal uterine artery Doppler ultrasound at 14-16 weeks pregnancy: randomized controlled clinical trial. Croat Med J 2005;46:821-5.
95. Morris JM, Fay RA, Ellwood D, Cook CM, Devonald KJ. A randomized controlled trial of aspirin in patients with abnormal uterine artery blood flow. Obstet Gynecol 1996;87:74-8.
96. Gofinet F, Aboulker D, Paris-Llado J, et al. Screening with a uterine Doppler in low risk pregnant women followed by low dose aspirin in women with abnormal results: a multicenter randomized controlled trial. BJOG 2001;108:510-8.
97. Subtil D, Goeusse P, Houfflin-Debarge V, et al. Randomised comparison of uterine artery Doppler and aspirin (100 mg) with placebo in nulliparous women: the Essai Regional Aspirin Mere-Enfant study (Part 2). BJOG 2003;110:485-91.
98. Lan KKG and and DeMets DL. Discrete sequential boundaries for clinical trials. Biometrika 1983;70:659-63.
99. Demets DL and Lan KKG. Interim analysis – the Alpha-spending function approach. Stat Med 1994;13:1341-52.
100. Weinberg CR and Wilcox AJ. Reproductive epidemiology in modern epidemiology by Rothman KJ and Greenland S. 2nd edition. Lippincott-Raven, Philadelphia; 1998:585-608.
101. Ennis MG, Hinton D, Naylor M, Revow and Tibshirani R. A comparison of statistical learning methods on the GUSTO database. Statistics in Medicine 1998;17: 2501-8.
102. Eftekhar B, Mohammad K, Eftekhar Ardebili H, Ghodsi M and Ketabchi E. Comparison of artificial neural network and logistic regression models for prediction of mortality in head trauma based on initial clinical data. BMC Med Inform Decis Mak 2005;5:3.
103. Zhou XH, Obuchowski NA, and McClish DK. Statistical methods in diagnostic medicine. Wiley: New York; 2002.
104. Greiner M, Pfeiffer D and Smith RM. Principles and practical application of the receiver-operating characteristic analysis for diagnostic tests. Prev Vet Med 2000;45:23-41.
105. Fluss R, Faraggi D and Reiser B. Estimation of the Youden Index and its associated cut-off point. Biom J 2005;47:458-72.

106. Robins JM, Hernán MA, and Brumback B. Marginal structural models and causal inference in epidemiology. Epidemiology 2000;11:550-60.
107. Hernán MA, Brumback B and Robins JM. Marginal structural models to estimate the causal effect of zidovudine on the survival of HIV-positive men. Epidemiology 2000;11(5):561-70.
108. Hernán MA, Brumback B, and Robins JM. Marginal structural models to estimate the joint causal effect of nonrandomized treatments. J Am Statist Asoc 2001;96:440-8.
109. Wang C, Vlahov D, Galai N et al. The effect of HIV infection on overdose mortality. AIDS 2005;10;19(9):935-42.
110. Cole SR, Hernán MA, Robins JM et al. Effect of highly active antiretroviral therapy on time to acquired immunodeficiency syndrome or death using marginal structural models. Am J Epidemiol 2003;158:687-94.
111. Pearl J. Causality: models, reasoning, and inference. New York: Cambridge University Press; 2000.
112. Hogan JW and Lancaster T. Instrumental variables and inverse probability weighting for causal inference from longitudinal observational studies. Stat Methods Med Res 2004;13:17-48.
113. Lallas CL, Preminger M, Pearle MS et al. Internet based multi-institutional clinical research: A convenient and secure option. J Urol 2004;171:1880-5.
114. Kuchenbecker J, Burkhard HD, Schmitz K and Behrens-Braumann W. Use of internet technologies for data aquisition in large clinical trials. Telemedicine J and E-Health 2001;7(1):73-6.
115. Curley RM, Evans RL, Kaylor J, Pogask RM and Chinchilli VM. Development and deployment of an internet-based management system for use by the Asthma Clinical Research Network. Control Clin Trials 2001;22:135S-155S.
116. Lopez-Carrero C, Arriaza E, Bolanos E et al. Internet in clinical research based on a pilot experience. Contemp Clin Trials 2005;26:234-43.

K. Appendices – Included as a separate document

Appendix 1: Screening Questionnaire and Detailed Eligibility Questionnaire

Appendix 2: Baseline Questionnaires
- Personal Information Questionnaire
- Demographic Questionnaire
- Health and Reproductive History Questionnaire Part A and Part B
- Family Medical History Questionnaire
- Exercise Questionnaire
- Lifestyle Questionnaire
- Occupation Questionnaire
- Physical Measurements Form

Appendix 3: Previous Event Verification Form

Appendix 4: Active Phase and Pregnancy Diaries

Appendix 5: Safety Interview and Adherence Questionnaire

Appendix 6: Passive Follow-up Questionnaire

Appendix 7: Pregnancy Follow-up Questionnaire and Short Physical Measurement Form

Appendix 8: Recruitment Materials

Effects of ***A***spirin in ***Ge***station and ***R***eproduction
Study Protocol
Version 2.3: 03/17/08

The EAGeR Study is a randomized clinical trial with study centers at the University at Buffalo and the University of Utah and a Data Coordinating Center at the University of Haifa. The study is funded and organized by the National Institute of Child Health and Human Development, with Enrique F. Schisterman, PhD as the Project Officer.

For more information, go to the EAGeR study website:
http://www.EAGeRtrial.org

University at Buffalo Study Center
University at Buffalo—EAGeR Trial
Department of Social and Preventive Medicine
65 Farber Hall
Buffalo, NY 14214
(716) 829-3128
Principal Investigator: Jean Wactawski-Wende, PhD

University of Utah Study Center
University of Utah—EAGeR Trial
Department of Obstetrics and Gynecology
30 North 1900 East, 2B200
Salt Lake City, Utah 84132
(801) 581-4128
Principal Investigator: Robert Silver, MD

University of Haifa Data Coordinating Center
University of Haifa—EAGeR Trial
Department of Statistics
Mount Carmel
Haifa, Israel 31905
Principal Investigator: Noya Galai, PhD

Effects of Aspirin in Gestation and Reproduction (EAGeR)

Study Protocol

Table of Contents **Page**

Section	Page
A. Study Description and Objectives	4
B. Background	5
B.1. Blood Flow and Reproduction	5
B.2. Aspirin and Reproduction	7
B.3. Aspirin and Fertility	9
B.4. Aspirin and Pregnancy Loss	10
B.5. Aspirin and Obstetric Complications	11
B.6. Remaining Questions	13
C. Study Design	14
C.1. Introduction	14
C.2. Eligibility criteria	14
C.3. Treatment groups	16
C.4. Outcome measures	17
D. Participant Management	23
D.1. Recruitment	23
D.2. Study visits and contacts overview	23
D.3. Screening Process	24
D.4. Baseline study visit	25
D.5. Randomization	26
D.6. Active follow-up	28
D.7. Passive follow-up	31
D.8. Pregnancy follow-up	33
D.9. Specimen collection	37
D.10 Retention Plan and Compensation	38
D.11 Sub-study	39
E. Study Timeline	2
F. Study Administration	43
G. Statistical Analysis	44
G.1 Overview	44
G.2 Study endpoint and intention to treat analysis	44
G.3. Interim Analysis	45
G.4 Prediction models and secondary analyses	48
G.5 Evaluation of drop outs and compliance	49

G.6 Analysis for the sub-study on compliance	50
G.7 Implementation of statistical analysis	50
H. Data Management Procedures	51
H.1 Internet-based clinical trials	51
H.2 EAGeR web-based system	51
H.3 Questionnaires and other data forms	52
I. Human Subject Protection and Confidentiality Procedures	57
I.1 Risks and benefits	57
I.2 Protection of human subjects from research risks – data coordinating center responsibilities	59
I.3. Subject population	60
I.4 Confidentiality procedures	62
J. References	64
K. Appendices (in a separate document)	71

Figures:

Figure 1. Participants' management in the EAGeR Trial	24
Figure 2. Timeline for participant follow-up during attempt to conceive	33
Figure 3 Timeline for participant follow-up during pregnancy	36
Figure 4. Probability of live birth versus implantation probability per menstrual cycle	46

Tables:

Table 1. Activities performed during pregnancy follow-up	36
Table 2. Study phases and activities by months from start of funding	42
**Table 3.** Power for comparing live birth rates in treatment and placebo groups with no interim looks.	46
**Table 4.** Power for one or two interim looks assuming	47

A. STUDY DESCRIPTION AND OBJECTIVES

Human reproduction is a remarkable but inefficient process. Maximally fertile couples have only an approximately 30 percent chance of conception in a given menstrual cycle, and average monthly fecundity is 20%. Successful conception does not guarantee a live birth. Estimates of miscarriages and early pregnancy loss vary and range between about 15 to 31%. Available data suggest that low dose aspirin (LDA) has the potential to favorably impact on several aspects of reproduction including conception, implantation, early pregnancy loss, late fetal death, preeclampsia, small for gestational age fetus, placental insufficiency and preterm birth. LDA is an ideal therapy; it is apparently safe, widely available, has few maternal side effects, and is inexpensive. However, much remains to be learned. It is unclear which patients would benefit from LDA, what the optimal dose and duration of treatment should be, whether mechanisms other than an influence on blood flow contribute to the effects of LDA, and what the adverse effects of different doses and durations of treatment might be. Prior studies have focused on narrow aspects of the effect of LDA such as IVF outcome or the development of preeclampsia. Reproduction is unique in that each stage is inextricably linked. Better blood flow at the time of conception and implantation may ultimately lead to improved placental function, and a reduced risk of preeclampsia, SGA, and preterm birth.

Unanswered questions about LDA and pregnancy can best be answered through a comprehensive, carefully designed, prospective randomized clinical trial. Observational epidemiological studies of endpoints such as these are often limited in their ability to properly evaluate implantation and early pregnancy loss as this requires a very careful daily monitoring of hormonal levels based on collection of daily samples of first morning urine, necessitating both a very committed population and a well designed system to help women to comply with this demanding regimen. As such, we propose a prospective randomized controlled trial to evaluate the "Effects of Aspirin in Gestation and Reproduction (EAGeR)," with the following objectives:

Study Objectives

The **primary objective** of the study is:

To determine the effect of low-dose aspirin (LDA) in combination with folic acid on the incidence of live births relative to placebo plus folic acid.

Secondary objectives will include:

a To determine the effect of LDA in combination with folic acid on the incidence of hCG detected pregnancy relative to placebo plus folic acid.

b To determine the effect of LDA in combination with folic acid on the incidence of clinically recognized pregnancy (by 6.5 week ultrasound) relative to placebo plus folic acid.

c To determine the effect of LDA in combination with folic acid on specific pregnancy outcomes (gestational age, preterm birth, birthweight [small for gestational age], major neonatal complications, length of hospital stay for infant, preeclampsia, and cost) relative to placebo plus folic acid.

Additional objectives are:

d To evaluate the safety of LDA in the participants and fetuses.

e To create a repository of tissue samples for future evaluation.

B. BACKGROUND

Infertility is a prevalent problem which is getting much attention and is causing a growing burden on health services in the western world. Estimates of miscarriages and early pregnancy loss vary and range between about 15 to 31%. A prospective observational study conducted in China reported that 8% of all detectable conceptions, ended in clinical spontaneous abortion, and 25% in early pregnancy loss. Of the cycles with chemically (but not clinically) detected pregnancies 14% ended in early loss.[1]

Maximally fertile couples only have an approximately 30% chance of conception in any given menstrual cycle,[2] and average monthly fecundity is estimated at 20%.[3] Successful conception does not guarantee a live birth. Over 30% of conceptions are lost early in gestation.[4] Many of these are due to problems with implantation and may not be clinically apparent. Additionally, 12–15% of conceptions result in clinically recognized pregnancy loss.[5]

The causes of subfertility or infertility, implantation failure, miscarriage, fetal death, and pregnancy complications are myriad and often poorly understood. For example, pregnancy loss may be due to genetic abnormalities, uterine malformations, hormonal abnormalities, immunologic disorders, as well as other causes.[6] In many cases an etiology is never determined. Nonetheless, a unifying feature of some cases of infertility, pregnancy loss, and obstetric complications is a decrease in uterine, ovarian, and placental blood flow.

B.1. Blood Flow and Reproduction

a. Fertility

Impaired uterine blood flow has been suggested as a cause for infertility.[7] This is supported by the observation that embryo implantation failure is higher in women undergoing in vitro fertilization (IVF) who have impaired uterine perfusion as measured by Doppler ultrasound.[8-9] Also, a relatively thin endometrial lining has been associated with decreased likelihood of successful pregnancy.[10] Since endometrial growth requires blood flow for delivery of oxygen, hormones and nutrients,[11] suboptimal endometrial growth and development may be a direct consequence of inadequate uterine perfusion. In turn, poor endometrial growth decreases the odds of successful implantation and pregnancy outcome. It is likely that impaired ovarian perfusion may also contribute to the risk of sub- or infertility. Ovarian perfusion is critical to many mechanisms in the process of ovulation, which is a critical step for reproduction.

b. Pregnancy Loss

Abnormal placental blood flow also has been implicated as a cause of both early and late pregnancy loss. The majority of clinically recognized pregnancy losses are early,[12]

sporadic in nature, and due to genetic abnormalities.[13] However, it has been recognized for decades that recurrent pregnancy loss (usually defined as three or more losses with no more than one live birth[6] is associated with antiphospholipid syndrome (APS). APS is an autoimmune disorder characterized by the presence of specified levels of antiphospholipid antibodies (aPL) and one or more clinical features, including pregnancy loss, thrombosis, or autoimmune thrombocytopenia.[14] The histologic findings of placental infarction, necrosis, and vascular thrombosis in some cases of pregnancy loss associated with antiphospholipid antibodies[15-16] have led to the hypothesis that thrombosis in the uteroplacental circulation may lead to placental infarction and ultimately, pregnancy loss. Numerous studies have linked recurrent first trimester pregnancy loss with APS[14,17-18] and aPL are detected in approximately 10 – 15% of these women.[14,17-18]

Recurrent early pregnancy loss has been associated with heritable thrombophilias as well. These disorders typically involve deficiencies or abnormalities in anticoagulant proteins or an increase in pro-coagulant proteins, and have been associated with the risk of vascular thrombosis and pregnancy loss. Several case series and retrospective studies reported an association between the Factor V Leiden mutation (associated with abnormal factor V resistance to the anticoagulant effects of protein C), the G20210A mutation in the promoter of the prothrombin gene, and deficiencies of the anticoagulant proteins antithrombin III, protein C, and protein S, and pregnancy loss.[19]

In most studies, thrombophilias were more strongly associated with losses after 10 weeks of gestation as opposed to anembryonic or embryonic losses. A recent meta-analysis indicated an odds ratio of 2.0 for “early” and 7.8 for “late” recurrent pregnancy loss for women with the factor V Leiden mutation, and an odds ratio of 2.6 for “early” recurrent fetal loss in those with the prothrombin gene mutation.[20] APS was shown to be more strongly associated with fetal death as opposed to anembryonic or embryonic losses.[21] These observations are not surprising given the high frequency and myriad causes (mostly genetic) of early losses.

c. Obstetric Complications

Pregnancies resulting in live births also may be complicated by abnormal uterine and placental blood flow. Examples include preeclampsia, small for gestational age fetus (SGA), and placental insufficiency resulting in fetal hypoxia or acidemia. Women with APS have a markedly high risk for preeclampsia and SGA.[22] These conditions also are associated with heritable thrombophilias,[23] although findings among studies are inconsistent.[23-24]

Regardless of thrombophilia status, numerous studies demonstrate an association between decreased uterine artery blood flow and disorders associated with placental insufficiency.[25-27] A meta-analysis of 27 studies concluded that abnormal uterine artery Doppler studies increase the likelihood of developing preeclampsia six-fold, and the risk of SGA over three-fold.[28] It is noteworthy that results are inconsistent.[28] Also, histologic evidence of thrombosis and infarction are common features of preeclampsia and SGA,[29-30] suggesting a vascular etiology for these conditions.

Abnormal uterine blood flow may influence placental function in many ways. Decreased flow later in gestation leads to a reduction in oxygen and nutrients for the fetus with predictable adverse sequelae. Early in gestation, decreased perfusion may actually cause abnormal placental growth and development. Fisher and colleagues demonstrated that oxygen tension profoundly influences cytotrophoblast proliferation and invasion.[31] Inadequate maternal arterial blood flow may adversely affect the ability of cytotrophoblast to differentiate, inhibiting normal placental growth and development.[31] This may account for the shallow or absent invasion of uterine spiral arteries associated with preeclampsia.[32] Indeed, abnormal blood flow at the very beginning of pregnancy may harm placental development, leading to clinical consequences (e.g. the development of preeclampsia) that are not apparent until later in gestation.

Given the substantial impact abnormal vascular flow can have on ovulation, fertility, implantation, placental growth and development, miscarriage, fetal death, preeclampsia, fetal growth, and preterm birth, a medication that improves blood flow to reproductive organs and prevents placental thrombosis is extremely desirable. Ideally, the medication would be safe for mother and fetus both during conception and pregnancy, inexpensive, have few side-affects, and be widely available. Aspirin may be just such a medication.

B.2. Aspirin and Reproduction

Aspirin is best known among lay individuals for its analgesic properties. The drug has many other effects including anti-inflammatory and antipyretic actions. Aspirin also is a potent inhibitor of platelet aggregation, contributing to its anti-thrombotic effects. The primary biologic effects of aspirin are mediated by inhibition of the enzyme cyclo-oxygenase (Cox), also termed prostaglandin G/H synthase. There are at least two isoforms of Cox, Cox-1 and Cox-2, both of which are inhibited by aspirin.[33] These enzymes catalyze the conversion of arachidonic acid to eicosanoids, or lipid mediators. The end products of arachidonic metabolism are varied and influenced by local enzymes in specific cell types. Thromboxane (TXA_2) is the major Cox product in platelets and is prothrombotic since it promotes platelet aggregation and vasoconstriction. At high doses, aspirin inhibits prostacyclin formation (PGI_2) in endothelial cells. This effect also is prothrombotic since PGI_2 causes vasodilation and decreases platelet aggregation. However, at low doses (typically 70 – 150mg), aspirin effectively inhibits platelet production of TXA_2 with little effect on endothelial PGI_2.[34-35] This is primarily due to the first pass effect in the liver wherein circulating platelets are exposed to the aspirin but at low doses, the drug is metabolized before reaching vascular endothelium. Thus low dose aspirin (LDA), results in a net increase in the PGI_2:TXA_2 ratio, decreasing thrombosis and increasing blood flow.

The anti-inflammatory properties of aspirin also are desirable in the prevention and treatment of reproductive pathology. In part, the pathophysiology of some cases of infertility, implantation failure, idiopathic miscarriage, APS, and preeclampsia is immunologically mediated. Although success has been mixed, anti-inflammatory medications have been used to treat these conditions. Thus, aspirin may reduce the rate of obstetric complications by suppressing the immune response in addition to improving blood flow.

LDA is generally considered to be efficacious in the prevention of certain types of thrombosis such as ischemic events in patients with coronary artery disease.[36] The drug also may reduce the risk of thrombosis associated with essential thrombocythemia.[37-39]

LDA is extremely attractive as a therapy for reproductive disorders because of its' high safety profile. Randomized clinical trials in thousands of pregnant women showed no increase in adverse fetal sequelae in doses < 150 mg per day.[40-42] The safety of higher doses is less clear. Initial reports suggested that prolonged exposure to high doses of Cox inhibitors such as indomethacin or other non-steroidal anti-inflammatory drugs (NSAIDs) may cause adverse fetal effects. These include premature closure of the ductus arteriosus, pulmonary hypertension, renal damage, intraventricular hemmorhage, and necrotizing enterocolitis.[43-44] More recently, a meta-analysis concluded that indomethacin use was not associated with adverse fetal effects.[45] It is controversial as to whether aspirin use increases the risk for fetal malformations. Aspirin was not associated with an overall increase in the risk of congenital malformations in a meta-analysis of 22 studies.[46] The same meta-analysis reported a possible increase in the risk of gastroschisis (OR 2.37; 95% Confidence Interval (CI,) 1.44 – 3.88) in infants exposed to aspirin (325 mg per day) in the first trimester.[46] This is biologically plausible since this malformation may be caused by vascular disruption of mesenteric vessels.[47] Results were not confirmed in a recent population based case-control study[48] and the association between aspirin use and gastroschisis remains uncertain. One study indicated an increased risk of miscarriage in women taking aspirin or other NSAIDs during the prenatal period.[49] However, the study included few (53) women taking NSAIDs, did not include information regarding dose and duration of treatment, was subject to selection bias, and did not account for some confounding variables such as aspirin use in women undergoing assisted reproductive technology or those with APS. Indeed, aspirin is often used empirically to treat infertility, recurrent miscarriage and APS (see below). Studies regarding safety are difficult to compare due to different doses, duration, and timing (with regard to pregnancy) of aspirin use. Nonetheless, the majority of data indicates minimal fetal risk from *in utero* LDA exposure.

The discovery of two Cox isoforms led to hopes that selective inhibition of one isoform might allow for the beneficial effects of Cox inhibition, without the untoward effects. Cox-1 was thought to be constitutively expressed while Cox-2 was inducible in response to infection or inflammation, as well as normal labor.[50] The Utah group was one of the first to demonstrate this in gestational tissues in a murine model of infection-mediated fetal death.[51] It appeared as though inhibition of Cox-2 (expressed under abnormal conditions) provided the beneficial effects of NSAIDs while inhibition of Cox-1 (normally expressed) led to unwanted consequences, making it an attractive potential therapy for preterm labor.[50-51] Unfortunately, selective inhibition of Cox-2 may lead to the same untoward fetal effects observed after non-selective Cox inhibition.[52-53] Newer Cox-2 selective agents may yet prove to be effective and safe, but none are ready for general clinical use.[50]

B.3. Aspirin and Fertility

Interest in the potential therapeutic effects of LDA on human fecundity began with the demonstration of its beneficial use in women with APS. The aPL that characterize the syndrome have been linked to reproductive abnormalities such as recurrent pregnancy loss. Women with recurrent pregnancy loss in turn have been shown to develop a higher than expected incidence of unexplained infertility. Because abnormal implantation may be a common etiology for both pregnancy loss and unexplained infertility, aPL have been hypothesized to play a role in both conditions. This in part prompted the hypothesis that LDA may enhance fecundity. Aside from aPL, other potential pathologic mechanisms that may reduce fecundity but may be treated by LDA have been proposed including diminished uterine perfusion, decreased endometrial thickness, uterine/endometrial inflammation, abnormal folliculogenesis, diminished ovarian progesterone production, and luteinized unruptured follicle syndrome. Diminished uterine perfusion may be a root abnormality initiating each of the other hypothesized mechanisms. Each of these potential mechanisms is amenable to experimental verification, but studies to date have not proven efficacy.

a. Uterine Perfusion - It has been suggested that poor uterine perfusion is a proximate cause of infertility.[7] Indeed, LDA appears to improve uterine blood flow as measured by Doppler pulsatility index.[54-56] One postulated underlying therapeutic mechanism of LDA on uterine blood flow is the vasodilatory effect of an increased PGI_2:TXA_2 ratio.[55] Another possible benefit of LDA is the inhibition of platelet aggregation and thereby physiologic endometrial "hyperthrombosis".

Paradoxically, the prostaglandin inhibitory effect of aspirin mediated by the irreversible acetylation of Cox can compromise implantation in women.[57-58] The net reproductive effect of aspirin appears to depend upon a balance of complex interactions between such opposing mechanisms as vasodilation and prostaglandin inhibition and possibly other dose-dependent effects of aspirin on prostaglandin economy. Although increased uterine perfusion may benefit implantation, it does not appear that the effects of aspirin are mediated by altered circulating sex steroid concentrations or by increased endometrial thickness as measured by transvaginal ultrasonography.[55,59-60]

b. Antiphospholipid Antibodies (aPL) and Infertility - Levels of aPL have been reported to be higher in women with infertility than in fertile controls.[61-62] It is unclear how these autoantibodies interfere with fecundity. One possibility is altered reproductive cell activity secondary to antibody binding to cell surface antigens (possibly containing phospholipids). Gametes, zygote cells and trophoblast could all be targets. Cell activity alterations also could disrupt hemostatic mechanisms leading to clinically significant microthrombi at the tissue level.[63] In turn, this may diminish implantation and subsequent fecundity.

A meta-analysis of seven studies including women with aPL undergoing in vitro fertilization (IVF), but not specifically treated for the presence of aPL, did not show an impact of the antibodies on implantation or pregnancy rates.[64] However, studies of the association of aPL and fecundity have included such wide variation of study

populations, antibodies tested, and treatment regimens, that conclusions regarding an association of aPL and fecundity, and the efficacy of treatment remain controversial.

The only randomized placebo-controlled crossover trial performed to date did not show a benefit to treatment in the small subset of aPL positive women with previously documented IVF implantation failure.[63] Women were treated with heparin and LDA precluding assessment of the independent effect of aspirin. Because LDA was not started until the day of embryo transfer, the effect of LDA and heparin on endometrial development could not be ascertained.

c. Aspirin Therapy and Outcome of In Vitro Fertilization (IVF) - Contributing factors to low baseline human fecundity have not been fully characterized but decreased implantation appears to be a major component. The protean effects of aspirin include both potentially helpful and harmful consequences upon the process of implantation. Therefore aspirin has been employed experimentally in attempts to enhance implantation. IVF offers an opportunity to analyze sequential steps in the early reproductive processes.

Available studies using aspirin during IVF have studied heterogeneous populations. These have included women whose endometriums were thin, previous poor responders to follicle stimulating hormone (FSH) of ovarian stimulation, donor oocyte recipients, and women whose oocytes are undergoing intracytoplasmic sperm injection (ICSI). Some studies included unselected populations (all infertility diagnoses and combinations) undergoing IVF. Also, aspirin protocols in published IVF studies vary as to dose, duration and cycle starting point. Not surprisingly, the conclusions vary regarding the efficacy of aspirin for an infertile IVF population and mechanistic effects cannot be precisely defined.

A total of 2,500 IVF patients have been randomized to aspirin versus placebo or no treatment in seven trials. The largest study (N = 1380) used unselected IVF patients and reported a statistically significant benefit for the use of LDA.[65] The possibility remains that routine use of aspirin for IVF (as is currently being done in many reproductive centers) may confer benefit. Fortunately, no serious side effects of aspirin use for IVF have been reported.

B.4. Aspirin and Pregnancy Loss

LDA was first used to treat recurrent pregnancy loss in women with APS. Use of the drug was prompted by recognition that thrombosis was central to the pathophysiology of APS, as well as the observation that IgG fraction from women with APS increases placental production of TXA_2 in humans.[66] Aspirin reduced TXA_2 under similar conditions without affecting PGI_2, thus decreasing the TXA_2:PGI_2 ratio.[67] It is somewhat difficult to assess the independent effect of aspirin in women with APS since most have been treated with additional agents such as heparins and or prednisone. Systematic reviews[68-69] of interventions intended to improve obstetric outcome in women with aPL identified three trials comparing aspirin alone to no treatment.[70-72] Aspirin alone had a relative risk of 1.05 (95% CI, 0.66, 1.68) for live birth compared to no treatment.[68-69] It is noteworthy that these trials were very small, included many women who do not meet

criteria for APS (either low titers of antibody or too few losses or medical problems), and were not all placebo controlled.[70-72] The authors conclude that a small benefit of aspirin alone cannot be rejected on the basis of available studies.[68] Unfractionated heparin combined with aspirin showed a relative risk of 0.46 (95% CI, 0.29 – 0.71) for pregnancy loss in two trials including 140 women with recurrent pregnancy loss and APS compared to aspirin alone.[68-69] A third trial showed no difference between low molecular weight heparin and aspirin compared to aspirin alone.[73] Both groups in the latter trial had excellent outcomes.

LDA also has been used to treat recurrent pregnancy loss in women without aPL. Most trials used heparin as well as aspirin, making it difficult to determine the independent effect of aspirin. A systematic review of anticoagulants for the treatment of women with recurrent pregnancy loss (defined as two early losses or one unexplained fetal death) identified two controlled trials.[74] One included 54 women and compared LDA to placebo where LDA had a relative risk for live birth of 1.00 (95% CI, 0.78 – 1.56).[72] The other included women with heritable thrombophilias and fetal death.[75] Although heparin improved the odds of live birth in this cohort compared to women treated with LDA,[75] the presence of a heritable thrombophilia may introduce a substantial bias in the findings.

The gestational age of prior losses may influence whether or not LDA is effective in improving subsequent pregnancy outcome. Rai and colleagues assessed the effect of LDA on pregnancy outcome in women with recurrent pregnancy loss.[76] The study was retrospective and not randomized, although the group was large (805 women with early miscarriage and 250 with late losses). LDA did not have a statistically significant increase in the live birth rate in women with early losses (OR 1.24, 95% CI, 0.93-1.67). In contrast, in women with unexplained fetal death, the OR for live birth associated with LDA was 1.88 (95% CI, 1.04-3.37).[76] The Utah group had similar findings in an uncontrolled retrospective cohort of 230 women with unexplained fetal death.[77] In univariate analysis, LDA was associated with an OR of 0.41 (95%CI 0.25-0.68) for subsequent pregnancy loss. After controlling for confounders in a multivariate analysis, LDA was associated with an OR of 0.12 (95% CI 0.05-0.32) in women 35 years of age or older.[77] Thus, LDA appears to be more helpful in women with prior fetal death as opposed to recurrent first trimester losses. We speculate that LDA may improve the utero-placental circulation, decreasing the risk of placental thrombosis, infarction, and insufficiency which have been associated with fetal death.[77-78] On the other hand, anembryonic and embryonic losses are common in normal individuals and often are due to *de novo* non-dysjunctional aneuploidy. Accordingly, aspirin may be less likely to help patients with recurrent early pregnancy loss. There is still a potential role for LDA in the treatment of some cases of early pregnancy failure. Aspirin (as well as heparin) modulates trophoblast apoptosis in BeWo cells and placental explants in vitro, providing another mechanism for aspirin to improve pregnancy outcome.[79]

B.5. Aspirin and Obstetric Complications

The best studied use of LDA in reproduction is the prevention of preeclampsia. As with APS, there are several reports indicating an increase in the TXA_2:PGI_2 ratio (metabolites) in serum in women with preeclampsia compared to normotensive controls.[80-81] Vainio and colleagues demonstrated an improvement in this ratio in women

treated with LDA.[81] Recent data from a murine model also support this paradigm.[82] Depression of platelet TXA_2 while preserving reproductive function (intended to mimic the effects of LDA) decreased platelet aggregation and prevented thrombosis.

Many large randomized controlled trials have been conducted in populations with both high and low risk for the development of preeclampsia. Duley et al performed a systematic review that included 51 trials involving 36,500 women treated with antiplatelet agents for the prevention of preeclampsia.[83] Forty-four of the trials involved the use of aspirin alone (compared with placebo or no treatment) while the remainder included other treatments, often in conjunction with aspirin. Overall, use of antiplatelet agents conferred a 19% reduction in the risk of preeclampsia (RR 0.81; 95% CI, 0.74-0.96). LDA was associated with a risk reduction for the development of preeclampsia associated with proteinuria, regardless of whether women were at moderate or high risk for preeclampsia, gestational age at trial entry, or dose of LDA.[83] There was a greater risk reduction in women treated with doses greater than 75 mg/day (RR 0.49, 95% CI 0.38-0.65) compared to lower doses (RR 0.86, 95% CI 0.79-0.93). Others reviews emphasize the increased benefit from LDA in women with historical risk factors (high risk for preeclampsia) and suggest that focusing on at risk groups would decrease the number of women it is necessary to treat to prevent a single case of preeclampsia.[84-86] Of course, the “risk:benefit” ratio also is influenced by risk, which appears to be quite low for LDA. Thus, treatment of low risk women may yet prove to be justified.

LDA also may reduce the risk of other adverse perinatal outcomes such as SGA fetus and late fetal death. In 32 trials of 24,310 women, antiplatelet therapy conferred an 8% reduction in SGA (RR 0.92, 95% CI 0.85-1.00) in women treated with the intent to prevent preeclampsia.[83] A small trial showed no benefit from LDA in women treated after the diagnosis of SGA fetus.[87] LDA also has not been effective when started after the diagnosis of preeclampsia.[88-89] The review by Duley and colleagues noted a 16% reduction in combined fetal, neonatal, and infant mortality in women taking antiplatelet therapy (RR 0.84, 95% CI 0.74-0.96). Perinatal death and SGA fetuses were not primary end points of these trials and results should be interpreted with caution.

Doppler velocimetry of the uterine, umbilical, and fetal arteries have been used to estimate flow and vascular resistance during pregnancy.[90-91] Although the positive predictive value is poor, especially in low risk populations, there is a recognized increase in subsequent risk of preeclampsia, SGA, and fetal death in women with abnormal uterine artery Doppler studies in the second trimester.[28] Several investigators have treated women with abnormal uterine artery Doppler studies with LDA in attempt to improve perinatal outcome. In women at high risk for the development of preeclampsia and SGA with abnormal uterine artery Doppler studies, LDA can reduce the rate of subsequent preeclampsia.[92-94] In contrast, LDA did not improve outcome in most investigations conducted in low risk populations with abnormal Doppler studies.[95-97]

Prevention of preterm birth is a very important potential benefit of LDA since prematurity is a major cause of perinatal morbidity and mortality. The drug may decrease the risk of preterm birth indirectly by reducing the risk of preeclampsia, SGA, and placental insufficiency. These conditions often result in iatrogenic preterm birth in an attempt to improve perinatal outcome. LDA also may have a direct effect on preterm labor and

delivery since Cox inhibition decreases uterine contractility and high dose aspirin use has been associated with delayed onset of labor. In the systematic review by Duley et al., data were available regarding preterm birth from 28 studies including over 30,000 women. The authors noted a 7% reduction in delivery < 37 weeks gestation in women treated with antiplatelet agents (RR 0.93, 95% CI 0.89-0.98). These findings are intriguing because even a small reduction in the rate of preterm birth would have dramatic effects on perinatal morbidity and health care costs.

B.6. Remaining Questions

Available data suggest that LDA has the potential to favorably impact on several aspects of reproduction including conception, implantation, early pregnancy loss, late fetal death, preeclampsia, SGA fetus, placental insufficiency and preterm birth. LDA is an ideal therapy; it is apparently safe, widely available, has few maternal side effects, and is inexpensive. The possibility that such a drug could have a major impact on obstetric outcome is extremely attractive. It is unclear which patients would benefit from LDA. Prior studies have focused on narrow aspects of the effect of LDA such as IVF outcome or the development of preeclampsia. Reproduction is unique in that the different stages are inextricably linked. Better blood flow at the time of conception and implantation may ultimately lead to improved placental function, and a reduced risk of preeclampsia, SGA, and preterm birth. Unanswered questions about LDA and pregnancy can only be answered through a comprehensive, carefully designed, prospective randomized clinical trial, such as that proposed in the EAGeR trial.

C. STUDY DESIGN

C.1. Introduction

The EAGeR study is a two-site prospective randomized double-blind placebo-controlled clinical trial evaluating the effect of low-dose aspirin use on gestation and reproduction outcomes. The target population is women aged 18-40 who have had one or two pregnancy losses in the past and who are actively trying to conceive. Women shall be screened at enrollment to ensure they are not pregnant and eligible with the goal of recruiting and randomizing 1600 completed subjects into two clinical sites. Subjects shall be randomly assigned to receive low dose aspirin (81 mg) plus folic acid (400 mcg) or identical looking placebo plus folic acid. These enrollees will be actively followed for two menstrual cycles or until becoming pregnant, whichever comes first. Active follow-up entails collection of questionnaire data as well as regular home specimen collection. Women not becoming pregnant within the first two cycles shall remain in the study under passive follow-up for an additional four menstrual cycles or until becoming pregnant, whichever comes first. Passive follow-up entails intake of Study Pills and folic acid and use of fertility monitors, pregnancy testing, and follow up questionnaires, but no home specimen collection. Women who become pregnant shall be followed throughout gestation for pregnancy outcomes and will continue to take Study Pills and folic acid throughout pregnancy until their 36th gestational week.

The trial will be conducted at two clinical sites, the University of Utah in Salt Lake City, Utah and the University at Buffalo in Buffalo, New York. The Utah site includes four medical centers and the Buffalo site will be conducted at a free standing research clinic at the University enrolling participants from the western NY region. Henceforth the term “clinical center” refers to each of the five clinics participating in the trial at the two sites. The study will take a total of 5 years to complete including start up activities (Phase I), recruitment and follow-up of pregnancy outcomes in all subjects (Phase II) and wrap-up and analysis (Phase III). Participants will be recruited from throughout the community and enrolled over approximately a three to four year time period. By the end of the study, it is expected the University of Utah will have enrolled 1100 complete subjects, two thirds of the study population, and the University at Buffalo will have enrolled 500 complete subjects. **To be considered a complete subject, participants need to complete at least 90% of their scheduled clinic visits.** The number of required visits will depend on the participant’s length of follow-up in the trial.

C.2. Eligibility Criteria

The target population is women of childbearing age who experienced one or two spontaneous pregnancy losses in the past and are actively trying to get pregnant. Women must meet the following eligibility criteria to be considered for participation in the EAGeR trial:

Inclusion Criteria:

a Women experiencing one or two pregnancy losses at any point in gestation in the past that were not elective termination(s). At least one of these losses must be well documented by one of the following:

- Sonogram demonstrating anembryonic loss, embryonic loss or fetal death.
- Histologic confirmation of products of conception that were spontaneously passed per vagina or surgically obtained.
- Hospital records of fetus delivery
- Late menses and positive serum hCG or positive urine hCG documented by hospital or clinic records followed by either a negative hospital/clinic pregnancy test or a decline in urinary hCG level over 3 days.*

b No more than 5 pregnancies in total including the pregnancy loss(es)**.

c Up to two prior pregnancies that did not end in a loss **

d Presence of intact tubes (both), ovaries (both), and uterus.

e Between 18 and 40 years of age at time of baseline visit

f Regular menstrual periods between 21 – 42 days in length (within the last 12 months). Regular menstrual periods are defined as no more than an 8-day difference between the woman's shortest and longest cycle.

g No more than one missed menses in the past 12 months (other than those missed due to pregnancy or breastfeeding).

h Actively trying to conceive with a male partner and not using contraception by the baseline visit.

i Not currently pregnant at the baseline or randomization visits.

j Ability and willingness to give informed consent.

k Willingness to be randomized and to take daily study pills for 6 months to a possible 15 months.

* Home pregnancy tests without hCG confirmation from a healthcare provider (either serum or urine) will not be accepted.

** Women may have up to two pregnancies beyond 20 weeks that were not losses, two spontaneous pregnancy losses at any time in the past, and up to one therapeutic or elective termination (two therapeutic or elective terminations if no other pregnancies). Ectopic and molar pregnancies would, for the purpose of enrollment, be considered in the same category as therapeutic termination pregnancies. Women with more than two live births or those with more than two losses, regardless of the week of gestation of the loss, are excluded.

Exclusion Criteria:

a Known allergies to aspirin or non-steroidal anti-inflammatory agents.

b Clinical indication for anticoagulant therapy. These include prior or current thrombosis, antiphospholipid syndrome (APS) or known major thrombophilia.

c Clinical indication for chronic use of NSAIDs such as rheumatoid arthritis.

d Indication for additional folic acid supplementation, such as prior infant with neural tube defect (NTD), seizure disorder.

e Medical contraindication to aspirin therapy. These include uncontrolled asthma, nasal polyps, bleeding disorders, or history of gastrointestinal ulcer.

f Presence of major medical disorders (regardless of severity). These include diabetes, hypertension, systemic lupus erythematosus (SLE), untreated or active cancer (any cancer in remission or non-melanoma skin cancer is not included in the exclusion criteria), liver disease, renal disease, rheumatoid arthritis, cardiac disease, pulmonary disease other than mild asthma, neurologic disease requiring medical treatment, uncontrolled hypothyroidism, uncontrolled seizure disorder. Untreated vitamin B_{12} deficiency, severe anemia (Hct < 30%), hemophilia, gout, nasal polyps, among others.

g Currently undergoing/planned use of assisted reproductive techniques during trial (IVF; IUI; Clomid)

h History of infertility or sub-fertility. This includes any of the following:

- No conception after ≥ 1 year of unprotected intercourse and actively trying to conceive.
- Any prior medical treatment for infertility.
- Prior treatment for known pelvic inflammatory disease.
- Known male infertility or sperm abnormality (current partner)
- Known tubal occlusion, anovulation, uterine abnormality, or endometriosis stage III or IV.
- History of polycystic ovarian syndrome.

i Presence of unstable mental disorder. These include bipolar illness, schizophrenia, uncontrolled depression, uncontrolled anxiety disorder.

j Known current or recent alcohol abuse or illicit drug use.

k Current diagnosis of sexually transmitted infection (STI) (temporary exclusion)

C.3. Treatment groups

The trial aims to evaluate the effects of low-dose aspirin on gestation and reproductive outcomes. In addition to the LDA, all participants will be provided a folic acid supplement to be taken daily, which is currently routinely recommended for women attempting to become pregnant.

Participants will be randomized into two groups receiving one of the following:

- Aspirin 81 mg taken orally daily plus folic acid 400 µg taken orally daily
- Placebo plus folic acid 400 µg taken orally daily

Aspirin and placebo capsules will be identical in look and weight to maintain blinding. The duration of treatment regimens will be up to 6 menstrual cycles, with those who become pregnant continuing until week 36 of pregnancy.

For the remainder of the protocol, the term "Study Pills" will be used to refer to aspirin and placebo to differentiate it from folic acid.

C.4. Outcome Measures

a hCG recognized pregnancy (implantation)

Home pregnancy kits (Quidel Quickview) will be used to identify pregnancies at the time of implantation. A positive qualitative result on a home pregnancy test followed by confirmation of quantitative hCG levels ≥ 0.025 ng/mL in urine specimens will define hCG pregnancy. Positive spot urine test at clinic visits will be also considered as hCG pregnancy. Quantitative hCG analysis will be performed from batched urine samples sent to the NIH repository.

b Clinically recognized pregnancy

Clinical recognition of pregnancy will be determined by

1) Documentation of the gestational sac from an ultrasound scan(s) at 6.5 weeks or later (or fetal heart tones with histologic confirmation of gestational tissue). We will utilize physicians' reports from routinely scheduled prenatal care as well as the study ultrasound performed early in pregnancy.

OR

2) Positive serum hCG test and missed menses

c Early pregnancy loss (EPL)

Pregnancy loss is a measure of impaired human fecundity and encompasses two types of losses: 1) early pregnancy loss or hCG detected pregnancy loss and 2) clinically recognized pregnancy loss. It is estimated that approximately one-third of all pregnancies are spontaneously aborted of which 2/3 are hCG pregnancy losses.

- hCG pregnancy loss

  An hCG pregnancy loss will be defined as a urinary hCG level ≥ 0.025 ng/mL for at least 3 consecutive days followed by a decline. The rise and fall of hCG will be evaluated during the 10 days prior to the next menstrual period through day 5 of the next menstrual period. Quantitative hCG analysis will be performed from batched urine samples sent to the NIH repository.

- Clinically recognized spontaneous abortion

A clinically recognized spontaneous abortion will be defined as that detected by the woman or her doctor before 20 weeks completed gestation and having had a confirmed pregnancy by documentation of the gestational sac or heart tones (see above).

d Pregnancy losses occurring less than 10 weeks may be anembryonic, embryonic, or unknown

- Anembryonic pregnancy loss

  An anembryonic pregnancy is a gestation in which embryonic development does not occur. Sonographic criteria include:

  1. Mean gestational sac diameter of ≥ 16 mm without an embryo
  2. Mean gestational sac diameter of ≥ 8 mm without a yolk sac
  3. No visible embryo two weeks after detection of gestational sac of any size.
  4. Positive serum hCG with missed menses and no sac visible on sonogram.

- Embryonic pregnancy loss

  Pregnancy loss occurring before 9 weeks completed gestation. Sonographic criteria include:

  1. Embryo with crown rump length ≤ 30 mm with no cardiac activity.

e Fetal pregnancy loss

Pregnancy loss occurring at 10 to 19 completed weeks gestation. Death of a fetus with crown rump length of > 30 mm and composite mean gestational age (based on biparietal diameter, abdominal circumference and femur length) < 20 weeks completed gestation.

f Stillbirth

Pregnancy loss occurring AT OR after 20 completed weeks gestation. The complete expulsion or extraction from its mother, after at least 20 completed weeks pregnancy, of a product of conception in which, after such expulsion or extraction, there is no breathing, beating of the heart, pulsation of the umbilical cord, or unmistakable movement of voluntary muscle.

h. Ectopic Pregnancy

A pregnancy that has implanted somewhere other than the uterus as confirmed by sonography, laparoscopy or laparotomy.

i. Molar Pregnancy (determined by pathology)

1. Complete hydatidiform mole: Hydropic degeneration and swelling of villous stroma, absence of blood vessels in the swollen villi, proliferation of trophoblastic endothelium, and absence of a fetus and amnion.
2. Partial hydatidiform mole: Hydropic degeneration and swelling of some (usually avascular) villi, with sparing of other villi. Focal trophoblastic hyperplasia. The fetus usually has features of triploidy and the karyotype is usually triploid.

j. Live birth

Birth of a fetus with any sign of life (e.g. pulsation of umbilical cord, purposeful movements)

k. Preeclampsia

The following definitions for preeclampsia will be used as recorded in participants' medical charts during pregnancy and obtained with chart abstraction.

Mild pregnancy related hypertension is defined as a systolic blood pressure ≥ 140 or diastolic blood pressure ≥90 on two occasions 4-240 hours (10 days) apart on or after 20 weeks 0 days of gestation. The first of the two blood pressures must have been obtained prior to 24 hours postpartum. All blood pressures obtained during hospital admissions will be counted, except intra-operative blood pressures and systolic blood pressures during labor.

Normotensive: A woman is considered normotensive if she was not previously diagnosed as being hypertensive. A women is considered to be previously hypertensive if she reports a physician documented history of elevated blood pressure outside of pregnancy or she manifests elevated blood pressure (systolic >140 mmHg or diastolic >90 mmHg, documented by physician) any time between conception and 140 days of gestation.

Note that the screening for EAGeR trial excludes women with known history of hypertension and those treated with anti-hypertensive drugs.

Proteinuria is defined by any of the following criteria
1) A total protein excretion value of ≥ 300 mg in a 24-hour urine
2) The finding of ≥ 2+ on dipstick or a protein creatinine ratio ≥ 0.35 if a 24-hour urine is not available.
3) If a 24-hour urine is not available, the finding of two or more urine dip sticks value of ≥ 1+ in the absence of a urinary tract infection (defined by a positive culture or nitrite dipstick that was regarded by the managing clinician as representing infection). The two values must be obtained from 4-240 hours of each other.

The 24-hour urine is the definitive test for proteinuria and supersedes all previous dipstick values. Thus if two dipstick readings show ≥ 2+ but the follow-up 24-hour urine collections shows protein < 300 mg, the patient is not considered to have proteinuria.

Mild preeclampsia is defined as mild pregnancy related hypertension and proteinuria, as defined above, if at least one elevated blood pressure measurements (systolic >140 mmHg or diastolic >90 mmHg) is recorded within 72 hours of diagnosis of proteinuria.

Severe preeclampia

Several criteria are used:

1) Severe pregnancy related hypertension plus proteinuria. Severe pregnancy-related is defined as a systolic blood pressure ≥ 160 or diastolic blood pressure ≥110 on two occasions 4-240 hours (10 days) apart on or after 20 weeks 0 days of gestation in a previously normotensive woman. The first of the two blood pressures must have been obtained prior to 24 hours postpartum. All blood pressures obtained during hospital admissions will be counted, except intra-operative blood pressures and systolic blood pressures during labor. A single systolic blood pressure ≥ 160 mmHg or diastolic blood pressure ≥ 110 mmHg qualifies for this diagnosis if the participant was treated with anti-hypertensive medication.
2) Mild preeclampia with severe proteinuria (24-hour urine collection ≥ 5 grams protein)
3) Mild pregnancy related hypertension and oliguria (24-hour urine collection < 500 ml volume)
4) Mild pregnancy related hypertension and pulmonary edema (confirmed by chest x-ray)
5) Mild pregnancy related hypertension and thrombocytopenia (a platelet count < 100,000/mm3)

HELLP Syndrome

Pregnancy related hypertension (mild or severe) with all of the following occurring within 72 hours of the elevated blood pressure measurement:
a. Thrombocytopenia: platelet count < 100,000/mm^3
b. SGOT (AST) ≥ 100 U/L
c. Hemolysis: either LDH ≥ 600 U/L or total bilirubin ≥ 1.2 mg/dl or peripheral blood smear showing nucleated RBCs, schistocytes, or an elevated reticulocyte count

Eclampsia

Pregnancy related hypertension (mild or severe) and a seizure during pregnancy

Hierarchy of conditions severity

If a participant satisfies the criteria for more than one of the categories (for example, if she satisfies the criteria for mild preeclampsia but also has a platelet

count less than 100,000/mm3) she will be diagnosed in the highest numbered category (arbitrarily considering severity to be in the order – mild preeclampsia, severe preeclampsia, HELLP, eclampsia).

l. Small for gestational age infant

Infant birthweight is ≤ 10% for gestational age (weight < 10th percentile), as defined by the standards of Kramer, et al .

(**http://www.pediatrics.org/cgi/content/full/108/2/e35**).

m. Preterm birth

Delivery prior to 37 weeks of completed gestation. Spontaneous preterm labor and delivery and preterm premature rupture of membranes will be distinguished from indicated preterm birth due to for example preeclampsia or SGA fetus.

n. Abnormal fetal testing

Non-reactive non-stress test (NST), positive contraction stress test (CST), abnormal biophysical profile (BBP ≤ 6).

o. Fetal intolerance of labor

Cesarean delivery due to abnormal fetal heart rate tracing.

p. Abruption or vaginal bleeding

Abruption or vaginal bleeding that results in hospitalization, Cesarean delivery, or pre-term birth.

q. Length of hospital stay for the infant.

r. Cost: Cost data collected will include charges (rather than actual cost) for maternal and neonatal inpatient care. Data will be collected from the hospital of delivery for live births. Data regarding charges will be obtained through hospital administrative electronic records. Additional data regarding this endpoint will include total length of stay (maternal and neonatal) as well as ICU admission. These data will be obtained from the medical record.

For the above outcomes, gestational age will be determined by the following:

1) Gestational age will be based on the 6.5 week study sonogram finding.
2) For participants with a pregnancy loss before the 6.5 week ultrasound, gestational age will be defined as the number of days from ovulation to the loss and then add 14 days onto that.
3) If the 6.5 week study ultrasound is not available and the pregnancy loss is believed to be after the point when the 6.5 week ultrasound should have been done, use the earliest sonogram obtained clinically in pregnancy and compare it to the last menstrual period (LMP). If within the following limits, use the LMP as the gestational age. If not, then use the sonogram results.

Gestational age according to first ultrasound	Limit of agreement to accept LMP
Up to 19 weeks, 6 days	±7 days
20 weeks, 0 days to 29 weeks, 6 days	±14 days
30 weeks, 0 days or greater	±21 days

If there are no available sonograms but the date of ovulation is available, then gestational age will be defined as the days from ovulation to delivery, plus 14.

If there are no available sonograms and date of ovulation is not available, the LMP will be used for the gestational age (ie delivery date-lmp date)

D. PARTICIPANT MANAGEMENT

D.1. Recruitment

The recruitment plan for the EAGeR trial will be site-specific.

Recruitment plan summary

Potential study participants will be recruited from multiple sources so as to reach a diverse study population that includes women who do and do not routinely access the health care system. Two main recruitment methods will be employed to reach potential subjects: **clinic-based recruitment** and **community-based recruitment**. Recruitment methods will include attempts to enroll women of various racial and socioeconomic backgrounds.

The main recruitment strategy of this trial will be based on the clinical settings, where women who have recently had a pregnancy loss are readily identified and approached. These settings will include hospital-based emergency departments, hospital-based ultrasound units, and private obstetrician/gynecology practices. Clinical sites will be sent a letter describing the study objectives, the goals of the trial and the need for subjects, and will be given study brochures and/or study contact forms for use in their office. The brochures will include a description of the study, some basic Q&A and a telephone number with information on how to reach study personnel. Women can either contact study coordinators directly or give consent for study coordinators to contact them.

Community-based recruiting will be done through a wide variety of mechanisms, including disseminating information about the study through advertisement in the media, direct mail, placement of flyers in community centers, and appropriate website posts. Because of the success of tear-off posters, flyers and word of mouth in recruiting for previous studies, these methods will be employed with EAGeR as well.

D.2. Study Visits and Contacts Overview

The planned follow-up schedule for the EAGeR trial includes an initial telephone screen for general eligibility, an in-clinic screening visit including baseline assessments and measures, and then an enrollment/randomization visit (day 2-4 of menstrual cycle), followed by two menstrual cycles of active follow-up (or until becoming pregnant, whichever comes first), followed by four months of passive follow-up (or becoming pregnant, whichever comes first). Active follow-up entails collection of questionnaire data and frequent specimen collection. Women not becoming pregnant within the first two cycles will remain in the study under passive follow-up for four additional cycles (or until becoming pregnant, whichever comes first). Those not pregnant by the end of passive follow-up will complete study participation. Women who become pregnant in the six months of study follow-up will be followed throughout gestation for pregnancy outcomes. During the one month following conception, the pregnant women will undergo close monitoring with specimen collection and clinical evaluations. Details of study visits are as described below.

To be considered a completer, a study participant must complete at least 90% of her scheduled clinic study visits (number of scheduled visits will vary depending on the length of follow up).

The outline of participants' follow-up in the EAGeR trial is depicted in Figure 1.

Figure 1. Participants' management in the EAGeR Trial

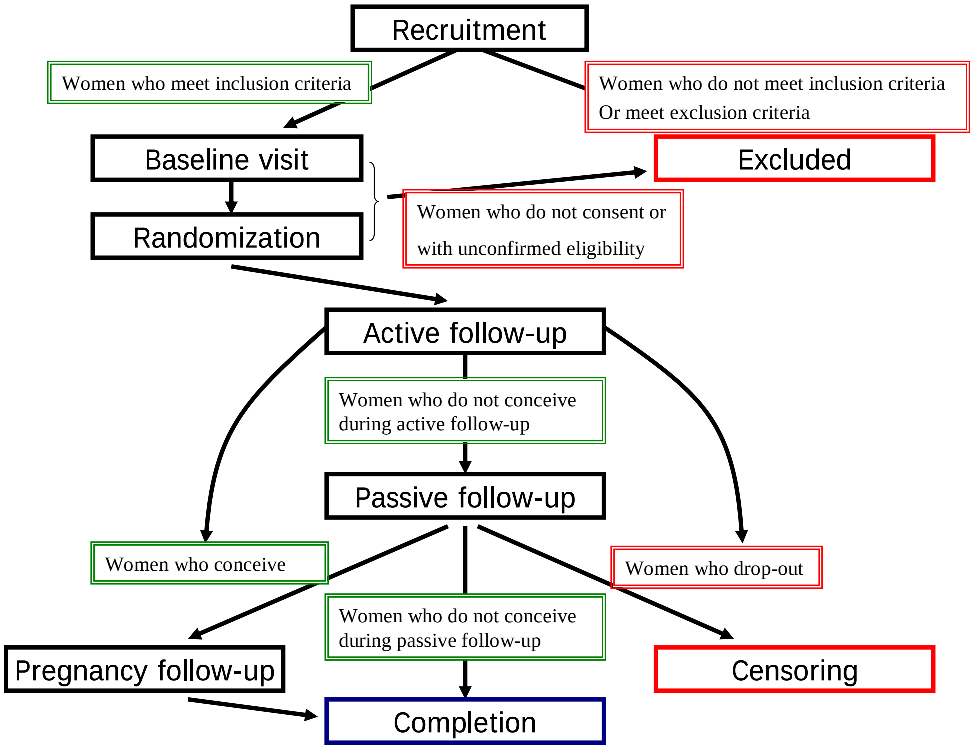

D.3. Screening process

Initial screening may take place in person or by telephone. Women who meet initial screening criteria will be invited to participate in the study and scheduled for a baseline visit in the study clinic at the earliest convenient date. Study personnel will obtain informed consent at the baseline visit for all participants.

Telephone Screen:
Preliminary contacts with interested women can be made directly with study personnel by phone or by email/web inquiries. Regardless of how initial contact is made, each potential participant will be contacted by telephone by one of our staff.

When the initial call comes into the clinic, the potential participant will be greeted warmly and the staff will ask a few introductory questions including asking their name, address, telephone number(s), how they heard about the study and their current age. The staff will then provide a basic overview of the study including the objectives and the basic

study requirements. They will be given an opportunity to ask questions. After the initial questions have been answered, the caller will be told that there are some initial screening questions that we will need to be asked to determine their basic eligibility. They will be asked to answer a series of eligibility questions (see Appendix 1). The telephone screen will be completed in its entirety, regardless of their answers to any individual question. The complete list of general eligibility questions (entire telephone screen) will be asked before a determination is made regarding eligibility in order to better characterize the prospective participants (all the reasons they are ineligible). Questions on the source of referral and how they heard about the EAGeR trial will be included on the screening questionnaire for recruitment tracking purposes.

If the woman is found to be *ineligible*, she will be informed that there are certain criteria that are predefined for study inclusion and that she does not meet these eligibility criteria. They will be thanked for their time and interest and the call will be ended. If they are determined to be *eligible* based on initial screening criteria, we will inform them that they do in fact meet our initial eligibility criteria. They will then be provided with additional details about the study and asked to schedule a baseline appointment at their convenience. They will be asked to provide additional contact information. A screening form script has been developed to ensure standardization of the screening process and to ensure information given to study participants is consistent (see Appendix 1).

If a baseline appointment is made after the telephone screening, the informed consent form will be mailed to the participant to read over before the baseline appointment. The consent form will be discussed in detail and signed at the baseline visit.

D.4. Baseline study visit (1st clinic visit, V-BL)

The baseline visit will entail informed consent, detailed eligibility, and data collection including administered questionnaires, physical measurements and laboratory testing (urine samples).

At this first baseline clinic visit, each potential participant will meet with the study coordinator or research assistant. At this visit, study objectives, participation requirements, and the consent form will be described in detail. After signed consent is obtained, a detailed eligibility questionnaire will be administered to ensure that the woman fits the inclusion criteria and has no conditions that could fit the exclusion criteria. Next, a urine specimen will be collected and a urine pregnancy test will be performed to confirm the woman is not pregnant (if pregnant, she will be excluded from the study). Study ID number will be generated at this visit.

Baseline questionnaires will be interviewer-administered and entered on computerized Web-forms.

The following questionnaires will be administered to elicit information on socio-demographic characteristics, health-related behavior and medical history (See Appendix 2 for the baseline questionnaires). Reported menstrual and reproductive history will be recorded in detail.

- Demographic Questionnaire

- Health and Reproductive History Questionnaire Part B
- Exercise Questionnaire
- Lifestyle Questionnaire
- Occupation Questionnaire

Participants will be given the Personal Information Questionnaire, Family Medical History Questionnaire and Part A of the Health and Reproductive History Questionnaire to be completed at home. Participants will bring the completed forms to the next clinic visit (the randomization visit) where study staff will review the forms for completeness and answer any questions. These questionnaires are sent home with the participant to reduce the amount of time spent at the research clinic and since the participants may need to use home records or other contacts in order to complete the questionnaires.

In addition to these questionnaires, blood pressure and anthropometric measurements (height, weight, waist to hip ratio, skin fold measurements) will be taken (See Appendix 2 for the Physical Measurements Form). Participants will also be asked to insert a vaginal swab to provide a Gram stain sample.

Participants will also receive brief instructions for the use of fertility monitors and specimen collection kits. These directions will be reinforced in more detail at the randomization visit.

At the end of the baseline visit, those subjects that remain interested and eligible will be scheduled for an active study visit where final enrollment and randomization will take place. Based on the menstrual history and the expected date of the start of the next menstrual cycle, the next appointment will be made to coincide with day 3 (2-4) of the next menstrual cycle. Delay of randomization until this visit allows additional opportunity to assess the commitment of the participants and to minimize dropout during the trial.

In addition to the baseline questionnaires, participants will be asked to provide authorization (i.e. release) to send a brief questionnaire to their gynecologist to secure details regarding the reported spontaneous abortion (SA) including date and results of the serum hCG, date and gestational age at the time of the SA, ultrasound findings (if completed), histology, and course following SA (i.e. D&C). (See Appendix 3 for the event verification form).

D.5. Randomization

D.5a. Randomization Visit (V-RD)

Following the baseline visit, participants will return for the final enrollment and randomization visit. The randomization visit constitutes the first active follow-up visit and its content aside from the randomization is detailed in the next section, ***D.6a.***

Briefly, during this visit, participants will be randomized following the protocol described below, receive their assigned treatment (3 month supply), and receive the testing materials for the study, i.e., fertility monitors, specimen collection kits, and daily diaries. The study staff will review the instructions with the participant again. Randomization will

be in the ratio of 1:1, treatment to placebo stratified by eligibility group and clinical center. Participants will be counseled to begin taking treatment daily starting that day. The next active follow-up visit will be scheduled for approximately the mid point of the current menstrual cycle.

Pill Dispensation: Study Pills and folic acid will be provided in bottles with a three month supply. Each Study Pill bottle will have enough pills to amply cover cycles 35 days long (105 capsules). Folic acid will be supplied in bottles containing 100 tablets (due to how the folic acid is commercially available). Bottle labeling and bar code verification will be established as described under "Randomization protocol section D5b below. Bottles, both unopened and opened, will be brought in for weighing at each clinic visit throughout the study to evaluate adherence.

At the randomization visit, participants will be given fertility monitors to help them predict their fertile period and time intercourse to maximize the likelihood of becoming pregnant. The fertility monitor will also provide information on the participant hormone levels and cycle for research purposes. The fertility monitor translates urine dipstick hormone measurements into predictions for the fertile period and is suitable for women whose menstrual cycle length falls within 21-42 days regardless of regularity. The monitor tracks the urinary metabolite of estradiol – estrone-3-glucuronide (E3G) and luteinizing hormone (LH) determined from dipsticks and displays the corresponding results (i.e., low, high or peak fertility days). The peak fertility days will also be used to schedule subsequent appointments during active follow-up.

D.5b. Randomization Protocol

Randomization of subjects will be carried out to obtain the 1:1 allocation ratio between the treatment and placebo arms. Randomization will be stratified by center and by eligibility criteria group. Two eligibility groups will be considered: group I including all women who have had only one spontaneous abortion in gestation week <20 and within the last 12 months, no more than one live birth in the past and no pregnancy losses occurring on or beyond 20 completed weeks of gestation; group II includes women who do not fit group I but are eligible by the modified criteria. A computer algorithm will create the random assignment to one of the treatment arms based on random permuted blocks design with randomly varied block size.

The random sequence of treatment assignment will be linked to a pre-determined list of study ID numbers generated at the Haifa DCC for each clinical center and to a corresponding non-informative list of medication bottle numbers for the treatment assignment. This list will reside on the local computers at the DCC and a mirror copy (encrypted) will be kept in a different secured location. As soon as an allocation is done, the information will be sent automatically to the server and the master randomization file will be updated accordingly.

A list of subject Study ID numbers will be created a-priori for each clinical center. At the baseline visit, when a new participant is enrolled, a new Study ID will be automatically generated through a secured link in the study's Web-based system. In the randomization

visit, the computer system will generate the treatment assignment for the current participant and the Bottle ID number for Study Pill will appear according to the randomization scheme. A subject will not be randomized without an indication of a signed informed consent and a confirmed eligibility.

There are several layers of masking mechanisms. The Study ID numbers are generated automatically, and the clinical center staff will not be able to obtain IDs without first enrolling a participant; the randomized treatment assignment will also take place automatically without any possibility of change at the clinical site end. The boxes containing the Study Pills are pre-packaged with identically looking pill bottles containing either aspirin or placebo. During the entire duration of the study, neither the participant nor the clinical centers' personnel will have any access to data that will enable de-coding the randomization scheme. Unblinding procedures are in place as needed but are anticipated to occur rarely.

The automated randomization scheme allows the process to take place in distant geographical locations as it does not require direct contact with a DCC staff member. However, in case any problems arise, the Haifa DCC operates a telephone hotline for reporting any urgent problems allowing for quick resolution.

D.6. Active follow-up

The active phase of follow-up will last for up to two menstrual cycles of participants and will entail collection of the following data: daily urine specimens, blood samples at mid-cycle 1, end-cycle 1, mid-cycle 2, end-cycle 2 by venipuncture, and daily diaries.
Active enrollment also entails collection of brief questionnaire data to evaluate adherence to treatment and the occurrence of possible adverse reactions to the Study Pill and folic acid given on trial. Once a women tests positive on a pregnancy test she will transition into the pregnancy follow-up schedule described in section D.8. In the following description of the non-pregnancy follow-up we use the terms "cycle" and "month" interchangeably. However, the exact study duration for each woman in this phase will be based on her cycle length and is therefore variable among participants.

D.6a. Active Follow-up Visits:

The first active follow-up activities are performed at the randomization visit (V-RD). The date of this visit will be scheduled to coincide with day 3 (day 2-4) of the participant's expected next menstrual cycle. In addition to the activities described above for the randomization visit (Section D5), the following activities will be performed at this visit:

- Again review the study requirements, answer any participant questions and review a brief update of eligibility criteria (any changes since baseline visit).
- Review part A of the Health and Reproductive History Questionnaire, the Family Medical History Questionnaire, and the Personal Information Questionnaire that the participant will have completed at home prior to the visit
- Provide the daily diary and instructions on how to complete it. Participants will be asked to complete the diary for the previous day and this will be reviewed with them to ensure understanding and completeness.
- Blood and urine samples will be collected.

- Provide the participant with the Clearblue Fertility Monitor (and testing sticks) and urine collection materials and review instructions for the use of the fertility monitors as well as instructions for home urine collection and storage.
- Provide study bags and a small cooler to be used for transport of diaries, monitors and home urine specimens.
- Provide the participant with a 3-month supply of Study Pill and folic acid. Instruct participants to bring all pill bottles back to each visit whether or not empty.
- Schedule the next visit to correspond to mid-cycle, about day 14 of a 28 day cycle (adjusted for cycle length). They will be asked to bring in their diaries and monitors and all urine samples collected in to the visit.

Between these active follow up appointments, participants will collect daily urine specimens (self storage by patient), complete daily diaries, and conduct fertility monitoring.

Specimen collection
First morning urine specimens will be collected daily by the women during follow-up in order to measure levels of human chorionic gonadotropin (hCG), as well as other relevant hormones. All women will begin collecting urine on day 3 (2-4) of their first study menstrual cycle (at the randomization visit) and continue collection at home through active follow-up or until they become pregnant. A two-week supply of pre-labeled urine specimen kits will be given to each participant at the beginning of the study and they will be resupplied as necessary. All specimens will be maintained in the participant's freezer and collected approximately every two weeks at the time of clinic visit where serum collection will also be done. Serum will be collected every two weeks during active follow-up on approximately the 14th and 3rd day of each cycle. During pregnancy, serum and urine will be collected according to study protocols (Section D.8).

The main purpose in collecting daily urine specimens during the active phase (and first month of pregnancy) is to capture early, sub-clinical losses by quantitative hCG testing. Qualitative hCG determinations will be used to document pregnancy, implantation, and early pregnancy loss. These data, taken together with clinical and ultrasound data, as well as serum hCG, will help categorize outcomes into biochemical or clinical pregnancies.

Stored serial urine samples can also be used retrospectively for determination of the LH surge and the progesterone metabolite pregnanediol glucuronide as a urinary marker of ovulation. These measurements promise more accurate laboratory determinations of LH surge than the Clearblue Fertility Monitor can provide. About 9% of cycles with the Clearblue Monitor do not have a clearly identified LH surge. Stored urine samples also will be used to assess treatment compliance (by assessing aspirin metabolites), physiologic potency of the aspirin therapy (by assessing eicosanoid metabolism), and as a repository for future assays.

Specimen repository
Samples collected that are not tested as part of the current protocol will be used to generate a specimen repository. All specimens will be processed and transported to the NICHD in a schedule according to study protocols.

D.6b. Active Phase Diaries (See Appendix 4)
Daily Diaries: Variables to be recorded daily during active conception attempts
- Use and results of the Clearblue Fertility Monitor
- Taking of study pills and folic acid (adherence measure)
- Bleeding or allergic signs or symptoms referable to the use of aspirin
- Intercourse occurrence
- Menstrual bleeding
- Unusual observed pelvic complaints such as pain and bleeding
- The use of self-imposed formulas, herbs or pharmaceuticals for conception (or contraception) attempts
- Concurrent illness
- Concurrent medications used
- Alcohol, tobacco and caffeine exposure
- Stress

D.6c. Active Visit 1 (Month 1, Mid-Cycle) (V-A1): At the mid-cycle visit for month 1 (about day 14 of a 28 day cycle) we will again review the study requirements and answer any participant questions. In addition we will:
- Collect home (frozen) urine specimens from participants; provide participant with another kit for the next two weeks of home urine collection.
- Collect in clinic blood and urine specimens.
- Collect and review daily diaries.
- Do an assessment of adherence using the Adherence questionnaire (See Appendix 5) and weighing pill bottles– address any problems reported.
- Weigh pill bottles to verify self-report and then return bottles.
- Conduct a safety interview regarding aspirin use (See Appendix 5).
- Collect information from fertility monitors (and discuss any issues found regarding daily use or other problems detected).
- Review instructions for next reporting period for urine collection, monitor and diaries.
- Schedule next appointment (for the estimated end of cycle date + 3 days to coincide with day 3 of the next menstrual cycle).

D.6d. Active Visit 2 (Month 1, End-Cycle) (V-A2): At the end-cycle visit for month 1 (day 3 [day 2-4] of the next menstrual cycle) we will again review the study requirements and answer any participant questions. In addition to activities done at the mid-cycle 1 visit (V-A2), we will verify pill adherence by weighing the pill bottles and will do a pregnancy test on collected urine (only if the participant has not started menstruating by this visit). The visit will include:
- Collect home (frozen) urine specimens from participants.
- Collect in clinic blood and urine specimens.
- Pregnancy test (urine stick) in clinic only if the participant has not started menstruating by this visit.*
- Collect and review daily diaries.

- Do a verbal assessment of adherence – address any problems reported (see Appendix 5).
- Do bottle weighing to verify self-report and return pill bottles.
- Conduct a safety interview.
- Collect information from fertility monitors (and discuss any issues found regarding daily use or other problems detected).
- Review daily diaries.
- Review instructions for next reporting period (urine collection, monitor use and diaries).
- *If the pregnancy test is negative, we will provide an updated calendar for testing and schedule next appointment (for about the mid-point of cycle 2). These subjects will have one additional month of active follow-up (see Active Month 2). HOWEVER, if the urine stick test is positive, subjects will transition in to pregnancy follow-up. Those subjects will be seen according to 1st Pregnancy Month visit schedule.
- If the participant has not started menstruating by this visit but the clinic pregnancy test is negative, she will be instructed to take a home pregnancy test (HPT) 5 days later. If the HPT is positive, she will need to return to the clinic within 3 days to start the pregnancy follow-up. If the HPT is negative (or she starts menstruating before the 5 days), the participant will continue as previously scheduled. If she does menstruate this month and continues to have a negative HPT, she will be considered as having an anovulatory cycle.

D.6e. Active Visit 3 (Month 2, Mid-Cycle (V-A3) (for subjects not pregnant in month 1) (V-A3): At the mid-cycle visit for month 2 (about day 14 of a second 28 day cycle) the protocol for V-A1(Month 1,Mid-Cycle visit) will be performed again (see above, D.6c).

D.6f. Active Visit 4 (Month 2, End-Cycle) (V-A4): The end-cycle visit for month 2 occurs on the expected day 3 (day 2-4) of the next menstrual cycle. The visit will include all activities as listed in Month 1, End-Cycle visit **V-A2** (see above, D.6d). In addition to the above listed V-A2 activities, participants will also be provided with another 3-month supply of Study Pills and folic acid (note: pills are provided one month in advance so that participants always have at least a one month supply). Participants will return the final daily diaries and home urine specimens. If pregnant, participants will transition into pregnancy follow-up. If not pregnant, participants will be instructed on the requirements for passive follow-up. At this visit, participants will be provided with a HPT and at least three days of emergency collection tubes to be used in case of a positive HPT. The next telephone contact and the following End-Cycle passive follow-up clinic visit will be scheduled.

D.7. Passive Follow-up

Women who do not conceive during the active follow-up phase will continue with treatment and enter passive follow-up for an additional four cycles or until becoming pregnant (whichever comes first). The passive follow-up schedule is less frequent than the active schedule and has fewer demands for specimen collection and diary reporting. Participants will continue taking study pills and conducting home fertility monitoring.

They will not need to collect urine for storage and freezing at home (they will continue using the monitors). Participants will have monthly visits with study staff to collect clinic samples and data, replenish pills, and to assess safety, adherence and pregnancy.

Phone calls between visits will be done at the mid-cycle point to assess any adverse event occurrences and reinforce adherence to the study pills.

D.7a. Passive Visits (V-P1 to V-P4): At the end-cycle monthly passive visits (Month 3, 4, 5 and 6 of study follow-up) participants will come into the clinic. The visits will occur at end-cycle + 3 days of the menstrual cycle (i.e., day 3 of menstruation).

At day 1 of expected menstruation, **ALL women without menses** will take a home pregnancy test (HPT). If the HPT is positive, women will use the three emergency urine collection tubes they should have and collect first morning urine until they come in to the clinic on their regularly scheduled visit at which point they will enter into pregnancy follow-up. She will be instructed to write the date on each of the urine collection tubes. If the HPT is negative, the women will still come in at the regularly scheduled visit but will not need to collect urine.

Passive follow-up visits will include:

- Completion of the passive-follow up questionnaire (See Appendix 6)
- Collection of a urine sample. Perform a pregnancy test (urine stick) in clinic only if the woman has NOT started menstruating.
- A verbal assessment of adherence – address any problems reported.
- Pill bottle weighing to verify self-report of adherence. (Distribute additional pills at V-P4).
- Completion of a safety interview.
- Collection of information from fertility monitors (discuss issues or other problems detected).
- Review of instructions for next reporting period (e.g., continue Study Pills and folic acid, continue using fertility monitor, conduct pregnancy testing if needed, complete the phone contacts at the mid-cycle, and come in for the next clinic visit at approximately end-cycle +3 day of the next menstrual cycle).
- If the pregnancy test is negative, we will provide an updated calendar for testing and schedule the next appointment. If the urine test is positive, subjects will provide a serum specimen for banking, and they will be seen according to 1st Pregnancy Month schedule.
- Instruct participant that any time she has a positive home pregnancy test, she needs to call the study coordinator to come into the clinic within 3 days of the positive test and to start collecting and freezing first morning urines until the day of the visit. She will be instructed to include the date on each of the urine collection tubes. She will need to bring the frozen urine samples to the clinic on the visit day.

D.7b. Passive Follow-Up Phone Contacts

The study staff will call participants at mid-cycle during passive follow-up. During these calls, following the script, the staff will administer the Safety Questionnaire and the Adherence Questionnaire. We will also reinforce the importance of adherence.

At the end of passive follow-up (end of 6th menstrual cycle), if no pregnancy is confirmed, subjects will be **closed** from regular participation in the study (no additional clinic visits), and the remaining medications will be collected. The non-pregnancy follow-up scheme is depicted in Figure 2.

Figure 2. Timeline for participant follow-up during attempt to conceive *

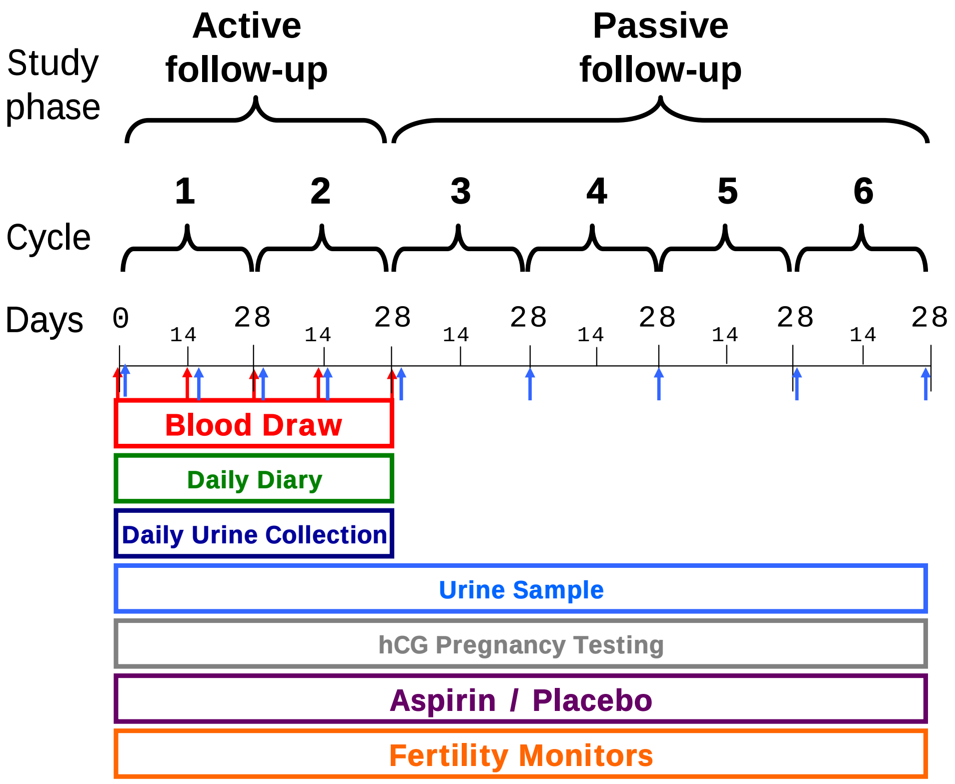

* Note: We use cycles of length 28 days as an example with day 14 indicating mid-cycle.

D.8. Pregnancy follow-up

Women will continue taking assigned study treatment assignment until 36 weeks gestation (or pregnancy outcome if sooner than 36 weeks) to avoid some potential risks associated with late aspirin use in pregnancy, including issues related to patent ductus arteriosus, bleeding with abruption and potential contraction inhibiting effects. Study personnel will actively follow participants for weeks 4-8 post detected pregnancy and then with periodic contact until the end of pregnancy. Women who experience an early hCG detected pregnancy loss (i.e., prior to clinical confirmation of pregnancy) will return to active or passive follow-up (depending on where they left off) until they have completed a total of six cycles of follow-up or become pregnant again, whichever comes first. The outline or pregnancy follow-up is described in Figure 3.

Pregnant women will be encouraged to receive prenatal care. Although this is not the responsibility of the trial, study coordinators will help facilitate access to care for willing participants without established providers.

1st Month of Pregnancy Visits: If participants become pregnant (regardless whether during active or passive follow up), they will conduct one additional month of home collection and storage of urines. In order to have samples to assess changes in urine hCG in subjects (both who miscarry and those who continue to maintain their pregnancy), we will collect urine for this entire month (30 days). During this first month, participants will also complete a pregnancy daily diary and have an early ultrasound at week 6.5 of gestation.

During pregnancy the following activities will occur:

- Telephone and personal contact with research nurses/staff to collect data, specimens, distribute pills, assess adherence, and conduct medication safety interview
- Participants will continue daily Study Pills (aspirin or placebo) and folic acid (given as three-month supply bottles) until week 36 of gestation.
- Daily home urine collection and storage for one month.
- Daily pregnancy diaries for one month.
- Completion of pregnancy follow-up questionnaire monthly either by phone or in clinic (See Appendix 7)
- Completion of Safety and Adherence Questionnaires as well as weighing the of the pill bottles to evaluate adherence in clinic visit.
- Completion of the Short Physical Measurements Form at each clinic visit
- A blood draw and urine sample at each study clinic visit.
- An additional study sonogram at 6.5 weeks of gestation.
- Obtain information from routine clinically indicated obstetric care including sonograms through medical record abstraction.
- Chart abstraction of medical records after pregnancy to assess obstetric outcome, sonograms, and prenatal laboratory values including the standard serum screen around 15-18 weeks' gestation (which is strongly correlated with placental insufficiency[128-129]).
- Maternal sera will be obtained at least once each trimester at the time of clinically indicated blood draw (initial visit, second trimester serum marker screen, glucola screen, and time of delivery (in Utah only)).
- In Utah, women will be given a container to collect tissue should they experience miscarriage during the study.

The University of Utah will perform eight visits during pregnancy based around routinely scheduled prenatal clinic visits whenever possible to minimize the burden on participants. When study visits are completed at the participant's physician's office (rather than the research clinic), all typical visit activities will still be performed except not all the information on the Short Physical Measurements Form may be available. The week 8 pregnancy visit needs to occur at the research clinic since new randomized pill

bottles will need to be obtained for the participant. This supply of pills should be enough to cover the period until week 36 of pregnancy. In Utah, the approximate times of the visits will be: week 4, 8, 12, 16, 20, 28, 36 and parturition (See Table 1 below for timing of contacts and activities). The last active or passive follow-up visit where the positive pregnancy test was obtained is considered week 4 of pregnancy. Specimen collection will be obtained at the 8, 12, 16, 20 and 28 week visits. If the 12, 20, and 28 week visits are done at the participant's physician's office and routine labs are to be drawn at that visit, every effort will be made to obtain the study labs at the same time. There will be no specimen collection at the 36 week visit. Utah will perform monthly phone calls at weeks 24, and 32 to conduct the safety interview, adherence assessment, and the monthly pregnancy questionnaire. The study ultrasounds will be performed at separate visits at one of the four research clinic sites and will be conducted at 6 ½ weeks to confirm and date the pregnancy. Utah will also collect placenta at the time of miscarriage (when feasible) and after delivery. Cord blood will also be collected at the time of delivery when feasible.

The University at Buffalo will perform six pregnancy visits at weeks 4, 8, 12, 16, 20, and 28. The last active or passive follow-up visit where the positive pregnancy test was obtained is considered week 4 of pregnancy. Specimen collection will be obtained at the 8, 12, 16, 20 and 28 week visits. A study ultrasound will be performed at 6 ½ weeks to confirm and date the pregnancy. In between study visits (at weeks 24, 32, and 36), Buffalo will perform monthly phone calls to conduct the safety interview, adherence assessment, and the monthly pregnancy questionnaire.

The outline or pregnancy follow-up is described in Figure 3 below.

Figure 3: Timeline for participant follow-up during pregnancy

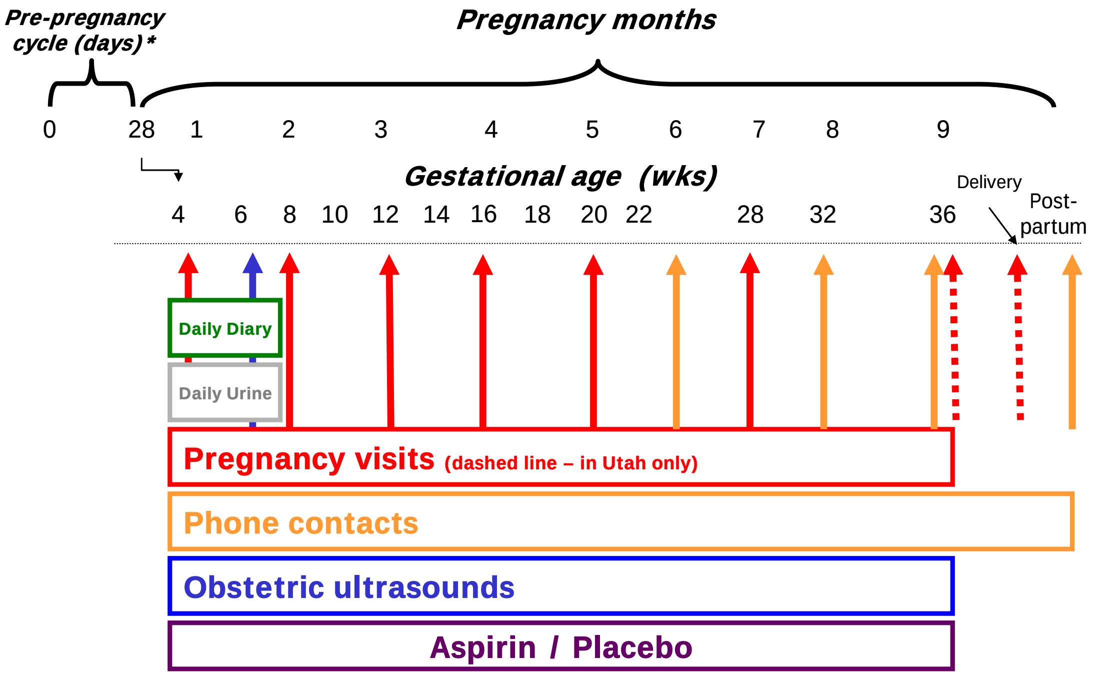

* Day 28 represent the end of cycle visit with a positive pregnancy test

Table 1. Activities performed during pregnancy follow-up visits

	Pregnancy Visits								
	Week gestational age								
**Utah & Buffalo**	4	6.5	8	12	16	20	28	36 (Utah)	Parturition
Blood Specimen	X		X	X	X	X	X		X (Utah)
Urine Specimen	X		X	X	X	X	X		
Home Daily Urine Collection	X	→	X						
Pregnancy Daily Diaries	X	→	X						
Study Ultrasound		X				X (Utah)			
Blood Pressure	X		X	X	X	X	X	X	
Anthropometric Measurements	X		X	X	X	X	X	X	
Pregnancy Questionnaire				X	X	X	X	X	
Safety Interview	X		X	X	X	X	X	X	
Adherence Questionnaire	X		X	X	X	X	X	X	
Placenta/Cord Blood Collection									X (Utah)

Closeout of Pregnant Participants and Outcome Ascertainment: Closeout will occur for all pregnant subjects within six to eight weeks of their final outcome (miscarriage, stillbirth, live birth, other). Any remaining study medications will be collected from the participants. A postpartum questionnaire will be conducted over the phone approximately six weeks after the participant's endpoint occurrence to obtain information about late complications for mother and child. Closeout will occur after the completion of the chart abstraction form for the course of pregnancy, delivery and late complications.

Miscarriage: For those experiencing SA information on dates, symptoms, procedures and location of visits to physician(s) will be asked. Where applicable, pathology records will be requested. A release(s) for records from their personal physician, any testing or treatment centers will also be secured to verify outcomes reported and dates. Information on support groups or counseling will be provided to these participants. The University of Utah will be taking bloods and performing karyotyping of the miscarriage tissues where tissue is available.

Stillbirth: For those with pregnancy loss after 20 weeks (stillbirth), information on dates, symptoms, procedures and location of visits to physician(s) will be asked. Where applicable, pathology records will be requested. A release(s) for records from their personal physician, any testing or treatment centers will also be secured to verify outcomes reported and dates. Complete information on the course of this pregnancy outcome will be abstracted from the physician and hospital records including information on both maternal and fetal complications. Information on support groups or counseling will be provided to these participants. The University of Utah will also be taking bloods when possible.

Live birth: For those who have a live birth, regardless of gestational age, information on dates, course of delivery, procedures and location of birth will be asked. A release(s) for records from their personal physician, any testing or treatment centers will also be secured to verify outcomes reported and dates. Complete information from hospital and physician records regarding gestational age, fetal growth, birth defects and type of delivery will be abstracted. Information on both the mother and the infant will be collected from the records using standardized abstraction forms. Those live births with complications occurring in either the mother and/or infant will be treated with careful sensitivity. Information on support groups or counseling will be provided to participants who have negative events occurring during or after delivery.

D.9. Specimen Collection

All blood, tissue and urine specimen (including clinic urine samples and the daily home urine samples) collected during the EAGeR study will be initially processed and stored at the local sites in -80F freezers. .

Later, ALL specimens collected from the clinical sites will be sent to the NIH Tissue Repository (Fisher Bioservices: 627 Lofstrand Lane, Rockville, MD 20850 – 301-762-1772). The exact schedule and protocol for shipping the specimen to the central repository will be determined at a later date, taking into account the rate of enrollment and the storage capacity at each center.

D10. Retention Plan and Compensation

D.10a. Retention Plan: Retention of the research subjects will be one of the most difficult tasks of this study. There are frequent clinical visits that require blood and urine collection at each. Timing of these visits is important. We are enrolling subjects of an age that include many working women and mothers. As such, we need to minimize burden as much as possible. Our belief is that retention begins at the time of recruitment. At recruitment it is essential to establish a positive rapport with the research subjects. This includes providing information in a friendly manner, being responsive to their concerns, providing subjects with enough detail to understand the requirements of participation and stressing the importance of the potential research findings. For this study, the importance of timing and frequency of study visits and sample collection need to be stressed clearly. Awareness of the time commitment will be integral. The following steps will be taken:

1. **Retention begins with recruitment.** Spending time at recruitment to fully explain the time commitment and importance of the study. Enroll only those subjects who understand the commitment. An informed participant is the best participant.
2. **Limit burden where possible.** Assess every aspect of the study to find ways to limit participant burden without compromising the study goals. Limit questionnaires and procedures to the most relevant measurable items.
3. **Provide an inviting research environment.** Staff need to be fully accommodating and friendly. The clinical space should be clean and professional. Make the participant feel welcome and at home in the clinic.
4. **Be on time.** Never make your participants wait. Show them that their time is valuable to you!
5. **Provide easy access to the clinic and close convenient parking.** Easy access for parking and using public transportation is a must.
6. **Listen.** Listening to their concerns is a key step in finding a solution. Keep communication open. Our staff have all been trained on *motivational interviewing*, a technique used to aid in retention of subjects having difficulty. MI allows the participant to reevaluate reasons for participation and negotiate their own plan to get back on goal.
7. **Obtain additional contact information.** Obtain a phone number of a friend or relative of the subject (one they are comfortable with), so that if they move or change their number, they can still be contacted.
8. **Incentives.** We use all sorts of inexpensive incentives to keep participants on track. They are fun and intended to reward subjects. Some examples are “gold stars” for those who complete forms, small tokens such as pens, refrigerator magnets bags or other items as rewards for participation, recognizing anniversaries, and follow-up notes that are personalized thanking them for their valuable time.

D.10b. Compensation:

Participant's compensation for the EAGeR trial will be site specific.

University of Utah
Participant compensation

Participants will receive a total of up to $ 250.00 remuneration for participation in the study. This will be distributed on a serial basis so as to avoid coercion and reward compliance as outlined below.

1. Baseline: $10.00 upon completion.
2. Enrollment: $40.00 upon completion. This will be paid after completion of the randomization visit.
3. Pregnancy: $100.00 upon becoming pregnant. Must have a positive pregnancy test at the clinic site and return the fertility monitor to receive payment. Will be paid for one positive pregnancy while in the study.
4. Completion: $100.00 upon completion of the study. Must turn in fertility monitor to receive payment.
5. Mileage reimbursement: $0.485/mile or the federal allowance rate for study visits at the site with the research nurse.

The participant will be required to sign the participant reimbursement form at each study visit. This form will be kept in the patient chart.

University of Buffalo
Participant compensation
In order to increase both recruitment rate and retention, all subjects will be compensated for their travel expenses. During this time they will also be required to keep diaries, return questionnaires and provide blood and urine samples. This is considered exceptional time burden over a fairly condensed period of time. The amount of money we offer is modest ($10 per clinic visit) and includes payment for participant travel expenses and effort. These payments will be processed every 3 months. Participants will complete vouchers at the end of each visit (required by University rules).

D.11 Sub-studies
D. 11. a. One sub-study will be conducted in the EAGeR trial to evaluate compliance with Study Pills. The sub-study will be based on analysis of urine samples for the presence of aspirin metabolites. The evaluations will be done on a 5% sample of the women participating in the study; that is about 80 samples in total. The sampling scheme will be developed by the DCC. For the sub-study to provide the most useful information, the sampling plan needs to consider possible variability in compliance among subjects, over time and with pregnancy status of the woman. We plan to sample 30 participants in month 2, 30 in month 4 of the non-pregnancy follow-up and 20 in the second month of pregnancy. The sample will include women in both treatment arms with a ratio of about 4-5:1 of aspirin versus placebo. This condition will help maintain the masking of treatment allocation and also allow compliance evaluation for women in the placebo arm

who may be taking aspirin independently. The sampling scheme will also allow for weighting of probabilities of selection by the reported adherence of the participants.

D.11.b Another sub-study will evaluate the role of adipocyte cytokines in the risk for gestational diabetes. This sub-study is titled: A study of novel adipocyte cytokines and the risk of gestational diabetes mellitus.

Background:
Gestational diabetes mellitus (GDM) is among the most common complications of pregnancy. Approximately 3-7% of all pregnancies are complicated by GDM, resulting in more than 200,000 cases annually (1). Importantly, this number is likely to increase as the prevalence of obesity among reproductive age women increases. Recent data have shown a substantial rise in the incidence of GDM from 1991 to 2000 (2) and a doubling from 1994 to 2002 (3). Women with GDM have increased risk for prenatal morbidity and a considerably elevated risk for impaired glucose tolerance and type 2 diabetes mellitus (type 2 DM) in the years following pregnancy. Children of women with GDM are more likely to be obese and have impaired glucose tolerance and diabetes in early adulthood. Although the precise underlying mechanism is yet to be identified, insulin resistance and inadequate insulin secretion to compensate for it play a central role in the pathophysiology of GDM. The metabolic challenges related to pregnancy reveal an otherwise undetected predisposition to glucose metabolic disorders in some women.

Excess adiposity is the single most important modifiable risk factor for the development of GDM. Mechanisms linking excess adiposity to elevated risk of GDM are not completely understood, but recent evidence points to a crucial role of specific hormones and cytokines (“adipokines”) secreted by the adipose tissue. A major breakthrough in understanding the link between adiposity and glucose intolerance has come from the demonstration of a cross-talk between adipose tissue and other insulin target tissues such as skeletal muscle and liver. Such cross-talk is mediated by a number of molecules that are secreted by adipocytes and act in an autocrine, paracrine, or endocrine fashion Among those identified to date are retinol binding protein 4 (RBP4), resistin, leptin, IL-6, and adiponectin (in particular, high molecular weight adiponectin). In concert, these cytokines, or 'adipokines', are believed to adapt metabolic fluxes to the amount of stored energy. Dysregulation of this network is believed to be a critical factor in the deterioration of insulin sensitivity that accompanies adiposity.

Despite the promising role of these novel adipocyte cytokines in the pathophysiology of insulin resistance, prospective studies of these adipokines in association with EAGeR GDM risk are sparse. The majority of limited available studies are small cross-sectional studies, inferences from which were hampered due to the ambiguous temporal relation between these adipokines and GDM risk. The major aim of this project is to prospectively investigate novel biochemical markers involved in adipokine secretion as predictors of GDM. In addition, we will examine whether these associations are independent of or modified by measures of overall and abdominal adiposity (BMI and waist circumference), and whether the associations between adipokines and GDM risk are mediated through elevated plasma free fatty acids.

Study design: A nested case-control study of 200 GDM cases and 400 controls based

on longitudinal data from 2 prospective studies.
1. EAGeR Trial (PI Dr. Enrique Schisterman): In this study, 1600 women with a history of miscarriages will be recruited. Among them, approximately 960 women (60%) are expected to have successful pregnancy in 6 months of trying. Approximately 40-70 GDM cases are expected to result from this study. For these cases we will select 80-140 controls.

2. The National Standard for Normal Fetal Growth study (PI Dr. Jim Zhang): In this study, 2400 low risk women will be recruited. Among them, there would be 70-90 GDM cases. We will add an arm of 600 high risk pregnant women (being obese) to this study, among which there would be at least 60 GDM cases.

By combining these two studies, we expect to obtain 170-220 GDM cases. Longitudinal blood samples including before pregnancy (EAGeR trial), during the 1st, 2nd, and 3rd trimester and 6 weeks postpartum (Fetal Growth Study) will be collected.

Adding a blood collection in the 16th gestational week to the EAGeR trial will enable the investigation of changes in adipokine levels every four weeks before clinical GDM screening test (24-28 weeks), which is a crucial time window for studying the pathogenesis of GDM. The approximately 4 bio-specimens from each woman before GDM screening test will allow a greater precision in evaluating the longitudinal trend of adipokine levels and other important molecules within individual women. Moreover, the inclusion of 16 gestational week blood collection will enable the time windows for bio-specimen collection in the EAGeR trial to be comparable to those in the Fetal Growth Study, another data source for the 'Adipokines and GDM' project.

Data collection and pregnancy follow-up
The new "Week 16" visit will be identical to other pregnancy clinic visits with the same questionnaires administered and same blood and urine samples obtained. The consent for this sub-study is covered by the original consent.

REFERENCE
1. Gestational diabetes mellitus. *Diabetes Care.* Jan 2004;27 Suppl 1:S88-90.
2. Ferrara A, Kahn HS, Quesenberry CP, Riley C, Hedderson MM. An increase in the incidence of gestational diabetes mellitus: Northern California, 1991-2000. *Obstet Gynecol.* Mar 2004;103(3):526-533.
3. Dabelea D, Snell-Bergeon JK, Hartsfield CL, Bischoff KJ, Hamman RF, McDuffie RS. Increasing prevalence of gestational diabetes mellitus (GDM) over time and by birth cohort: Kaiser Permanente of Colorado GDM Screening Program. *Diabetes Care.* Mar 2005;28(3):579-584.

E. STUDY TIMELINE

The study duration is five years. The first 6-8 months are considered as Phase I of the study and are used for study set-up, developing and writing of the study protocol, MOP, questionnaires and developing the study's web-site and computerized data entry system and securing IRB approval. This time will also be used to obtain the study pills and all the materials needed for the study as well as the recruitment materials. Phase II of the study starts with recruitment and continues with participants' follow-up and data collection until the end of months 57. The last three months of the trial are Phase III which is the wrap-up time used for final analyses and writing of reports. The timing of the segments of the study from the start of funding is shown in Table 2 below.

Table 2. Study phases and activities by months from start of funding

		**Phase**										
		**I**					**II**					**III**
		**Year 1**		**Year 2**		**Year 3**		**Year 4**		**Year 5**		
Task	Time (Mos.)	**0-6**	**7-12**	**13-18**	**19-24**	**25-30**	**31-36**	**37-42**	**43-48**	**49-54**	**55-57**	**58-60**
Set-up and training	0-6											
Recruitment	7-40											
Active follow-up	8-43											
Passive follow-up	10-47											
Pregnancy f-u	8-57											
Close out	58-60											

F. STUDY ADMINISTRATION

Groups and subcommittees

The Steering Committee

The Steering Committee for the trial provides leadership in ensuring the scientific integrity of the study, while conforming to the ethics and standards of practice promulgated by the American College of Epidemiology. This is in addition to adherence to protection of human subjects according to the specifications of the authorizing IRBs. The steering committee includes the principle investigators at the clinical sites and the DCC as well as the Project Officer at NICHD. This committee will make all final protocol decisions.

Data and Safety Monitoring Board

A Data and Safety Monitoring Board (DSMB) will be established to provide independent guidance to the study conduct. The members of the DSMB will include experts in relevant fields, potentially to include endocrinology, fertility, reproductive epidemiology, biostatistics and bioethics. DSMB members are appointed by the Director of the NICHD. The DSMB reports to the NICHD director through the NICHD Project Officer and the DESPR Director. The DSMB is charged with reviewing all interim data for safety and efficacy and for recommending to the Project officer any actions that should be taken to insure the ethical conduct of the trial. Results of all such reviews will be available to the investigators upon completion of the trial and unblinding. Final decisions for study continuation or discontinuation will be made by the NICHD Director. The DSMB will also have to provide periodic reports for submission to site IRB's stating the study continues and other necessary updates.

Other study committees

Additional special committees including endpoints committee and publishing committee will be established during the evolution of the trial.

G. STATISTICAL ANALYSIS

G.1 Overview

The EAGeR trial is designed as a multi-site randomized double blind clinical trial with two treatment arms: folic acid + aspirin compared to folic acid + placebo. A fixed sample size of 1600 randomized complete subjects is planned. Thus, for planning and statistical power computations we assume that this is the number of subjects who complete the trial and does not include dropouts.

The primary analysis plan is based on an "intention-to-treat" approach comparing the two treatment arms based on the randomized assignment, ignoring later changes in treatment. This analysis is described in Section G.2. The Haifa DCC will perform periodic partial analyses and present interim reports to the DSMB as requested, during Phase II of the trial. It is anticipated that partial analyses will be performed every 6-12 months. The final analysis will be performed upon completion of data collection and editing in Phase III of the trial. Also one full formal interim analysis is planned and the power calculations with considerations for the choice of optimal time for the analysis are given in Section G.3.

In addition to the intention to treat analysis, the Haifa DCC will engage in methodological research with the Project Officer at the NICHD. The focus is on developing statistical methods for clinical prediction models of reproductive outcomes as described in Section G.4, analysis of intermediate endpoints (Section G.5) and evaluating the effect of dropouts and compliance on the study results (Section G.6).

G.2 Study endpoints and intention to treat analysis

G.2.1 Study endpoints: operational definition and verification

Study endpoints will be determined based on self-reports, self-administered pregnancy tests, clinic pregnancy tests, medical record evaluations (including routine pregnancy follow-up, ultrasound) and laboratory tests. Whenever possible, we will verify the endpoints using different sources of information. For example, a report by a woman of a positive pregnancy test will be verified against the laboratory results for urine hCG around the same date of the self-reported pregnancy. Similarly, self-reports of pregnancy loss will be ascertained by medical records (in case of a clinically recognized pregnancy) or by blood and urine tests around the same time point (in cases of early loss <12 weeks' gestation).

Operational definitions for a study outcome events are detailed in Section C.4. The sources of information for study endpoints ascertainment:

a) Live birth – based on hospital records
b) hCG detected pregnancy (implantation) – a self-report of a positive home pregnancy test followed by a spot urine test in the clinic and quantitative hCG evaluations in urine and serum specimens.

c) Early loss of pregnancy (ELP) – hCG pregnancy loss will be defined as a rise of urinary HCG to >0.025 ng/mL for at least 3 consecutive days followed by a decline. The rise and fall of HCG will be evaluated during the 10 days prior to the next menstrual period through day 5 of the next menstrual period. Clinically recognized pregnancy losses will be identified based on self-report followed by confirmation on ultrasound and medical records,
d) Specific pregnancy outcomes: Cesarean section, preeclampsia, gestational age, preterm birth birthweight [small for gestational age], major neonatal complications or death, and severe post-partum maternal morbidity will be determined based on hospital records and medical chart abstraction.

All events will be reviewed and verified by a central outcome review that will be performed by the NICHD Project Officer and the Principal Investigators at the study clinical sites and the Data Coordinating Center.

G2.2 Intention to treat analysis

The primary analysis of the clinical trial will be "intent to treat" (ITT) analysis based on the total cohort of randomized patients. This approach will be applied to the primary endpoint (live birth) as well as designated secondary endpoints. The difference between the incidence rates of live births for the two treatment groups will be examined by a two-sided test based on the standard Z-score (using the normal approximation to the binomial distribution). The common null hypothesis states that the effect of aspirin on the outcome is null compared to the placebo.

The secondary objectives (as described in Section A) with binary endpoints will be analyzed similarly as comparison of proportions. For binary outcomes such as pregnancy, the time to event may be of interest. In this case, survival analysis methods will be applied with the log-rank test for comparison of the two study treatment groups. For continuous outcome such as birthweight either parametric or non-parametric methods will be used as appropriate. If transformations to approximate a normal distribution are feasible, they will be carried out prior to the parametric analysis. The normality of the data will be examined both graphically using Q-Q plots and by formal tests (Wilks- Shapiro test). For data (either on original or transformed scale) assumed to be normally distributed the comparison of the two treatment groups will be carried out by a t-test. Alternatively if a non-parametric test is required, the Wilcoxon-Mann-Whitney test will be used.

G.3 Interim analysis

For a multi-year trial such as this one it is useful to examine the data as it accumulates in order to see if the study should be ended early or to catch any surprising developments. Such interim analysis needs to be planned formally. One of the most widely used procedures for interim analysis of a clinical trial is the α spending function approach of Lan and DeMets.[98-99] This method uses a function of time to specify the rate at which the total Type I error probability is to be spent during the trial interim looks so that this quantity does not exceed the desired overall Alpha level. For the EAGeR trial the spending function corresponding to the O'Brien-Fleming procedure will be used. The O'Brien- Fleming boundaries were chosen since for a given sample size they do not significantly change the overall power and are conservative early in the trial. By

conservative we mean that the proportions of success must be extremely different between the treatment arms to result in statistical significance early in the trial.

For our implementation the rate at which Type I error is spent is a function of elapsed time. We consider live birth as the primary trial endpoint and can expect events to start appearing approximately 10 months after subjects are randomized, that is month 16 of the trial. The accrual period will continue through month 40 while Phase II ends by month 57. Thus, for the interim analysis we consider the time interval of 16-56 month as the period when the primary outcome of live birth accumulates. Realistically though, only interim looks prior to month 40 of the trial will affect future recruitment while looks between 40 and 56 months can only affect decisions regarding continuing active or passive follow-up of subjects already enrolled in the trial.

The following Tables 3 and 4 present results of power calculations for various interim looks plans for the EAGeR trial. These demonstrate the implications of different choices for the number and timing of interim looks. We assume throughout an alpha level of 0.05 and a sample size of 1600 women divided equally to the aspirin and placebo arms of the trial. Previously published studies[100] suggest that for the control group a probability of about 0.70 - 0.80 for live birth given pregnancy (P_{preg}) can be expected. Further, the probability of implantation per cycle ($P_{implant}$) was assumed to be constant and in the range between 0.175 and 0.25. Figure 4 shows the probability of live birth (PLB) considering six cycles of menstruation (including both active and passive follow-up):

$$\mathrm{P}LB = \mathrm{P}_{preg}\, P_{implant} \sum_{c=1}^{6} (1 - P_{implant})^{c-1}$$

Figure 4. Probability of live birth versus implantation probability per menstrual cycle

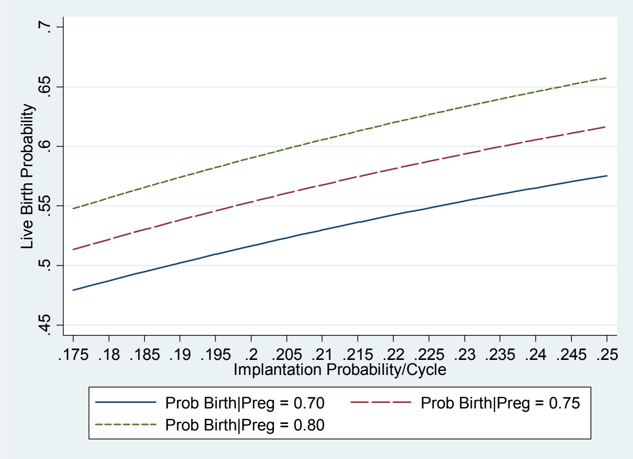

Hence a conservative estimate for the live birth rate would be a combination of implantation rate or 17.5% per cycle and a probability of live birth given pregnancy of 70% yielding an estimated value of PLB = 0.479. The final calculations will be done after the population from which participants are drawn will be identified. A refinement of the calculation can easily incorporate non-fixed probabilities of implantation per cycle

In the following, we consider a range of probabilities for the placebo + folic acid arm of 0.46, 0.48 and 0.50. The effect of the intervention will be measured as the rate ratio (RR) of live birth in the aspirin + folic acid arm compared to placebo + folic acid. We consider effect sizes of 1.15, 1.20 and 1.25. The calculations were preformed using PASS software. Table 3 presents the resulting power for no interim looks and Table 4 shows the power for one or two interim looks for selected outcome probabilities and effect size.

Table 3. Power for comparing live birth rates in treatment and placebo groups with <u>no</u> interim looks.

Live Birth Probability in placebo arm	Live Birth Rate Ratio to be Detected		
	1.15	1.20	1.25
0.46	.77	.96	.99
0.48	.81	**.97**	1.0
0.50	.84	.98	1.0

Table 3 shows for example that with 800 women in each treatment arm, we have >80% power to detect effects ≥ 1.15 for an assumed overall live birth rate of 0.48 in the placebo group.

Table 4 gives the power for one or two interim looks and the Z-score boundaries, assuming PLB = 0.48 in the placebo arm and a rate ratio of 1.20. For the period 16 – 56 months when live births accumulate, an interim look at 0.45 and 0.6 of the way correspond to 18 and 24 months into the planned recruitment period. In other words, stopping at month 18 of recruitment (24th month of the trial) will "save" the last 6 months of recruitment while by 24 months of recruitment (30th month of the trial) the target sample size should be reached and the stopping will apply to cessation of follow-up and data collection. Alternatively if at this stage aspirin is shown to be effective one could switch the placebo patients to aspirin and continue the follow-up. The decision to stop early will not be made solely on the basis of these statistical tests but will be made by the DSMB taking into account all available information including evaluation of secondary outcomes and ethical issues.

Table 4. Power for one or two interim looks assuming PLB = 0.48, RR = 1.2

Number of interim looks	Time of interim looks	Boundaries	Power
1	0.45<br>1.00	$\pm$2.571<br>$\pm$2.010	0.490<br>0.964
1	0.60<br>1.00	$\pm$2.366<br>$\pm$2.038	0.718<br>0.963
2	0.45<br>0.60<br>1.00	$\pm$2.571<br>$\pm$2.485<br>$\pm$2.050	0.490<br>0.696<br>0.962

Thus for example, compared to the naive power of 0.97, one interim look at 0.45 of events accumulation period will results in a very small loss of total power to 0.964

Even with two interim looks there is very little power loss. <u>Two interim looks at 18 and 24 months into the planned recruitment period yield a high total power of 0.962 and is the proposed plan for the trial.</u>

The high power shown for assessing an effect size of RR =1.2 for the primary outcome of the EAGeR trial indicate that more modest effects could be detected with high probability.

The Lan-DeMets methodology is flexible in that the total number and timing of the interim looks does not need to be specified in advance. Based on results of periodic partial analyses additional looks can be planned and the test boundaries can be adjusted according to the spending function.

G.4 Prediction models and secondary analyses

In addition to the ITT analysis the data collected in the EAGeR trial will be utilized to develop prediction models for various endpoints in the gestation and reproductive process. These models will enhance our understanding of these processes and the factors that may affect outcomes. In that, the study will provide important information beyond the evaluation of the effects of LDA.

The prediction models approach is described here only briefly.

Prediction of live birth

Various prediction models can be built for this binary event considering baseline and follow-up information. Since women were randomized to receive one of two treatments, we will initially develop separate models for the groups based on the intention to treat assignment with the population at risk being the complete study population. We propose a few modeling approaches as an example.

A model to predict the probability of live birth may be developed based on the information collected at the baseline visit such as demographic information, medical history, and time since last spontaneous abortion. Three methods can be used to build such a model: (a) Logistic regression (b) Neural networks (c) Regression trees. While logistic regression is a standard technique, the other two methods will allow us to consider more complicated associations between risk factors and outcome (e.g. interactions and optimal cut-off points) as well as discrete outcomes with several possible categories (e.g. early pregnancy loss, pregnancy complications, birth). These aspects may be especially interesting for the EAGeR trial where a number of sequentially occurring endpoints are evaluated.

Validation methods will be used to compare these approaches whereby the data will be divided randomly into a training (e.g. 2/3 of the data) and validation sets (e.g. 1/3 of the data). The models will be fit using the training set and their predictive ability will be compared on the validation set. ROC (receiving operating characteristic) curves will be used to compare the different models and determine which is to be preferred.[101-102] The optimal probability threshold for the logistic and neural network models will be chosen using the Youden Index.[103-105]

In addition to the models using only baseline information there is interest in considering additional clinical and biomarker information gathered during follow-up. In particular, assessing the influence of true exposure to treatment, taking into consideration compliance to assigned treatment and additional aspirin use. Compliance would need to be accounted for either in a cumulative manner (e.g. >80% compliance over time) or by considering lagged variables with compliance being measured over specified time intervals (e.g. trimesters of pregnancy). In contrast to prediction models based on baseline information, such models would be restricted to prediction for subjects who have reached various stages, for example examining the effect of compliance in say the first trimester of pregnancy conditional on completing the first trimester. For these analyses the sample sizes will be lower and models will include covariates. Therefore, specific power analyses will be conducted to assess the power available for testing specific hypotheses of interest.

Other analytic approaches including survival analysis will be applied for secondary endpoints such as time to becoming pregnant or time from hCG detected pregnancy to early pregnancy loss (EPL). Both Cox regression models and neural networks will be considered for covariates adjustment. Finally, for outcomes that may be repeated over time such as symptoms assessed in the safety interview, methods of longitudinal data analysis with repeated measurements will be applied. These include marginal models, both parametric and semiparametric (e.g. generalized estimating equations approach) and random effect models.

One aspect of the study design that will be addressed carefully in the analysis is the subgroup of women who experience an hCG pregnancy loss and return to follow-up with a chance of becoming pregnant again. The rationale for including this group in the primary analysis of live birth is that typically this type of pregnancy loss would not have been detected. However, these women may contribute more than one pregnancy to the analysis (at most two) and up to two losses. These women may be different from other participants in their characteristics or in how aspirin affects them. Therefore sensitivity analysis will be performed to assess the influence of censoring these women at the time of first event on study results.

Secondary outcomes for infants' complications
For these outcomes, expected to be rare, an intention to treat analysis will be performed using Poisson regression methods to compare the overall rate of these (adverse) events in the two treatment groups. Additional analyses will use live-birth as the denominator to calculate rates.

A general note:
In general, for some analyses comparison between study arms will not be based strictly on intention to treat approach. Rather, the denominator will be defined to include only those sub-groups that are at risk for the outcome event according to their course of follow-up. For example, although live birth is the primary outcome and will be analyzed with the ITT principle, we may want to look at live birth rates out of those women who became pregnant to compare the two groups. All these additional analyses will be interpreted with caution to maintain a coherent summary of the effect of LDA on all reproduction and gestation outcomes.

G.5 Evaluation of drop outs and compliance

<u>*Evaluate the effect of drop outs and non-compliance*</u>

The Haifa DCC will co-develop with the Project Officer statistical methods for evaluation of drop out and compliance for the future analyses of the study results. Both drop-outs and non-compliance introduce a problem when the processes do not occur at random. Drop-outs potentially lead to selectively missing data that may bias crude intention-to-treat comparisons. Non-compliance to assigned treatment can also be influenced by intermediate study outcomes and therefore introduce additional bias. Marginal structural models (MSM) suggested in the causal inference framework[106-108] have been used in observational studies to account for time-dependent covariates that are on the pathway to disease outcome or self-selection into treatment.[109-110] MSM have also been useful in

clinical trial for handling non-compliance to provide unbiased estimates of the causal effect of treatment building upon the theory of causal inference and the idea of 'potential outcomes.[111-112] A key component to these models is inverse probability weighting set to model the exposure (e.g. aspirin use) with the end result providing an estimate of the relative risk of the outcome of interest among the treated group compared to placebo, with appropriate confidence intervals.

G6. Analysis for the sub-study on compliance

The sub-study to assess compliance based on laboratory assessments of the aspirin metabolite salicylate (salicylic acid) in urine samples was described above in section D10. The sampling design for this sub-study is somewhat challenging given that little data is available on the pharmacokinetic behavior of LDA metabolites in the urine. Moreover, an important question is how sensitive are salicylate levels of to non-adherence; for example, what is the effect of skipping medication for one day on this biomarker? We propose to perform first a pilot study analyzing a sequential series of daily urine samples for few individuals (on aspirin and also controls) to better understand the trajectory of aspirin metabolites in the urine over time. Compartmental models and other pharmacokinetic analyses will be applied. Based on the analysis of the pilot and the estimated time to steady state and half-life of low-dose aspirin metabolite we will plan the sampling of individuals and times for evaluation of self-reports on adherence.

G.7 Implementation of statistical analysis

Data analysis will be performed on personal computers at the Haifa DCC using SAS, STATA and R software. Questions related to data analysis and methodological issues will be discussed periodically in the weekly staff meetings at the DCC. Interim results will be summarized and presented at the DSMB meetings and discussed with the Project Officer. Holding regular discussions of study analysis is crucially important to ensure that study aims are met and that the interpretation of data is valid. Publication quality summaries of the results of the statistical analyses will be prepared by the Haifa DCC team for interim and final reports, presentations and manuscripts.

H. DATA MANAGEMENT PROCEDURES

H.1. Internet-based clinical trials

The University of Haifa DCC will develop an internet-based system for the EAGeR trial in close collaboration with the investigators and staff members at the Clinical Sites.

Over the last decade increasing numbers of randomized controlled clinical trials as well as other clinical multi-center studies are performed using internet-based technologies. Some of the advantages of such systems include minimization of errors, real time data reporting, saving in resources for recording of the data, less paper and less storage space required.[113-114]

However there are some difficulties that need to be addressed when considering an internet -based clinical trial. The development phase requires time and an understanding of the environment where the system is going to be deployed and its goals. Curley and his colleagues report on such difficulties but note that the system developed for asthma clinical studies is being successfully used.[115] Others have also noted possible difficulties in implementing the new technologies in clinical settings that lack the needed infrastructure or trained staff.[116]

.

H.2. EAGeR web-based system

The web-based system developed for the EAGeR trial is designed to provide an efficient and secure platform for data entry, management, coordination and monitoring of the trial. The system will meet the following goals:

a) Provide a state-of-the-art web-based platform for the EAGeR study to facilitate communications between all study investigators and staff to enhance the study coordination and monitoring.
b) Provide an electronic data capture (**EDC**) system that is simple to use and incorporates edit checks with full screen support.
c) Accessibility – enable the data to be easily retrieved and reorganized according to need.
d) Security – ensure the safety of the data by limiting access to authorized personnel and allowable procedures.
e) Recoverability – devise a backup system that will enable reconstruction of the data to any state at any time to allow for the re-creation of any analysis files and reports that existed in the past.
f) Audit trail – automated audit trail

H.2a Study web-site

The study website will be used for:

1) providing information for potential participants, and study subjects including a brief background on the study and contact information for the clinical sites
2) access to study roster , committees members
3) centralized data handling for participants screening and enrollment
4) obtaining randomized treatment and access the computerized forms
5) links to study documentation including protocol, MOP, forms, codebook
5) access monitoring and progress reports
6) links to newsletter, announcements, meeting minutes.

Access to the study website and the data entry system will be through dial-up, broad-band or wireless connections using available web-browsers.

H.3. Questionnaires and other data forms

All necessary forms and documentation material will be available on-line through the study website and can also be printed on location at the Clinical-sites as needed. The availability of information on line ensures that all are working with the same most updated version

Forms will be designed to be clear, concise, and convenient to use and for the web-based forms also include imbedded coding and initial error checking mechanisms. Data collection for the EAGeR trial will begin with screening, through the baseline visit, active follow-up (with daily diaries and specimens) during the first two menstrual cycles, a passive follow-up during the next four cycles and follow-up during pregnancy. Except for the self-administered daily diaries and certain other questionnaires that require participants to do additional follow-up (family history, medical history, personal information contacts), the principal mode of data collection for the EAGeR trial will be web-based.

All data forms developed will be pre-tested prior to final implementation and modifications made. Questionnaires will include brief specifications and instructions on how to complete them with detailed explanations given in the questionnaire's MOP.

Questionnaires

The study questionnaires and forms are listed in Section D above. Briefly, baseline questionnaires will elicit socio-demographic information, medical and reproductive history and health-related habits. Follow-up visits and phone questionnaires will obtain information on adherence to and safety of Study Pills and brief behavior summaries. Questionnaires are interviewer-administered and entered on a computerized form through the study web system.. A few questionnaires will be given to the participant to be self-administered but all will be reviewed by the study nurse/staff to verify understanding and completion. Paper versions of the questionnaires will be available on the study website in case the access to the web system is not possible. These forms will be entered later with a flag.

Previously completed questionnaires for the same woman will be retrievable from the system permitting on-site checking for consistency of new with old data and allowing the verification of any changes with the participant.

Daily diaries

These will be filled daily by each subject during the active follow-up period of two menstrual cycles, or until pregnancy, whichever comes first and during the first four weeks of identified pregnancy. These forms, eliciting information on treatment compliance, intercourse, exposures to caffeine, tobacco, alcohol and stress, possible adverse effects including bleeding and illnesses are designed to be simple and

convenient to encourage response. The completed forms will be collected by the Clinical Sites according to the study MOP and will then be shipped to the DCC for data entry.

Abstraction forms

Information on the course of pregnancy and delivery will be obtained through abstraction of medical records from routine pre-natal clinic visits, routine tests (e.g. blood tests, ultra-sound, amniocentesis) and any hospitalization, OBGYN, other PMD, or emergency room visits. Chart abstraction forms are formatted and will be entered on the computer web-system similar to other forms.

Biospecimen tracking

For each visit where specimens are being collected a series of barcodes is provided to be printed on labels and placed on tubes and containers per MOP. Each specimen sample before and after processing is barcoded and computerized laboratory forms are used to record the specimen handling and transport. The barcodes contain information on the Study ID number, the date and the type of specimen and tube. Mapping of the repository trays will allow the location of any specific specimen as needed. Shipment from local repository to the central study repository will be documented and performed according to the SOP for the lab specimen and in collaboration with the central repository. The specimen tracking information will be saved in separate data files at the DCC.

a. *Information collected from unscheduled report forms*

In addition to data collected during clinic visits and phone interviews there are a number of forms and reports that are used at any time during follow-up. These include the:

I. Case Report Form (CRF) - used to report any adverse or unusual events
II. Adverse Events Follow-up Form – used to update sequelae of adverse event reported in the CRF
III. Endpoint Report Form – used to report occurrence of study endpoints including pregnancy, loss or termination of pregnancy and delivery
IV. Protocol Violation Form
V. Withdrawal Form – used to report participant's early withdrawal from follow-up or stopping of Study Pills or folic acid
VI. Unscheduled Contact Form
VII. Change Form – used to make any changes in participants information that was entered before on one of the questionnaires.
VIII. Additional Pills/Barcodes Form

Data from each of these unscheduled forms is handled similarly to the visit associated form.

Report of adverse events:

For the EAGeR trial possible adverse events may include spotting or bleeding at anytime during pregnancy, vomiting and other gastrointestinal effects, intrauterine growth restriction, preterm labor, premature preterm rupture of membranes, congenital anomaly, intrauterine fetal death, placental and other birth complications, neonatal death, severe

maternal morbidity requiring hospitalization, and maternal death. It should be noted that these are symptoms that are also generally consistent with pregnancy, therefore may not be unexpected.

If a severe adverse event is identified while the woman is on follow-up, the staff will alert the site PI and complete the CRF within 2 days of becoming aware of the event. The DCC will update the Project Officer and the DSMB to discuss if any action needs to follow, including stopping of Study Pills/folic acid or unblinding to a health care provider.

Other local forms

Other study forms including informed consent and various local administrative form used for local participant management will be developed at the clinical sites in consultation with the DCC.

Data entry procedures

Procedures for data entry are defined and detailed in the MOP. Electronic forms are either a special application (e.g. screening and personal information) or a PDF writable form. Submitted forms need to have a digital signature of the person filling in the information. Information is saved in the study data base and also as a completed PDF form that can be retrieved and serve as a source document.

The Haifa DCC will develop training sessions to guide data entry staff in data entry procedures, coding schemes, and error checking ensuring completeness and accuracy of the data entered. The procedures will include instruction about conducting personal or telephone interviews, recording biospecimen tracking information and abstracted medical information.

Confidentiality procedures

Strict confidentiality procedures ensure that information collected for the EAGeR Trial could not be linked to participant's identifier except for cases where medical attention may be needed. Participants' personal information will only be kept locally at each clinical center and kept separately from the data collected during the study where only the Study ID for each subject will appear. The locator information will be kept in a secured local server with paper forms in a locked secure cabinet/area.

Quality control procedures

The DCC will perform ongoing check of all data entered newly into the database. These will be part of the SAS data management programs and will include checks for discrepancy between information obtained through different forms and/or at different follow-up times.

Ongoing assessments of adherence to the study protocol and evaluations of data quality are essential for the success of the trial. Quality control processes will be established to identify any suspected deviations from what is expected and discrepancies in information obtained from various sources. Routine error and completeness reports will be sent to the clinical center. Those will be returned to the DCC in a timely manner to resolve identified problems and correction of errors. Concise summaries of the findings of the quality control procedures will be presented periodically on the study web-site to alert all study personnel to common problems and suggest resolution as well as any resulting modifications to study protocol.

Quality control for clinical monitoring

The DCC with the NICHD will conduct annual field audits of the clinical sites to assure adherence to the study protocol and requirements. The audits will include examination of the data collection process, specimen collection and processing, data entry procedures, local data management and record keeping. In additional, more frequent audits will be executed by the local project coordinators at each center based on an audit plan developed with the DCC. This will include a sample of about 5% of forms to be entered in parallel by two staff members and checking specimen tracking information.

<u>Data system safety and backups</u>

Data safety

The study servers will be kept in secured locations one in the US and one at the University of Haifa. Access to the study website and database server will be restricted to authorized personnel as determined by the DCC. All transmissions of data over the internet will be encrypted. Servers will be protected by firewall. An automated audit trail will log all entries to the study web-site and the data entry system.

Backup Protocol

The two EAGeR trial study servers are synchronized simultaneously and thus provide a real-time backup for the entire database. Backup procedures will be performed daily for the servers, work stations and the personal computers used for the project. In case of a hardware or software failure, files can be retrieved to the time of their last backup, that is, the previous day. Version for the previous 30 days will be kept. Backup copies will be created on external magnetic media (e.g. tapes, DVD) once a month and global backups, performed every 3 days, will allow retrieval of data up to the previous six months. In addition, the internet-based system creates audit trails with a date-stamp copy of every transaction and therefore the current database can be reconstructed to any state that has occurred since its implementation.

Reports and newsletter

The Haifa DCC will be responsible for preparation and distribution of interim and final reports, quarterly newsletter, presentations and other publications in coordination with

the Project Officer at the NICHD and in collaboration with the study investigators. Reports will include summaries of recruitment and follow-up status, by Clinical Site, graphical and tabular presentations and results of interim statistical analysis. The reports will have clear explanations of the methods used, interpretations and point out questions or problems that need special attention.

Special reports of adverse events occurring during the course of the study will be prepared periodically and submitted to the Project Officer and the DSMB.

The quarterly newsletter with be made available on the study Web-site and contain information on the progress and performance of the clinical centers.

I. HUMAN SUBJECT PROTECTION AND CONFIDENTIALITY PROCEDURES

This section details the issues relevant to protection of human subjects involved in the EAGeR trial.

I.1. Risks and Benefits

A. Risks and Protection Against Risk

1. Procedure Risks

Study Pills and folic acid: Any drug (including aspirin) or dietary supplement (including folic acid) can have harmful side effects, especially when mixed with other products or if the participant is allergic to aspirin or folic acid. Participants who are allergic to the Study Pills or folic acid will be ineligible to participate. Research personnel will monitor participants' pill intake (including supplements and herbal remedies) through phone calls and the information provided in the Daily Diary. Aspirin use can result in serious side effects, such as bleeding ulcers, bleeding in the brain, renal failure, and some kinds of strokes. Some medical conditions, such as hypertension, bleeding disorders, uncontrolled asthma, history of gastrointestinal ulcers, and hepatic and renal disease, could increase the risk of adverse effects from aspirin; therefore, patients with these conditions will be ineligible for participation. Along with its needed effects, a dietary supplement such as folic acid may cause some unwanted effects. Although folic acid does not usually cause any side effects, it can cause fever, general weakness or discomfort, reddened skin, shortness of breath, skin rash or itching, tightness in chest, trouble breathing, and wheezing. These more serious side effects are rare and will be monitored by the research staff through telephone contact and review of Daily Diary entries. If any adverse events are identified, these will be reported to the PI to determine if the participant should discontinue the pills. Any serious adverse events will be reported to the IRB and to the DCC. The DCC will provide periodic summaries of these events to the DSMB.

Blood Sample: This procedure is likely to involve discomfort or minimal pain as well as bruising. Rarely, blood draws include clotting, infection, and fainting. Blood is drawn by certified healthcare personnel in research and/or medical facilities.

Anthropometric Measurements: There are no anticipated physical risks associated with anthropometric measurements. However, there could be some discomfort and/or embarrassment when having the measurements taken. This procedure is conducted by experienced and certified healthcare personnel to minimize this risk.

Home Urine Collection and Fertility Monitor Testing: There are no known risks associated with these procedures. These are standard tests that are easily conducted in the comfort of a participant's home.

Interview: Baseline information will be obtained from each participant addressing her sociodemographic; medical and reproductive history; somatic body type; diet; and

lifestyle. This could be uncomfortable and or embarrassing. This interview will be conducted by trained personnel experienced in interviewing participants.

Daily Diary: Recording information every day can be difficult and frustrating at times. However, the diary has been designed to collect information in a concise fashion to minimize the time spent on this component of the study.

Medical Records Review: The risks associated with this procedure are minimal and include the accidental or incidental disclosure of medical information. This risk is minimized by adhering to strict confidentiality policies (see below).

2. Subject Confidentiality

There is a risk that the participant's information from existing medical records and study data could be released unintentionally. This risk is remote, however, since all research personnel are well-trained (HIPAA and human subject protection training is required) in these issues and will not release information to anyone outside of parameters of the protocol. Data intended to be shared between study sites will be free of identifiers that would permit linkages to individual research participants and variables that could lead to deductive disclosure of the identity.

Study documents/data will be stored in well-established and secure research offices. This information will be stored in locked filing cabinets and on password-protected computers.

3. Subject Privacy

The risk of loss of privacy also exists. However, great consideration is taken when interviewing participants and performing procedures. These interviews and procedures are performed in private and comfortable settings by experienced research staff. By following these safeguards, we anticipate that the risk of compromising a woman's privacy is minimal.

4. Provisions for Intervention in the Event of Adverse Events

All procedures are performed in medical or research centers where emergency assistance is available. Personnel performing study-related procedures are trained and experienced in the procedures they are performing and can identify potential adverse effects.

B. Benefits to Subjects and Others

Women in this study may not realize a direct benefit as a result of their participation. However, during the medical evaluations and interviews, information about their medical care and pregnancy may be discovered that may not have been without participating in

this study. In this event, that information will be relayed to the woman and her referring physician so that management of these issues can be pursued.

It is possible that women may have a more positive pregnancy outcome as a result of participation in this study. However, this cannot be guaranteed.

Information derived from this study will potentially identify a new medical treatment that may significantly impact pregnancy outcomes for many women in the future.

C. Benefits and Importance of the Knowledge to be Gained

Available information shows that low-dose aspirin (LDA) can impact several aspects of reproduction including conception, implantation, early pregnancy loss, late fetal death, preeclampsia, small for gestational age (SGA) fetus, placental problems, and preterm birth. LDA is an ideal therapy; it is apparently safe, widely available, has few maternal side effects, and is inexpensive. The possibility that such a drug could have a major impact on obstetric outcome is very important. However, there is a lot to learn. It is not clear which patients would benefit from LDA, whether other things that can influence blood flow add to the effects of LDA, and what the side effects of this treatment would be. Earlier studies have only focused specific effects of LDA such as in-vitro fertilization outcome or the development of preeclampsia. Reproduction is different in that each stage is linked together. Better blood flow at the time of conception and implantation may ultimately lead to a healthier placenta during pregnancy, and a lower risk of preeclampsia, SGA, and preterm birth.

The potential risks to the participants in this study are relatively low and are reasonable in relation to the importance of the knowledge we anticipate gaining. Procedures outlined in this protocol such as anthropometric measurements, blood draws, ultrasounds, fertility monitor testing, and interviews are minimal and we do not anticipate that the risks are above those anticipated in the course of a person’s daily life.

The protocol clearly identifies risks to the participants and measures have been included to the decrease the risks anticipated in this study. By identifying a new medical intervention that may be associated with positive pregnancy outcomes, these risks are reasonable within the context of the benefits to the participants and women in the future.

D. Alternative Procedures

Since there are no proven medications to minimize adverse pregnancy outcomes, there are no alternative procedures to offer to participants. Women may choose not to participate in this study and continue attempts at conceiving a pregnancy on their own or after consultation with a physician.

I.2. Protection of Human Subjects from Research Risks – Data Coordinating Center Responsibilities

The responsibility of the Data Coordinating Center (DCC) to protect human subjects from research risk will focus on the information aspects. The potential risks involved in this study include clinical ones (e.g. adverse reaction to the aspirin or folic acid), emotional ones (e.g. increased anxiety, embarrassment due to questions related to sexual behavior) and risks to their privacy. The clinical sites have developed a clear informed consent document to make sure that participants understand the study and know the risks involved, however minimal. Participants' personal information, test results and other data will be kept confidential throughout the trial and stored in secured data archives. Personal information will be kept in a locked safe with limited access to specific study staff. Interviews (personal and by telephone) and specimen collection will be conducted in privacy. Personal information is never released without prior permission from participants. Locator information is stored separately from the data identified by participants study ID number. Linking documents are kept secure by authorized staff members. Procedures to release information in case of an adverse event will be established in coordination with the Project Officer and the DSMB.

All DCC personnel will complete human subject protection training. Any harm suffered by a participant, either physical or emotional, or alleged infringement on privacy will be documented and reported to the IRB and to the DSMB.

I.3. Subject Population

A. Women and Minorities

Women 18-40 years of age will be recruited for participation this study. All women who have experienced a pregnancy loss in the last 12 months will be provided with information about the study. In addition, information about the study will be posted in physician offices in the form of "tear-off sheets" or study brochures. No efforts will be made to exclude any women based on minority, racial, or ethnic status. Given the focus of this particular study, women will be the only participants in this study.

Participants will be requested to complete a brief questionnaire at the time of informed consent that identifies ethnicity and racial designation(s). The participant will be asked for ethnicity first and then will be able to identify one more racial designations.

The trial is potentially open to all women who fit the inclusion/exclusion criteria. The DCC will work with the clinical sites to identify potential sources of data to characterize as best as possible the race/ethnicity composition of the population to be screened for participation in the trial. Typically, trial populations do not accurately represent the target population. However, effort will be made to direct the screening to allow a heterogeneous group to be included. The DCC will generate monthly summaries to each site with the age and ethnic composition of the population screened and the group recruited. In case a growing imbalance develops, the issue will be discussed with the clinical sites and with the project officer to come up with necessary changes in recruitment strategies to minimize exclusion of minorities for any reason. No sub-group analysis is planned but statistical modeling of the data will assess the impact of age and race/ethnicity on study endpoints.

B. Children

The EAGeR trial will follow participating women (age 18 or older) throughout pregnancy and obtain information about outcomes including live birth. Although children are not recruited as active participants in the trial, some information on the newborn babies will be collected. This point will be made clear in the consent form. The Haifa DCC and the clinical sites will apply the strict confidentiality procedures that will be implemented for all study data also to information collected on the newborns in the EAGeR trial.

In case a woman agrees to participate in the trial but later refuses to allow any information on the baby to be collected, a note will be made in the woman's file to remind the clinical sites not to collect that information.

Given the scope of this proposal, children under the age of 18 are not appropriate participants since the issues of reproductive health (e.g., miscarriage, fetal death, etc.) are not identified until women have reached adult age. Subjecting children to the risks, although considered minimal in this study, cannot be justified in such a way that would satisfy current DHHS regulations (45 CFR 46.406.).

C. Special Populations and Circumstances

We will not be recruiting and enrolling special classes or participants such as children, prisoners, institutionalized individuals, or others who are likely to be vulnerable populations.

This proposal does not include plans to include such special conditions such as the use of recombinant DNA molecules, human embryonic germ cells, and human embryonic stem cells.

D. Pregnant Women, Fetuses, Placenta

Women are excluded from the study if they are already pregnant. However, since this study is focused on improving the outcome of reproductive health, it will be necessary to follow women if they become pregnant during the course of the study. All stipulations outlined in 45 CFR 46.204 (Research Involving Pregnant Women and 45 CFR 46.206 (Research Involving, After Delivery, the Placenta, the Dead Fetus, or Fetal Material) will be followed to maintain research integrity and protection to the participants.

E. Exclusion if Unable to Consent

Although this is identified in the inclusion/exclusion above, it is important to reiterate that we will not enroll anyone who does not have the ability to provide effective informed consent.

I.4. Confidentiality Procedures

<u>Confidentiality procedures</u>:
To ensure the strict confidentiality of participants' information and data we will establish the following procedures:
a) Study IDs – upon recruitment, each subject will be assigned a study identification number (study ID). This study ID will be used by project staff on all data forms, laboratory specimens and in the study's main databases. In this manner, the identity of the participant will not be linked directly to the data collected during the study. The assignment of ID numbers will be done by the DCC using random components that will make the sequence difficult to predict.
b) A separate locator file will have the identifying information for each subject (including name and address) linked to the study ID number. This file will be kept in a secure place and will only be available to the site personnel, not the DCC.
c) Reports generated based on the collected data will not include any identifying information and thus preserve the confidentiality of the participants.
d) The only occasion where participant identity may be linked to the collected data is in case of an adverse event that requires medical intervention; these cases will be discussed with the Project Officer on a case by case basis.
e) The need to ensure strict confidentiality of the data collected from participants will be stressed as part of the training of the study personnel involved in data collection. The importance of preserving confidentiality as a moral duty of researchers will be explained. The data may include sensitive information about sexual history and contacts and other personal data such as medical history. The willingness of participants to give such information is based on the trust that this data will be used for the research purposes only. Therefore, it is essential that study staff understand this and protects the data collected not only through the study protocols but also information gathered through incidental observations of study participants.
f) The data collected during the study will be presented in an aggregated form as totals (means, medians etc) or percentages and will not include specific individual results.
h) Access to the database will be limited to authorized study staff and will be protected with a changing password.

The DCC will not have any locator information in the study database. All locator information will be located at the local clinical sites. The subjects' code number and identity will be kept in locked files in the research center of the study Principal Investigators. Only key staff and investigators will have access to the records. In order to monitor this research study, representatives from the clinical sites' Institutional Review Boards, and other federal agencies such as NIH (National Institutes of Health), and OHRP (Office of Human Research Protection) may inspect the research records. A certificate of confidentiality will be requested from the federal government and provides further confidentiality protection by authorizing study staff to protect the privacy of subjects in this study.

<u>Data Quality Monitoring</u>: Details of the specific plans for training in data quality monitoring are described briefly in the following paragraph.

The staff members to be trained in data quality monitoring include personnel in the clinical sites involved in collection and entry of data (including the person supervising these activities at each clinical site) as well as the personnel in the Haifa DCC. Staff members involved in data collection and recording need to understand the importance of obtaining full and accurate data as well as understand the reason for each data item collected. Written training materials will be developed by the Haifa DCC staff and used in training sessions together with the procedures manuals. The training programs will follow the outline of the manual. For each form there will be a data entry specification sheet providing specific instruction and references. Each form will be discussed so that the staff is clear about the logical flow of the questions, skip patterns, and content. The training materials will also include practice exercises to familiarize the trainees in various situations and problems that may arise in the process of collecting and recording data. The Haifa DCC will generate sample data that the trainees will need to enter into the computerized forms through the study web-site. These 'made-up' data will include errors and missing information that will need to be detected and resolved for the data to be entered and submitted. Similar training examples will be developed for laboratory specimen data and for abstraction of medical records. Instruction will include options for getting help and resolving data-related problems including emails to the DCC data management team.

As part of quality monitoring, there will be special attention to the uniformity of procedures for data collection across all study sites. At the end of the training session, personnel involved in data collection and entry should demonstrate complete understanding proficiency in the procedures using all data instruments and the study web-site.

In each clinical site, one person will be designated as supervisor of data collection and entry. The supervisor will be trained by the DCC staff in performing routine checks to monitor the adherence to study protocol for data collection and entry.

J. References

1. Wang X, Chen C, Wang L, et al. Conception, early pregnancy loss, and time to clinical pregnancy: a population-based prospective study. Fertil Steril 2003;79:577-84.
2. Zinaman MJ, Clegg ED, Brown CC, et al. Estimates of human fertility and pregnancy loss. Fertil Steril 1996;65:503-9.
3. Evers JH. Female subfertility. Lancet 2002;360:151-8.
4. Wilcox AJ, Weinberg CR, O'Connor JF, et al. Incidence of early loss of pregnancy. N Engl J Med 1988;319:189-94.
5. Boklage CE. Survival probability of human conceptions from fertilization to term. Int J Fertil 1990;35:75-93.
6. Silver RM, Branch DW. Sporadic and recurrent pregnancy loss. In Reece EA, Hobbins JC, eds. Medicine of the fetus and mother. Second edition. Philadelphia: J.B. Lippincott Company, 1999, 195 - 216.
7. Goswamy RK, Williams G, Steptoe PC. Decreased uterine perfusion - a cause of infertility. Hum Reprod 1988;3:955-9.
8. Battaglia C, Larocca E, Lanzani A, et al. Doppler ultrasound studies of the uterine arteries in spontaneous and IVF stimulated ovarian cycles. Gynecol Endocrinol 1990;4:245-50.
9. Steer CV, Mills C, Campbell S, et. al. The use of transvaginal color flow imaging after in vitro fertilization to identify optimum uterine conditions before embryo transfer. Fertil Steril 1992;57:372-6.
10. Abdalla HI, Brooks AA, Johnson MR, et al. Endometrial thickness: A predictor of implantation in ovum recipients? Hum Reprod 1994;9:363-5
11. Norwitz ER, Schust DJ, Fisher SJ. Implantation and the survival of early pregnancy. NEJM. 2001 Nov 8;354(19):1400-8.
12. Goldstein SR. Embryonic death in early pregnancy: a new look at the first trimester. Obstet Gynecol 1994;84:294-297.
13. Klein J, Stein Z. Epidemiology of chromosomal anomalies in spontaneous abortion: prevalence, manifestation and determinants. In: Bennett MI, Edmonds DK, eds. *Spontaneous and recurrent abortion.* Oxford, UK: Blackwell Scientific Publications, 1987:29-50.
14. Levine JS, Branch DW, Rauch J. The antiphospholipid syndrome. N Engl J Med 2002;346:752-63.
15. Silver RM, Draper ML, Lyon JL, Byrne JLB, Ashwood EA, Branch DW: Unexplained elevations of maternal serum alpha-fetoprotein in women with antiphospholipid antibodies: A harbinger of fetal death. Obstet Gynecol, 1994;83:150-5
16. Van-Horn JT, Craven C, Ward K, Branch DW, Silver RM. Histologic features of gestational tissues from women with antiphospholipid and antiphospholipid-like syndromes. Placenta 2004;25:642-8.
17. Parrazzini F, Acaia B, Faden D, et al. Antiphospholipid antibodies and recurrent abortion. Obstet Gynecol 1991;77:854-858.
18. Parke AL, Wilson D, Maier D. The prevalence of antiphospholipid antibodies in women with recurrent spontaneous abortion, women with successful pregnancies, and women who have never been pregnant. Arthritis Rheum 1991;34:1231-1235.

19. Kujovich JL. Thrombophilia and pregnancy complications. Am J Obstet Gynecol 2004;191(2):412-24.
20. Rey E, Kahn SR, David M, Shrier I. Thrombophilic disorders and fetal loss: a meta-analysis. Lancet 2003;361(9361):901-8.
21. Oshiro BT, Silver RM, Scott JR, Yu H, Branch DW. Antiphospholipid antibodies and fetal death. Obstet Gynecol 1996;87:489-93.
22. Branch DW, Silver RM, Blackwell JL, et al. Outcome of treated pregnancies in women with antiphospholipid syndrome: an update of the Utah experience. Obstet Gynecol 1992;80:614-620.
23. Infante-Rivard C, Rivard GE, Yotov WV, et al. Absence of association of thrombophilia polymorphisms with intrauterine growth restriction. N Engl J Med 2002;347:19-25.
24. Livingston JC, Barton JR, Park V, et al. Maternal and fetal inherited thrombophilias are not related to the development of severe preeclampsia. Am J Obstet Gynecol 201;185:153-7.
25. Steel SA, Pearce JM, McParland P, Chamberlain GVP. Early Doppler ultrasound screening in prediction of hypertensive disorders of pregnancy. Lancet 1990;335:1548-51.
26. Bewley S, Cooper D, Campbell S. Doppler investigation of uteroplacental blood flow resistance in the second trimester: A screening study for preeclampsia and intrauterine growth retardation. Br J Obstet Gynaecol 1991;98:871-9.
27. Frusca T, Soregaroli M, Valamonico A, et al. Doppler velocimetry of the uterine arteries in nulliparous women. Early Hum Dev 1997;48:177-85.
28. Chien PF, Arnott N, Gordon A, et al. How useful is uterine artery Doppler flow velocimetry in the prediction of preclampsia, intrauterine growth retardation and fetal death? An overview. Br J Obstet Gynaecol 2000;107:196-208.
29. Salafia CM, Pezzullo JC, Lopez-Zeno JA, et al. Placental pathologic features of preterm preeclampsia. Am J Obstet Gynecol 1995;173:1097-105.
30. Moldenhauer JS, Stanek J, Warshak C, Khoury J, Sibai B. The frequency and severity of placental findings in women with preeclampsia are gestational age dependent. Am J Obstet Gynecol 2003;189:1173-7.
31. Genbacev O, Zhou Y, Ludlow JW, Fischer SJ. Regulation of human placental development by oxygen tension. Science 1997;277:1669-72.
32. Meekins JW, Pijnenborg R, Hanssens M, et al. A study of placental bed spiral arteries and trophoblast invasion in normal and severe preeclamptic pregnancies. Br J Obstet Gynaecol 1994;101:669-74.
33. Vane JR, Botting RM. The mechanism of action of aspirin. Thromb Res 2003;110:255-8.
34. Patrono C, Coller B, Dalen JE, et al. Platelet-active drugs: the relationships among dose, effectiveness, and side effects. Chest 2004;119:39S-63S.
35. FitzGerald GA, Oates JA, Hawiger J, et al. Endogenous biosynthesis of prostacyclin and thromboxane amd platelet function during chronic administration of aspirin in man. J Clin Invest 1983;71:676-88.
36. Patrono C, Garcia-Rodriguez LA, Landolfi R, Baigent C. Low-dose aspirin for the prevention of atherothrombosis. N Engl J Med 2005;353:2373-83.
37. Landolfi R, Gennaro LD, Novarese L, Patrono C. Aspirin for the control of platelet activation and prevention of thrombosis in essential thrombocythemia and polycythemia

vera: current insights and rationale for future studies. Semin Thromb Hemost 2006;32:251-9.
38. CAST (Chinese Acute Stroke Trial) Collaborative Group. CAST: randomized placebo controlled trial of early aspirin use in 20,000 patients with acute ischaemic stroke. Lancet 1997;349:1641-9.
39. Erkan D, Merrill JT, Yazici Y, et al. High thrombosis rate after fetal loss in antiphospholipid syndrome: effective prophylaxis with aspirin. Arthritis Rheum 2001;44:1466-7.
40. CLASP Collaborative Group. Low does aspirin in pregnancy and early childhood development: follow up of the collaborative low dose aspirin study in pregnancy. Br J Obstet Gynaecol 1995;102:861-8.
41. Knight M, Duley L, Henderson-Smart DJ, King JF. Antiplatelet agents for preventing and treating preeclampsia. Cochrane Database Syst Rev 2000;CD000492.
42. Duley L, Henderson-Smart D, Knight M, King J. Antiplatelet drugs for prevention of preeclampsia and its consequences: systematic review. BMJ 2001;322:329-33.
43. Norton M, Merril J, Cooper B, et al. Neonatal complications after the administration of indomethacin for preterm labor. N Engl J Med 1993;329:1602-7.
44. Souter D, Harding J, McCowan L, et al. Antenatal indomethacin: adverse fetal effects confirmed. Aust N Z J Obstet Gynaecol 1998;38:11-6.
45. Loe SM, Sanchez-Ramos L, Kaunitz A. Assessing the neonatal safety of indomethacin tocolysis. Obstet Gynecol 2005;106:173-9.
46. Kozer E, Nifkar S, Costei A, et al. Aspirin consumption during the first trimester of pregnancy and congenital anomalies: A meta-analysis. Am J Obstet Gynecol 2002;187:1623-30.
47. Werler MM, Sheehan JE, Mitchell AA. Maternal medication use and risks of gastroschisis and small intestinal atresia. Am J Epidemiol 2002;155:26-31.
48. Norgard B, Puho E, Czeizel A, et al. Aspirin use during early pregnancy and the risk of congenital abnormalities: A population based case-control study. Am J Obstet Gynecol 2005;192:922-3.
49. Li DK, Liu L, Odouli R. Exposure to non-steroidal anti-inflammatory drugs during pregnancy and risk of miscarriage: population based cohort study. BMJ 2003;327:
50. Vermillion ST, Robinson CJ. Antiprostaglandin drugs. Obstet Gynecol Clin N Am 2005;32:501-17.
51. Silver RM, Edwin SS, Trautman MS, et al. Bacterial lipopolysaccharide mediated fetal death: Production of a newly- recognized form of inducible cyclooxygenase (COX-2) in murine decidua in response to lipopolysaccharide. J Clin Invest, 1995;95:725-31.
52. Peruzzi L, Gianoglio B, Porcellini MG, et al. Neonatal end-stage renal failure associated with maternal ingestion of cyclo-oxygenase-type-2 selective inhibitor. Lancet 1999;354:1615.
53. Landau D, Shelef I, Polacheck H, et al. Perinatal vasoconstrictive renal insufficiency associated with maternal nimesulide use. Am J Perinatol 1999;16:441-4.
54. Wada I, Hsu CC, Williams G, Macnamee MC, Brinsden PR. The benefits of low-dose aspirin therapy in women with impaired uterine perfusion during assisted conception. Hum Reprod 1994;9:1954-7.
55. Kuo Hong-Chang, Hsu Chao-Chin, Wang Shan-Tair, Huang Ko-En. Aspirin improves uterine blood flow in the peri-implantation period. J Formos Med Assoc 1997;96:253-7.

56. Rubinstein M, Marazzi A, Polak de Fried E. Low-dose aspirin treatment improves ovarian responsiveness, uterine and ovarian blood flow velocity, implantation, and pregnancy rates in patients undergoing in vitro fertilization: a prospective, randomized, double-blind placebo-controlled assay. Fertil Steril 1999;71:825-9.
57. Li D-K, Liu L, Odouli R. Exposure to non-steroidal anti-inflammatory drugs during pregnancy and risk of miscarriage: population based cohort study. BMJ 2003;327:368
58. Norman RJ, Wu R. The potential danger of COX-2 inhibitors. Fertil Steril 2004;81:493-4.
59. Hsieh Y-Y, Tsai H-D, Chang C-C, Lo H-Y, Chen C-L. Low-dose aspirin for infertile women with thin endometrium receiving intrauterine insemination: a prospective, randomized study. J Assist Reprod Genet 2000;17:174-7.
60. Duvan CI, Ozmen B, Satiroglu H, Atabekiglu CS, Berker B. Does addition of low-dose aspirin and/or steroid as a standard treatment in nonselected intracytoplasmic sperm injection cycles improve in vitro fertilization success? A randomized, prospective, placebo-controlled study. J Assist Reprod Genet 2006;23:15-21.
61. Birkenfeld a, Mukaida T, Minichiello L, Jackson M, Kase NG, Yemini M. Incidence of autoimmune antibodies in failed embryo transfer cycles. Am J Reprod Immunol 1994;31:65-8.
62. Birdsall MA, Lockwood GM, Ledger WL Antiphospholipid antibodies in women having in-vitro fertilization. Hum Reprod 1996;11:1185-9.
63. Stern C, Chamley L, Norris H, Hale L, Baker HWG. A randomized, double-blind, placebo-controlled trial of heparin and aspirin for women with in vitro fertilization implantation failure and antiphospholipid or antinuclear antibodies. Fertil Steril 2003;80:376-83.
64. Hornstein MD, Davis OK, Massey JB, Paulson RJ, Collins JA. Antiphospholipid antibodies and in vitro fertilization success: a meta-analysis. Fertil Steril 2000;73:330-333.
65. Waldenstrom U, Hellberg D, Nilsson S. Low-dose aspirin in a short regimen as standard treatment in in vitro fertilization: a randomized, prospective study. Fertil Steril 2004;81:1560-4.
66. Peaceman AM, Rehnberg KA. The effect of immunoglobulin G fractions from patients with lupus anticoagulant on placental prostacyclin and thromboxane production. Am J Obstet Gynecol 1993;169:1403-6.
67. Peaceman AM, Rehnberg KA. The effect of aspirin and indomethacin on prostacyclin and thromboxane production by placental tissue incubated with immunoglobulin G fractions from patients with lupus anticoagulant. Am J Obstet Gynecol 1995;173:1391-6.
68. Empson M, Lassere M, Craig JC, Scott JR. Recurrent pregnancy loss with antiphospholipid antibody: A systematic review of therapeutic trials. Obstet Gynecol 2002;99:135-44.
69. Empson M, Lassere M, Craig J, Scott J. Prevention of recurrent miscarriage for women with antiphsopholipid antibody or lupus anticoagulant. The Cochrane Database of Systematic Reviews 2005, Issue 2:CD002859.pub2.
70. Cowchock S, Reece EA. Do low-risk pregnant women with antiphospholipid antibodies need to be treated? Am J Obstet Gynecol 1997;176:1099-100.
71. Pattison NS, Chamley LW, Birdsall M, et al. Does aspirin have a role in improving pregnancy outcome for women with the antiphospholipid syndrome? A randomized controlled trial. Am J Obstet Gynecol 2000;183:1008-12.

72. Tulppala M, Marttunen M, Soderstrom-Anttila V, et al. Low dose aspirin in prevention of miscarriage in women with unexplained or autoimmune related recurrent miscarriage: Effect on prostacyclin and thromboxane A2 production. Hum reprod 1997;12:1567-72.
73. Farquharson RG, Quenby S, Greaves M. Antiphospholipid syndrome in pregnancy: A randomized controlled trial of treatment. Obstet Gynecol 2002;100:408-13.
74. Di Nisio M, Peters L, Middeldorp S. Anticoagulants for the treatment of recurrent pregnancy loss in women without antiphospholipid syndrome. Cochrane Database Syst Rev 2005;CD004734.
75. Gris JC, Mercier E, Quere I, et al. Low molecular weight heparin versus low-dose aspirin in women with one fetal loss and a constitutional thrombophilic disorder. Blood 2004;103:3695-99.
76. Rai R, Backos M, Baxter N, Chilcott I, Regan L. Recurrent miscarriage-an aspirin a day? Hum Reprod 2000;15:2220-3.
77. Frias AE, Luikenaar RA, Sullivan AE, et al. Poor obstetric outcome in subsequent pregnancies in women with prior fetal death. Obstet Gynecol 2004;104:521-6.
78. Branch DW, Silver RM. Criteria for antiphospholipid syndrome: early pregnancy loss, fetal loss, or recurrent pregnancy loss? Lupus 1996;5:409-13.
79. Bose P, Black S, Kadyrov M, et al. Heparin and aspirin attenuate placental apoptosis in vitro: Implications for early pregnancy failure. Am J Obstet Gynecol 2005;192:23-30.
80. Chavarria ME, Lara-Gonzalez L, Gonzalez-Gleason A, et al. Prostacyclin/thromboxane early changes in pregnancies that are complicated by preeclampsia. Am J Obstet Gynecol 2003;188:986-92.
81. Vainio M, Riutta A, Koivisto AM, Maenpaa J. Prostacyclin, thromboxane A and the effect of low-dose ASA in pregnancies at high risk for hypertensive disorders. Acta Obstet Gynecol Scand 2004;83:1119-23.
82. Yu Y, fan J, Chen XS, et al. Differential impact of prostaglandin H synthase 1 knockdown on platelets and parturition. J Clin Invest 2005;115:986-95.
83. Duley L, Henderson-Smart DJ, Knight M, King JF. Antiplatelet agents for preventing preeclampsia and its complications. The Cochrane Database of systematic reviews 2003, Issue 4:CD004659.
84. Heyborne KD. Preeclampsia prevention: Lessons from the low-dose aspirin therapy trials. Am J Obstet Gynecol 2000;183:523-8.
85. Coomarasamy A, Honest H, Papaioannou S, Gee H, Khan KS. Aspirin for prevention of preeclampsia in women with historical risk factors: A systematic review. Obstet Gynecol 2003;101:1319-32.
86. Coomarasamy A, Braunholtz D, Song F, Taylor R, Khan KS. Individualizing use of aspirin to prevent preeclampsia: a framework for clinical decision making. BJOG 2003;110:882-8.
87. Newnham JP, Godfrey M, walters BJN, Phillips J, Evans SF. Low dose aspirin for the treatment of fetal growth restriction: A randomized controlled trial. Aust NZ J Obstet Gynaecol 1995;35:4:370-4.
88. Low dose aspirin in prevention and treatment of intrauterine growth retardation and pregnancy induced hypertension. Italian study of aspirin in pregnancy. Lancet 1993;341:396-400.

89. CLASP: a randomized trial of low-dose aspirin for the prevention and treatment of preeclampsia among 9364 pregnant women. CLASP (Collaborative Low-dose Aspirin Study in Pregnancy) Collaborative group. Lancet 1994;343:619-29.
90. Papageorghiou AT, Roberts N. Uterine aretrey Doppler screening for adverse pregnancy outcomes. Curr Op Obstet Gynecol 2005;17:584-90.
91. Baschat AA. Pathophysiology of fetal growth restriction: implications for diagnosis and surveillance. Obstet Gynecol Surv 2004;59:617-27.
92. Bower SJ, Harrington KF, Schucter K, McGirr C, Campbell S. Prediction of preeclampsia by abnormal uterine Doppler ultrasound and modification by aspirin. BJOG 1996;103:625-9.
93. Vainio M, Kujansuu E, Iso-Mustajarvi M, Maenpaa J. Low dose acetylsalicylic acid in prevention of pregnancy-induced hypertension and intrauterine growth retardation in women with bilateral uterine artery notches. BJOG 2002; 109:161-7.
94. Ebrashy A, Ibrahim M, Marzook A, Yousef D. Usefulness of aspirin therapy in high-risk pregnant women with abnormal uterine artery Doppler ultrasound at 14-16 weeks pregnancy: randomized controlled clinical trial. Croat Med J 2005;46:821-5.
95. Morris JM, Fay RA, Ellwood D, Cook CM, Devonald KJ. A randomized controlled trial of aspirin in patients with abnormal uterine artery blood flow. Obstet Gynecol 1996;87:74-8.
96. Gofinet F, Aboulker D, Paris-Llado J, et al. Screening with a uterine Doppler in low risk pregnant women followed by low dose aspirin in women with abnormal results: a multicenter randomized controlled trial. BJOG 2001;108:510-8.
97. Subtil D, Goeusse P, Houfflin-Debarge V, et al. Randomised comparison of uterine artery Doppler and aspirin (100 mg) with placebo in nulliparous women: the Essai Regional Aspirin Mere-Enfant study (Part 2). BJOG 2003;110:485-91.
98. Lan KKG and and DeMets DL. Discrete sequential boundaries for clinical trials. Biometrika 1983;70:659-63.
99. Demets DL and Lan KKG. Interim analysis – the Alpha-spending function approach. Stat Med 1994;13:1341-52.
100. Weinberg CR and Wilcox AJ. Reproductive epidemiology in modern epidemiology by Rothman KJ and Greenland S. 2nd edition. Lippincott-Raven, Philadelphia; 1998:585-608.
101. Ennis MG, Hinton D, Naylor M, Revow and Tibshirani R. A comparison of statistical learning methods on the GUSTO database. Statistics in Medicine 1998;17: 2501-8.
102. Eftekhar B, Mohammad K, Eftekhar Ardebili H, Ghodsi M and Ketabchi E. Comparison of artificial neural network and logistic regression models for prediction of mortality in head trauma based on initial clinical data. BMC Med Inform Decis Mak 2005;5:3.
103. Zhou XH, Obuchowski NA, and McClish DK. Statistical methods in diagnostic medicine. Wiley: New York; 2002.
104. Greiner M, Pfeiffer D and Smith RM. Principles and practical application of the receiver-operating characteristic analysis for diagnostic tests. Prev Vet Med 2000;45:23-41.
105. Fluss R, Faraggi D and Reiser B. Estimation of the Youden Index and its associated cut-off point. Biom J 2005;47:458-72.

106. Robins JM, Hernán MA, and Brumback B. Marginal structural models and causal inference in epidemiology. Epidemiology 2000;11:550-60.
107. Hernán MA, Brumback B and Robins JM. Marginal structural models to estimate the causal effect of zidovudine on the survival of HIV-positive men. Epidemiology 2000;11(5):561-70.
108. Hernán MA, Brumback B, and Robins JM. Marginal structural models to estimate the joint causal effect of nonrandomized treatments. J Am Statist Asoc 2001;96:440-8.
109. Wang C, Vlahov D, Galai N et al. The effect of HIV infection on overdose mortality. AIDS 2005;10;19(9):935-42.
110. Cole SR, Hernán MA, Robins JM et al. Effect of highly active antiretroviral therapy on time to acquired immunodeficiency syndrome or death using marginal structural models. Am J Epidemiol 2003;158:687-94.
111. Pearl J. Causality: models, reasoning, and inference. New York: Cambridge University Press; 2000.
112. Hogan JW and Lancaster T. Instrumental variables and inverse probability weighting for causal inference from longitudinal observational studies. Stat Methods Med Res 2004;13:17-48.
113. Lallas CL, Preminger M, Pearle MS et al. Internet based multi-institutional clinical research: A convenient and secure option. J Urol 2004;171:1880-5.
114. Kuchenbecker J, Burkhard HD, Schmitz K and Behrens-Braumann W. Use of internet technologies for data aquisition in large clinical trials. Telemedicine J and E-Health 2001;7(1):73-6.
115. Curley RM, Evans RL, Kaylor J, Pogask RM and Chinchilli VM. Development and deployment of an internet-based management system for use by the Asthma Clinical Research Network. Control Clin Trials 2001;22:135S-155S.
116. Lopez-Carrero C, Arriaza E, Bolanos E et al. Internet in clinical research based on a pilot experience. Contemp Clin Trials 2005;26:234-43.

K. Appendices – Included as a separate document

Appendix 1: Screening Questionnaire and Detailed Eligibility Questionnaire

Appendix 2: Baseline Questionnaires
- Personal Information Questionnaire
- Demographic Questionnaire
- Health and Reproductive History Questionnaire Part A and Part B
- Family Medical History Questionnaire
- Exercise Questionnaire
- Lifestyle Questionnaire
- Occupation Questionnaire
- Physical Measurements Form

Appendix 3: Previous Event Verification Form

Appendix 4: Active Phase and Pregnancy Diaries

Appendix 5: Safety Interview and Adherence Questionnaire

Appendix 6: Passive Follow-up Questionnaire

Appendix 7: Pregnancy Follow-up Questionnaire and Short Physical Measurement Form

Appendix 8: Recruitment Materials

EAGeR
The Effects of Aspirin in Gestation & Reproduction

Effects of ***A***spirin in ***Ge***station and ***R***eproduction (EAGeR) Study

Amendment to EAGeR Study Protocol, Version 2.1 May 31, 2007

AMENDMENT # 1, August 19th 2007

General description: This amendment reflects changes in the eligibility criteria that have been modified to allow women with more than one spontaneous abortion to participate as well as not placing a time limit on when the pregnancy loss occurred. The rationale for the change was to offer this study to a wider group of women who may benefit from the results of this trial. This change will also widen the target population and increase our recruitment pool. The randomization scheme has been modified to include stratification by the old or modified eligibility criteria.

Modification to section C.1 on page 14: Introduction

The second sentence in the first paragraph was:
"The target population is women aged 18-40 who have had a single spontaneous abortion in the past year and who are actively trying to conceive."

It is changed to:
"The target population is women aged 18-40 who have had one or two pregnancy losses in the past and who are actively trying to conceive."

Modification to section C.2 on pages 14-16: Eligibility criteria

C.2. Eligibility Criteria

The target population is women of childbearing age who experienced one or two spontaneous pregnancy losses in the past and are actively trying to get pregnant. Women must meet the following eligibility criteria to be considered for participation in the EAGeR trial:

Inclusion Criteria:

a Women experiencing one or two pregnancy losses at any point in gestation in the past that were not elective termination(s). At least one of these losses must be well documented by one of the following:

- Sonogram demonstrating anembryonic loss, embryonic loss or fetal death.

- Histologic confirmation of products of conception that were spontaneously passed per vagina or surgically obtained.
- Hospital records of fetus delivery
- Late menses and positive serum hCG or positive urine hCG documented by hospital or clinic records followed by either a negative hospital/clinic pregnancy test or a decline in urinary hCG level over 3 days.*

b No more than five total pregnancies including the pregnancy loss(es).**

c Up to two prior pregnancies that did not end in a loss **

d Presence of intact tubes (both), ovaries (both), and uterus.

e Between 18 and 40 years of age at time of baseline visit

f Regular menstrual periods between 21 – 42 days in length (within the last 12 months). Regular menstrual periods are defined as no more than an 8-day difference between the woman's shortest and longest cycle.

g No more than one missed menses in the past 12 months (other than those missed due to pregnancy or breastfeeding.

h Actively trying to conceive with a male partner and not using contraception by the baseline visit.

i Not currently pregnant at the baseline or randomization visits.

j Ability and willingness to give informed consent.

k Willingness to be randomized and to take daily study pills for 6 months to a possible 15 months.

* Home pregnancy tests without hCG confirmation from a healthcare provider (either serum or urine) will not be accepted.

** Women may have up to two pregnancies beyond 20 weeks that were not losses, two spontaneous pregnancy losses at any time in the past, and up to one therapeutic or elective termination (two therapeutic or elective terminations if no other pregnancies). Ectopic and molar pregnancies would, for the purpose of enrollment, be considered in the same category as therapeutic termination pregnancies. Women with more than two live births or those with more than two losses, regardless of the week of gestation of the loss, are excluded.

Exclusion Criteria:

a Known allergies to aspirin or non-steroidal anti-inflammatory agents.

b Clinical indication for anticoagulant therapy. These include prior or current thrombosis, antiphospholipid syndrome (APS) or known major thrombophilia.

c Clinical indication for chronic use of NSAIDs such as rheumatoid arthritis.

d Indication for additional folic acid supplementation, such as prior infant with neural tube defect (NTD), seizure disorder.

e Medical contraindication to aspirin therapy. These include uncontrolled asthma, nasal polyps, bleeding disorders, or history of gastrointestinal ulcer.

f Presence of major medical disorders (regardless of severity). These include diabetes, hypertension, systemic lupus erythematosus (SLE), untreated or active cancer (any cancer in remission or non-melanoma skin cancer is not included in the exclusion criteria), liver disease, renal disease, rheumatoid arthritis, cardiac disease, pulmonary disease other than mild asthma, neurologic disease requiring medical treatment, uncontrolled hypothyroidism, uncontrolled seizure disorder. Untreated vitamin B_{12} deficiency, severe anemia (Hct < 30%), hemophilia, gout, nasal polyps, among others.

g Currently undergoing/planned use of assisted reproductive techniques during trial (IVF; IUI; Clomid)

h History of infertility or sub-fertility. This includes any of the following:

- No conception after ≥ 1 year of unprotected intercourse and actively trying to conceive.
- Any prior medical treatment for infertility.
- Prior treatment for known pelvic inflammatory disease.
- Known male infertility or sperm abnormality (current partner)
- Known tubal occlusion, anovulation, uterine abnormality, or endometriosis stage III or IV.
- History of polycystic ovarian syndrome.

i Presence of unstable mental disorder. These include bipolar illness, schizophrenia, uncontrolled depression, uncontrolled anxiety disorder.

j Known current or recent alcohol abuse or illicit drug use.

k Current diagnosis of sexually transmitted infection (STI) (temporary exclusion)

Modification to section D.1 on page 23: Recruitment plan summary (first paragraph)

D.1. Recruitment

The recruitment plan for the EAGeR trial will be site-specific.

Recruitment plan summary

Potential study participants will be recruited from multiple sources so as to reach a diverse study population that includes women who do and do not routinely access the health care system. Two main recruitment methods will be employed to reach potential subjects: **clinic-based recruitment** and **community-based recruitment**. Recruitment methods will include attempts to enroll women of various racial and socioeconomic backgrounds.

The main recruitment strategy of this trial will be based on the clinical settings, where women who have had a pregnancy loss are readily identified and approached. These settings will include hospital-based emergency departments, hospital-based ultrasound units, and private obstetrician/gynecology practices. Clinical sites will be sent a letter describing the study objectives, the goals of the trial and the need for subjects, and will be given study brochures and/or study contact forms for use in their office. The brochures will include a description of the study, some basic Q&A and a telephone number with information on how to reach study personnel. Women can either contact study coordinators directly or give consent for study coordinators to contact them.

Modification to section D.5a on pages 26-27: Randomization visit (first paragraph)

D.5a. Randomization Visit (V-RD)

Following the baseline visit, participants will return for the final enrollment and randomization visit. The randomization visit constitutes the first active follow-up visit and its content aside from the randomization is detailed in the next section, ***D.6a.***

Briefly, during this visit, participants will be randomized following the protocol described below, receive their assigned treatment (3 month supply), and receive the testing materials for the study, i.e., fertility monitors, specimen collection kits, and daily diaries. The study staff will review the instructions with the participant again. Randomization will be in the ratio of 1:1, treatment to placebo stratified by eligibility criteria group and clinical center. Participants will be counseled to begin taking treatment daily starting that day. The next active follow-up visit will be scheduled for approximately the mid point of the current menstrual cycle.

Modification to section D.5b on page 27: Randomization Protocol (first paragraph)

D.5b. Randomization Protocol

Randomization of subjects will be carried out to obtain the 1:1 allocation ratio between the treatment and placebo arms. Randomization will be stratified by center and by eligibility criteria group. Two eligibility groups will be considered: group I including all women who have had only one spontaneous abortion in gestation week <20 and within the last 12 months, no more than one live birth in the past and no pregnancy losses occurring on or beyond 20 completed weeks of gestation; group II includes women who do not fit group I but are eligible by the modified criteria. A computer algorithm will create the random assignment to one of the treatment arms based on random permuted blocks design with randomly varied block size.

Modification to Section K. Appendices 1 2 & 3

K. Appendices

The following questionnaires have been revised to reflect the new eligibility criteria:

- Telephone Screening

- Detailed Eligibility
- Previous Event Verification Form
- Health and Reproductive History Questionnaire Part A and Part B

The revised questionnaires will appear on the web site.

EAGeR
The Effects of Aspirin in Gestation & Reproduction

Effects of ***A***spirin in ***Ge***station and ***R***eproduction (EAGeR) Study

Amendment to EAGeR Study Protocol, Version 2.1 May 31, 2007

AMENDMENT # 2, January 30, 2007

RE: Addition of a sub-study titled: A study of novel adipocyte cytokines and the risk of gestational diabetes mellitus

General description: The purpose of this amendment is to change the "Week 16" visit during pregnancy follow-up from a telephone visit to a clinic visit. The new "Week 16" visit will be identical to other pregnancy clinic visits with the same questionnaires administered and same blood and urine samples obtained. Additional text giving the background and rationale for this sub-study is also added to the protocol

This document includes only the sections in the protocol where changes are made.

Modification to section D.8 on page 33: Pregnancy follow-up

On page 34-35, the 5th and 6th paragraphs of this section were:

The University of Utah will perform seven visits during pregnancy based around routinely scheduled prenatal clinic visits whenever possible to minimize the burden on participants. When study visits are completed at the participant's physician's office (rather than the research clinic), all typical visit activities will still be performed except not all the information on the Short Physical Measurements Form may be available. The week 8 pregnancy visit needs to occur at the research clinic since new randomized pill bottles will need to be obtained for the participant. This supply of pills should be enough to cover the period until week 36 of pregnancy. In Utah, the approximate times of the visits will be: week 4, 8, 12, 20, 28, 36 and parturition (See Table 1 below for timing of contacts and activities). The last active or passive follow-up visit where the positive pregnancy test was obtained is considered week 4 of pregnancy. Specimen collection will be obtained at the 8, 12, 20 and 28 week visits. If the 12, 20, and 28 week visits are done at the participant's physician's office and routine labs are to be drawn at that visit, every effort will be made to obtain the study labs at the same time. There will be no specimen collection at the 36 week visit. Utah will perform monthly phone calls at weeks 16, 24, and 32 to conduct the safety interview, adherence assessment, and the monthly pregnancy questionnaire. The study ultrasounds will be performed at separate visits at one of the four research clinic sites and will be conducted at 6 ½ weeks to confirm and date the pregnancy. Utah will also collect placenta at the time of miscarriage (when feasible) and after delivery. Cord blood will also be collected at the time of delivery when feasible.

The University at Buffalo will perform five pregnancy visits at weeks 4, 8, 12, 20, and 28. The last active or passive follow-up visit where the positive pregnancy test was obtained is considered week 4 of pregnancy. Specimen collection will be obtained at the 8, 12, 20 and 28 week visits. A study ultrasound will be performed at 6 ½ weeks

to confirm and date the pregnancy. In between study visits (at weeks 16, 24, 32, and 36), Buffalo will perform monthly phone calls to conduct the safety interview, adherence assessment, and the monthly pregnancy questionnaire.

It is changed to:

The University of Utah will perform eight visits during pregnancy based around routinely scheduled prenatal clinic visits whenever possible to minimize the burden on participants. When study visits are completed at the participant's physician's office (rather than the research clinic), all typical visit activities will still be performed except not all the information on the Short Physical Measurements Form may be available. The week 8 pregnancy visit needs to occur at the research clinic since new randomized pill bottles will need to be obtained for the participant. This supply of pills should be enough to cover the period until week 36 of pregnancy. In Utah, the approximate times of the visits will be: week 4, 8, 12, 16, 20, 28, 36 and parturition (See Table 1 below for timing of contacts and activities). The last active or passive follow-up visit where the positive pregnancy test was obtained is considered week 4 of pregnancy. Specimen collection will be obtained at the 8, 12, 16, 20 and 28 week visits. If the 12, 20, and 28 week visits are done at the participant's physician's office and routine labs are to be drawn at that visit, every effort will be made to obtain the study labs at the same time. There will be no specimen collection at the 36 week visit. Utah will perform monthly phone calls at weeks 24, and 32 to conduct the safety interview, adherence assessment, and the monthly pregnancy questionnaire. The study ultrasounds will be performed at separate visits at one of the four research clinic sites and will be conducted at 6 ½ weeks to confirm and date the pregnancy. Utah will also collect placenta at the time of miscarriage (when feasible) and after delivery. Cord blood will also be collected at the time of delivery when feasible.

The University at Buffalo will perform six pregnancy visits at weeks 4, 8, 12, 16, 20, and 28. The last active or passive follow-up visit where the positive pregnancy test was obtained is considered week 4 of pregnancy. Specimen collection will be obtained at the 8, 12, 16, 20 and 28 week visits. A study ultrasound will be performed at 6 ½ weeks to confirm and date the pregnancy. In between study visits (at weeks 24, 32, and 36), Buffalo will perform monthly phone calls to conduct the safety interview, adherence assessment, and the monthly pregnancy questionnaire.

<u>Modification to Figure 3 page 36: Timeline for participant follow-up during pregnancy</u>

The change: Week 16 visit was changed from a phone visit (yellow arrow) to a clinic visit (red arrow).

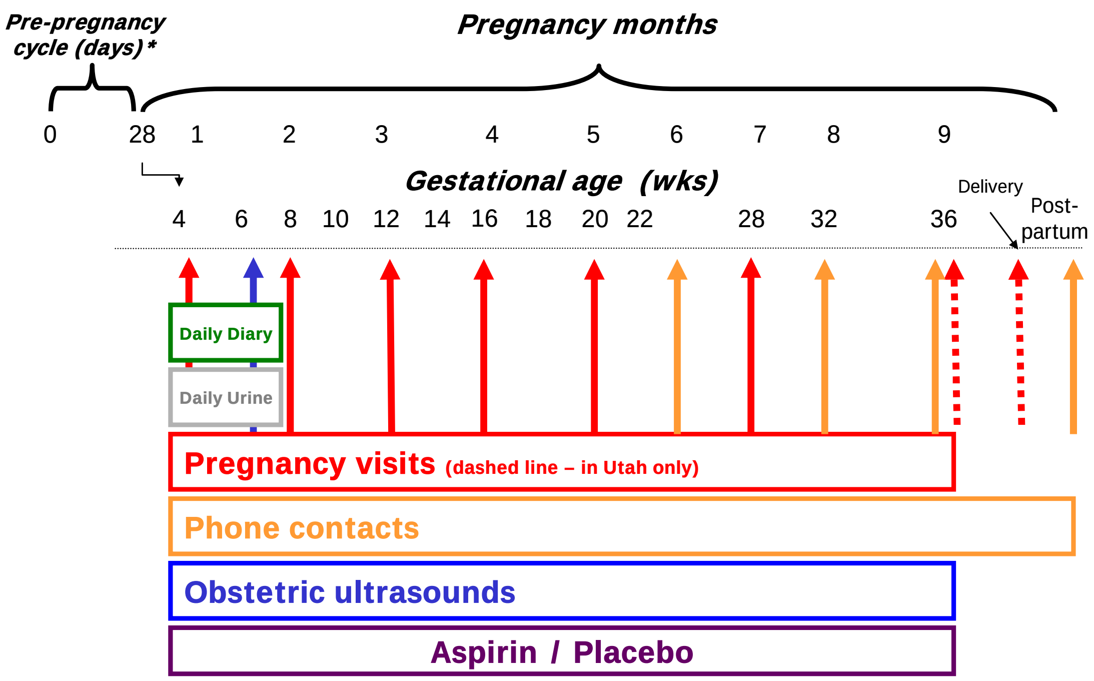

* Day 28 represent the end of cycle visit with a positive pregnancy test

Modification to Table 1 page 36. Activities performed during pregnancy follow-up visits

The change: A new column was added for week 16 pregnancy clinic visit

	Pregnancy Visits								
	Week gestational age								
**Utah & Buffalo**	4	6.5	8	12	16	20	28	36 (Utah)	Parturition
Blood Specimen	X		X	X	X	X	X		X (Utah)
Urine Specimen	X		X	X	X	X	X		
Home Daily Urine Collection	X	→	X						
Pregnancy Daily Diaries	X	→	X						
Study Ultrasound		X				X (Utah)			
Blood Pressure	X		X	X	X	X	X	X	
Anthropometric Measurements	X		X	X	X	X	X	X	
Pregnancy Questionnaire				X	X	X	X	X	
Safety Interview	X		X	X	X	X	X	X	
Adherence Questionnaire	X		X	X	X	X	X	X	
Placenta/Cord Blood Collection									X (Utah)

Modification to section D.11 on page 39: sub-study

The section name was:
D.11 Sub-study

It is changed to:
D.11 Sub-studies

The first paragraph is now a new sub-section D.11.a :

D.11.a "One sub-study will be conducted in the EAGeR trial to evaluate compliance…."

A new sub-section D.11.b. was added to describe the new GDM sub-study:

D.11.b Another sub-study will evaluate the role of adipocyte cytokines in the risk for gestational diabetes. This sub-study is titled: A study of novel adipocyte cytokines and the risk of gestational diabetes mellitus.

Background:
Gestational diabetes mellitus (GDM) is among the most common complications of pregnancy. Approximately 3-7% of all pregnancies are complicated by GDM, resulting in more than 200,000 cases annually (1). Importantly, this number is likely to increase as the prevalence of obesity among reproductive age women increases. Recent data have shown a substantial rise in the incidence of GDM from 1991 to 2000 (2) and a doubling from 1994 to 2002 (3). Women with GDM have increased risk for prenatal morbidity and a considerably elevated risk for impaired glucose tolerance and type 2 diabetes mellitus (type 2 DM) in the years following pregnancy. Children of women with GDM are more likely to be obese and have impaired glucose tolerance and diabetes in early adulthood. Although the precise underlying mechanism is yet to be identified, insulin resistance and inadequate insulin secretion to compensate for it play a central role in the pathophysiology of GDM. The metabolic challenges related to pregnancy reveal an otherwise undetected predisposition to glucose metabolic disorders in some women.

Excess adiposity is the single most important modifiable risk factor for the development of GDM. Mechanisms linking excess adiposity to elevated risk of GDM are not completely understood, but recent evidence points to a crucial role of specific hormones and cytokines ("adipokines") secreted by the adipose tissue. A major breakthrough in understanding the link between adiposity and glucose intolerance has come from the demonstration of a cross-talk between adipose tissue and other insulin target tissues such as skeletal muscle and liver. Such cross-talk is mediated by a number of molecules that are secreted by adipocytes and act in an autocrine, paracrine, or endocrine fashion Among those identified to date are retinol binding protein 4 (RBP4), resistin, leptin, IL-6, and adiponectin (in particular, high molecular weight adiponectin). In concert, these cytokines, or 'adipokines', are believed to adapt metabolic fluxes to the amount of stored energy. Dysregulation of this network is believed to be a critical factor in the deterioration of insulin sensitivity that accompanies adiposity.

Despite the promising role of these novel adipocyte cytokines in the pathophysiology of insulin resistance, prospective studies of these adipokines in association with

GDM risk are sparse. The majority of limited available studies are small cross-sectional studies, inferences from which were hampered due to the ambiguous temporal relation between these adipokines and GDM risk. The major aim of this project is to prospectively investigate novel biochemical markers involved in adipokine secretion as predictors of GDM. In addition, we will examine whether these associations are independent of or modified by measures of overall and abdominal adiposity (BMI and waist circumference), and whether the associations between adipokines and GDM risk are mediated through elevated plasma free fatty acids.

Study design: A nested case-control study of 200 GDM cases and 400 controls based on longitudinal data from 2 prospective studies.

1. EAGeR Trial (PI Dr. Enrique Schisterman): In this study, 1600 women with a history of miscarriages will be recruited. Among them, approximately 960 women (60%) are expected to have successful pregnancy in 6 months of trying. Approximately 40-70 GDM cases are expected to result from this study. For these cases we will select 80-140 controls.

2. The National Standard for Normal Fetal Growth study (PI Dr. Jim Zhang): In this study, 2400 low risk women will be recruited. Among them, there would be 70-90 GDM cases. We will add an arm of 600 high risk pregnant women (being obese) to this study, among which there would be at least 60 GDM cases.

By combining these two studies, we expect to obtain 170-220 GDM cases. Longitudinal blood samples including before pregnancy (EAGeR trial), during the 1st, 2nd, and 3rd trimester and 6 weeks postpartum (Fetal Growth Study) will be collected.

Adding a blood collection in the 16th gestational week to the EAGeR trial will enable the investigation of changes in adipokine levels every four weeks before clinical GDM screening test (24-28 weeks), which is a crucial time window for studying the pathogenesis of GDM. The approximately 4 bio-specimens from each woman before GDM screening test will allow a greater precision in evaluating the longitudinal trend of adipokine levels and other important molecules within individual women. Moreover, the inclusion of 16 gestational week blood collection will enable the time windows for bio-specimen collection in the EAGeR trial to be comparable to those in the Fetal Growth Study, another data source for the 'Adipokines and GDM' project.

Data collection and pregnancy follow-up

The new "Week 16" visit will be identical to other pregnancy clinic visits with the same questionnaires administered and same blood and urine samples obtained. The consent for this sub-study is covered by the original consent.

REFERENCE

1. Gestational diabetes mellitus. *Diabetes Care*. Jan 2004;27 Suppl 1:S88-90.
2. Ferrara A, Kahn HS, Quesenberry CP, Riley C, Hedderson MM. An increase in the incidence of gestational diabetes mellitus: Northern California, 1991-2000. *Obstet Gynecol*. Mar 2004;103(3):526-533.
3. Dabelea D, Snell-Bergeon JK, Hartsfield CL, Bischoff KJ, Hamman RF, McDuffie RS. Increasing prevalence of gestational diabetes mellitus (GDM) over time and by birth cohort: Kaiser Permanente of Colorado GDM Screening Program. *Diabetes Care*. Mar 2005;28(3):579-584.

*E*ffects of *A*spirin in *Ge*station and *R*eproduction
The EAGeR Study

Amendment to EAGeR Study Protocol, Version 2.3, March 17, 2008

AMENDMENT # 3, May 25, 2010

Protocol for Post – Withdrawal Telephone Interview

We plan to contact EAGeR participants who withdraw from the study early to determine if they became pregnant after their withdrawal. This is participants who had no contact with study staff after their withdrawal. Participants will be contacted approximately 10-12 months after withdrawal. These contacts will be implemented in May 2010 and can begin once IRB approval is received. Those who withdrew more than 12 months before May 2010 will be contacted as soon as approval is received.

Procedures: Review each chart carefully before making any contacts with participants who withdrew from the study. If there are any concerns about contacting them, those concerns should be discussed with the PI or on a Conference Call with other EAGeR PIs and staff.

1. If a participant indicates “DO NOT CONTACT FOR ANY REASON,” do not contact.

2. If, in the assessment of the EAGeR team, it would cause undue stress to the participant, do not contact.

3. Place at least three telephone calls at different times of the day, for example, one in the morning, one in the afternoon and one in the evening. Telephone calls may also be attempted on weekends if time and resources allow it.

4. Initial contacts should be attempted by telephone. E-mail contacts are permitted if used while the participant was actively participating, but not as a first method of contact attempt.

The interview will be completed on paper, use the form **Post – Withdrawal Telephone Interview.**

Chapter 1

Interim Analysis

1.1 Preliminaries

1.1.1 Sample size, proportions, and the test statistic

Let $N = N_0 + N_1$ be the trial total sample size, where N_0 is the sample size in the placebo group and N_1 is the sample size in the treatment group. Let n_0, n_1 be the number of completed participants at the time of the interim analysis, in the placebo and treatment groups respectively. Completed participant include all sort of completion except for withdrawal. In the following calculations we assume that $n_0 = n_1$, and define $n = n_0 + n_1$. In particular, we use the notation $t = n/N$ in order to express the time t.

Let p_0 be the proportion of live birth in the placebo group under H_0, and p_1 be the proportion of live birth in the treatment group under H_1. Let $\hat{p}_0(t)$, and $\hat{p}_1(t)$ be the observed proportions of live birth in the placebo and treatment groups at time t respectively. We denote the difference between the proportions by $\delta = p_1 - p_0$, and its estimator by $\hat{\delta}(t) = \hat{p}_1(t) - \hat{p}_0(t)$. Thus, if $n_0 = n_1$, we can use the identity $t = n/N$, and define the test statistic to be

$$Z(t) \quad = \quad \sqrt{t}\frac{\hat{\delta}(t)\sqrt{N}}{2\sigma}, \tag{1.1}$$

where

$$\sigma = \sqrt{\frac{p_1(1-p_1)}{2} + \frac{p_0(1-p_0)}{2}}. \tag{1.2}$$

Group sequential procedures for interim analysis are based on their equivalence to discrete boundary crossing of a Brownian motion process with drift parameter θ. The test statistics $Z(t)$ follow the multivariate normal distribution with means $\sqrt{t}\theta$ and, for $t_1 < t_2$, covariances $\sqrt{t_1/t_2}$. The drift parameter θ is related to the parameters of the z-test through the equation

$$\theta = \frac{\delta\sqrt{N}}{2\sigma}. \tag{1.3}$$

We note that θ is independent of t and is pre-specified in the beginning of the trial.

In the sequel we consider the following scenarios. The total sample size is $N = 1600$, where $N_1 = N_0 = 800$. For the placebo proportion we consider the values $p_0 = 0.4,\ 0.42,\ 0.44,\ 0.46,\ 0.48$. The treatment proportions will be calculated as $p_1 = RR * p_0$, where RR is the relative risk according to the EAGeR protocol. Specifically, we consider $RR = 1.15,\ 1.2,\ 1.25$.

1.1.2 Type one and Type two errors

Under H_0 we have $p_1 = p_0$ so that the drift parameter θ defined in (1.3) is equal zero. Under H_1 we assume that $\theta \neq 0$. The type one error α, and type two error β of the trial are defined as follows.

$$\begin{aligned} \alpha &= P(Z(1) > z_{1-\alpha}|H_0) = P(N(0,1) > z_{1-\alpha}) = 1 - \phi(z_{1-\alpha}), \\ \beta &= P(Z(1) \leq z_{1-\alpha}|H_1) = P(N(\theta,1) \leq z_{1-\alpha}) = \phi(z_{1-\alpha} - \theta), \end{aligned}$$

where ϕ stands for the standard normal distribution. The power of the trial is equal to $1 - \beta$.

According to the EAGeR protocol, in order to calculate boundaries for efficacy, we use the two sided alpha spending function as given by

$$\alpha(t) \;=\; 2 - 2\phi\Big(\frac{z_{1-\alpha/4}}{\sqrt{t}}\Big). \tag{1.4}$$

In particular, the total type one error is set to $\alpha = 0.05$.

Note that the final value for $z_{1-\alpha}$ is determined after we calculate boundaries for efficacy using the alpha spending function displayed in (1.4). In order to emphasize this point, in the sequel we denote the final $z_{1-\alpha}$ by z_F.

1.1.3 Stopping for futility

The conditional power

Denote the Browninan motion value at time t by $B(t)$. Then we have that $B(t) = \sqrt(t)Z(t)$, where $Z(t)$ is given by (1.1). Given that we observe at time t the value $B(t) = b(t)$, the conditional power at time t is define by

$$CP(t,\theta) = P(B(1) > z_F|B(t) = b(t)).$$

Using the Brownian motion framework for our calculations, this probability is equal to

$$\phi(Z_{CP}(t,\theta)), \tag{1.5}$$

where

$$Z_{CP}(t,\theta) = \frac{b(t) + (1-t)\theta - z_F}{\sqrt{1-t}}. \tag{1.6}$$

Remarks.

(1) At the beginnig of the trial $t = 0$, and $b(0) = 0$. Thus, we have

$$CP(0, \theta) = \phi(\theta - z_F) = 1 - \beta,$$

the power of the trial.

(2) The drift parameter θ in (1.6) resembles the effect size from time t until the end of the trial.

(3) Except for t, and θ, the conditional power value is dependent on z_F.

Remark (2) implies that there are other options for calculating the conditional power value. For example, one can assume that the effect size from now on is zero, which results in a different value $CP(t, \theta = 0)$. Also, we can replace the drift parameter θ in (1.6), by its estimator $\hat{\theta} := b(t)/t$ (current trend), which will give us another conditional power value. In what follows, we focus on the conditional power value as given by (1.6).

Probability for stopping for futility

Let us look again at the expression for $Z_{CP}(t, \theta)$ as given by (1.6). If we consider this term before we observe $B(t) = b(t)$, then $Z_{CP}(t, \theta)$ is a random variable, since $B(t) \sim N(t\theta, t)$. In particular, in this case we have

$$Z_{CP}(t, \theta) = \frac{B(t) + (1 - t)\theta - z_F}{\sqrt{1 - t}} \sim N\Big(\frac{t\theta + (1 - t)\theta - z_F}{\sqrt{1 - t}}, \frac{t}{1 - t}\Big). \tag{1.7}$$

Based on the expression above we can calculate the probability of stopping for futility. Suppose that we decide to stop for futility if the conditional power value as given by (1.5) is less than some pre-specified value γ, say 0.3; i.e., we will stop the trial for futility, if at time t we have $CP(t, \theta) = P(B(1) > z_F | B(t) = b(t)) \leq 0.3$. Using (1.7) we can calculate the probability for such an event. Specifically, this probability is given by

$$P_{stop} = P(CP(t, \theta) \leq \gamma) = P(Z_{CP}(t, \theta) \leq z_\gamma). \tag{1.8}$$

According to (1.8), we can specify a boundary value for the Brownian motion for which we will stop the trial for futility. This boundary value is given by

$$b_\gamma(t) := z_\gamma \sqrt{1 - t} - (1 - t)\theta + z_F.$$

Thus, we will stop the trial for futility if $B(t) \leq b_\gamma(t)$. The probability for stopping for futility then takes the form

$$P_{stop} = P(B(t) \leq b_\gamma(t)).$$

Note that here we can compute the stopping probability under the assumption that $B(t) \sim N(0, t)$, which we denote by $P_{stop}(H_0)$. Similarly, we can compute the stopping probability

under the assumption that $B(t) \sim N(t\theta, t)$, which we denote by $P_{stop}(H_1)$. We obtain the following expressions for the probability for stopping for futility.

$$P_{stop}(H_0) = \phi\Big(\frac{b_\gamma(t)}{\sqrt{t}}\Big), \quad (1.9)$$

$$P_{stop}(H_1) = \phi\Big(\frac{b_\gamma(t) - t\theta}{\sqrt{t}}\Big). \quad (1.10)$$

Remarks.

(1) Smaller $B(t)$ values are required to yield low CP values under H_1 than under the current trend or H_0.

(2) Here too, there are other options for calculating the probability for stopping for futility with respect to what we plug in for the drift parameter θ, and in addition, to the expectation of the random variable $B(t)$.

When we stop a trial for futility, we expose ourselves to a type two error. For example, when we have only one interim look for futility stopping (and no other looks for efficacy), the total erros β_T and α_T of the trial are then calculated by

$$\beta_T = P_{H_1}(B(t) \leq b_\gamma(t)) + P_{H_1}(\{B(t) > b_\gamma(t)\} \cap \{B(1) \leq z_F\}),$$
$$\alpha_T = P_{H_0}(\{B(t) > b_\gamma(t)\} \cap \{B(1) > z_F\}).$$

Note that α_T is smaller than the original type one error α. If other interim looks are planed for efficacy, then the calculation will be in the same spirit, but different.

1.2 Sensitivity analysis

The following calculations are based on one interim look for futility, and no looks for efficacy. Thus we set $z_F = z_{1-\alpha/2} = 1.96$.

We first explore a case where we observe no difference between the two arms at time t. In this case we observe $\hat{\delta}(t) = 0$, and the statistic $Z(t)$ given by (1.1) is equal zero. Thus, the conditional power at time t is calculated by plugging $b(t) = 0$ into (1.6). We analyse this case under two extreme scenarios. In the first scenario we let $p_0 = 0.48$, and $RR = 1.2$ so that $p_1 = 0.576$, $\delta = 0.096$, and $\theta = 3.864$. The power of the trial is 97%, and we get

the following plot.

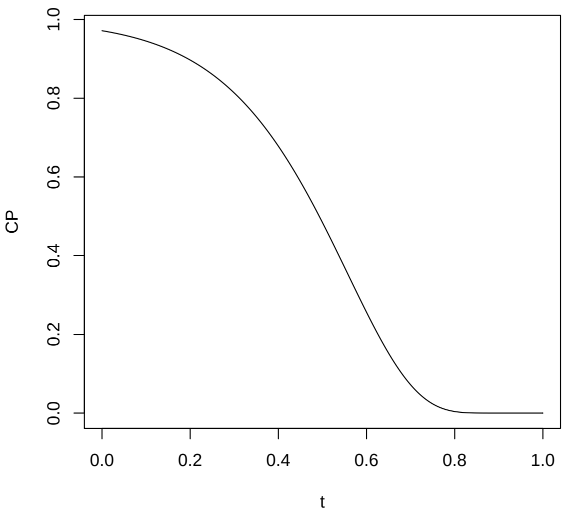

If we decide to stop the trial for futility if the conditional power value is below 30%, then the probabilities for stopping for futility under H_0, H_1, are given by (1.9)-(1.10) and displayed below.

(a) (b)

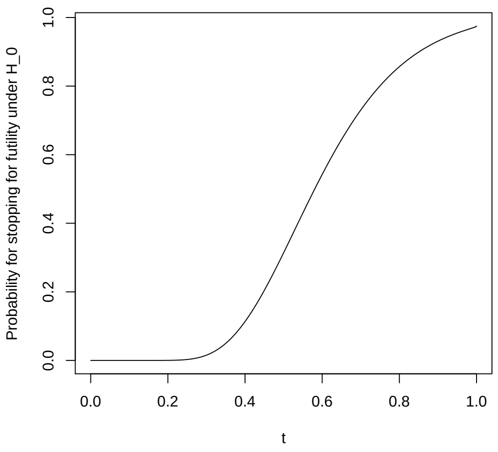

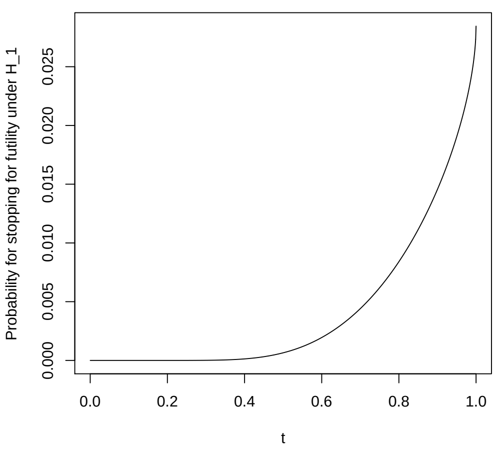

Now suppose that $p_0 = 0.4$, and $RR = 1.15$ so that $p_1 = 0.46$, $\delta = 0.06$, and $\theta = 2.43$. The power of the trial is then 68%, and the conditional power values at time t when no

difference is observed between the two arms is displayed in the plot below.

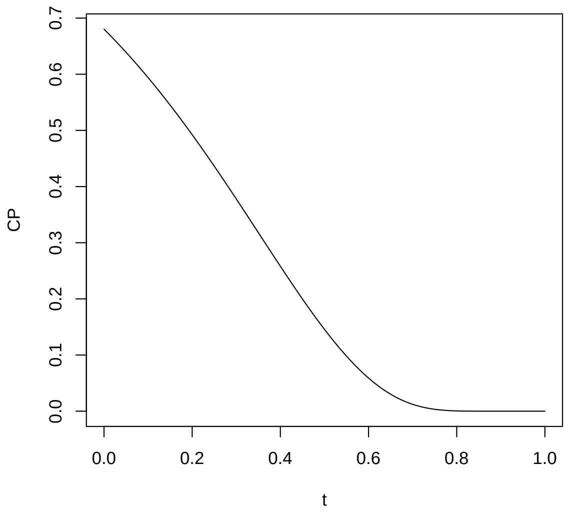

If we decide to stop the trial for futility if the conditional power value is below 30%, then the probabilities for stopping for futility under H_0, and H_1 are displayed below.

(a) (b)

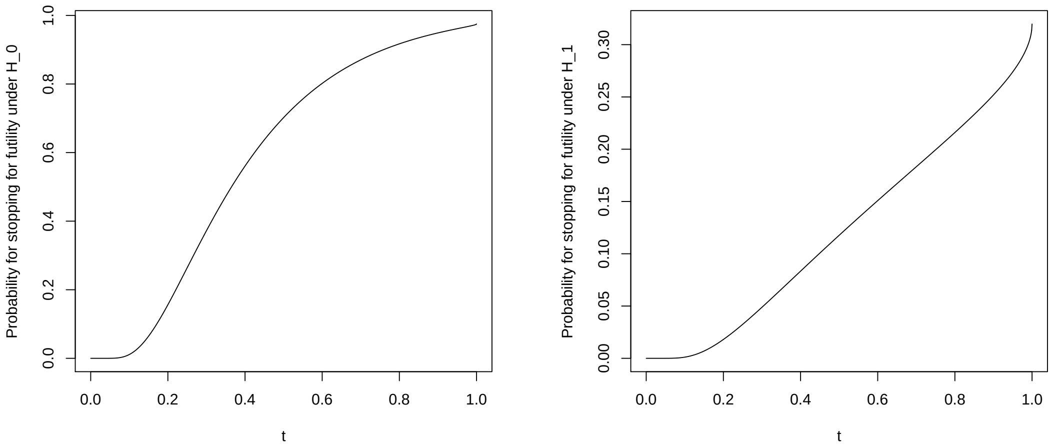

We now explore again the case where we observe no difference between the two arms at time t, for specific values of t, as a function of p_0. In the first scenario we let $t = 0.2$, $p_0 = 0.4,\ 0.42,\ 0.44,\ 0.46,\ 0.48$. The treatment proportions will be calculated as $p_1 = RR * p_0$, where $RR = 1.15$. This yields $p_1 = 0.46,\ 0.483,\ 0.506,\ 0.529,\ 0.552$, $\delta = 0.06,\ 0.063,\ 0.066,\ 0.069,\ 0.072$, and $\theta = 2.43,\ 2.54,\ 2.65,\ 2.77,\ 2.88$. The power of

the trial is 68%, 72%, 75%, 79%, 82%, with respect to p_0, and we get the following plot.

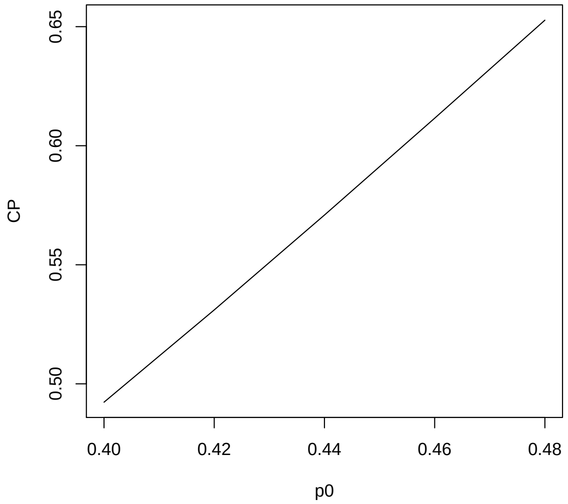

If we decide to stop the trial for futility if the conditional power value is below 30%, then the probabilities for stopping for futility under H_0, H_1, are given by (1.9)-(1.10) and displayed below.

(a)

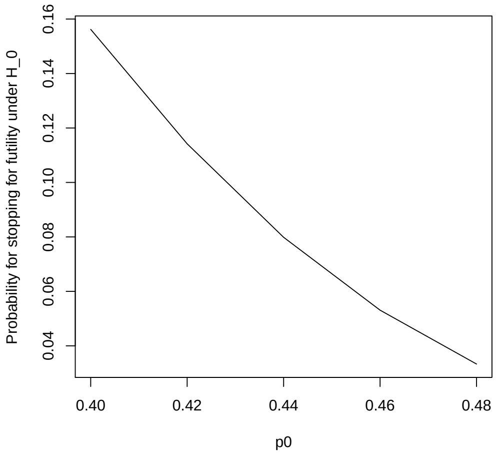

(b)

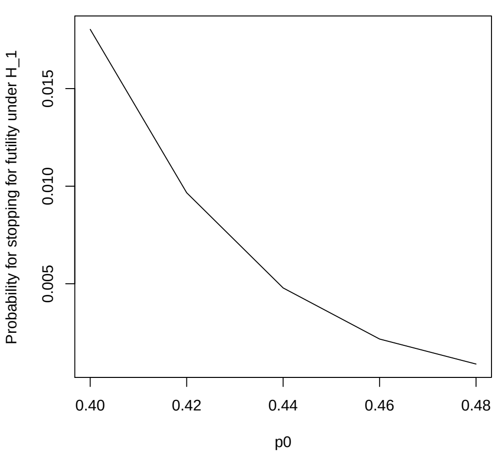

Now we introduce the same scenario for time $t = 0.3$.

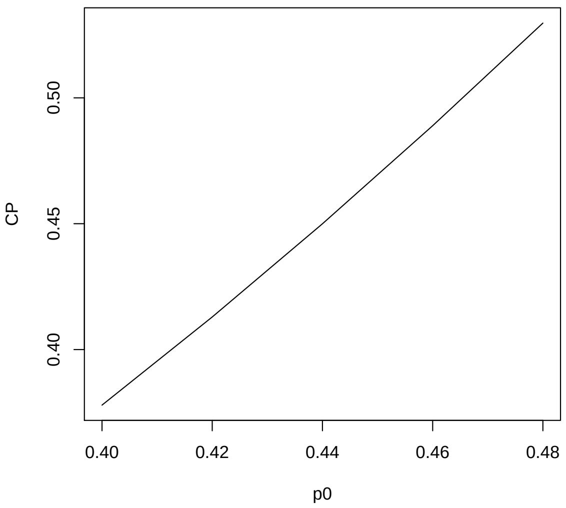

If we decide to stop the trial for futility if the conditional power value is below 30%, then the probabilities for stopping for futility under H_0, H_1, are given by (1.9)-(1.10) and displayed below.

(a)

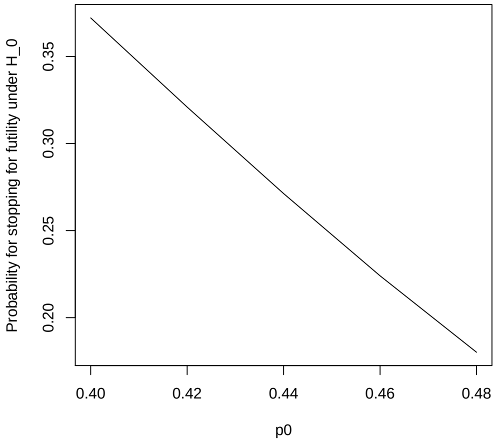

(b)

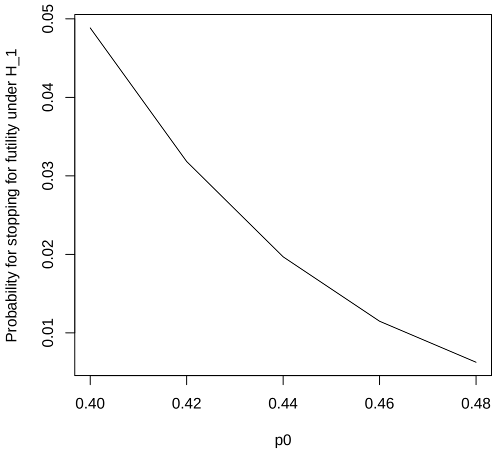

Supplement: S2 File — (PDF) [file pone.0200533.s003.pdf]
